# Supplementary material for: Combining machine learning and nanopore construction creates an artificial intelligence nanopore for coronavirus detection
Source: Nat Commun. 2021 Jun 17;12:3726. doi: 10.1038/s41467-021-24001-2 (PMC8211865; doi:10.1038/s41467-021-24001-2)
Supplement: Supplementary file 1 — Supplementary Information [file 41467_2021_24001_MOESM1_ESM.pdf]

Supplementary Information for
Combining machine learning and nanopore construction creates an artificial intelligence nanopore for coronavirus detection

Masateru Taniguchi, Shohei Minami, Chikako Ono, Rina Hamajima, Ayumi Morimura, Shigeto Hamaguchi, Yukihiro Akeda, Yuta Kanai, Takeshi Kobayashi, Wataru Kamitani, Yutaka Terada, Koichiro Suzuki, Nobuaki Hatori, Yoshiaki Yamagishi, Nobuei Washizu, Hiroyasu Takei, Osamu Sakamoto, Norihiko Naono, Kenji Tatematsu, Takashi Washio, Yoshiharu Matsuura & Kazunori Tomono

The Supplementary Information includes:

1. Identification of nanoparticles (Tables S1-S3, and Figs. S6 and S7)
2. Machine learning algorithm utilized to identify nanoparticles and cultured coronaviruses (Figs. S1-S5)
3. Identification of the four cultured coronaviruses (Figs S8 and S9, and Table S4)
4. Description on the machine learning algorithm used to identify clinical specimens (Figs. S10-S12)
5. Identification of SARS-Co-2 in clinical specimens of saliva (Figs. S13-S15, and Tables S5 and S6)
6. Identification of cultured SARS-CoV-2 and cultured influenza virus (Fig. S16)
7. List of all primers used (Table S7)
8. Supplementary reference

1. Identification of nanoparticles

Materials: Standard nanoparticles of polystyrene with diameters of 200 nm ± 6 nm and 220 nm ± 6 nm are NIST standard certified nanoparticles procured from Thermo Fisher Scientific. Nanopores with diameters of 200 nm and 220 nm were measured with three nanopores with a diameter of 300 nm ± 10 nm (Table S1). The number of ionic current-time waveforms (number of waveforms) obtained by applying a voltage of 0.1 V using 1 x PBS as a buffer is given in Table S2. In order to investigate the nanopore dependence on the discrimination accuracy of the 200 nm and 220 nm nanoparticles, machine learning was conducted according to Figures 1k, S1-S4 to obtain the confusion matrixes and F-value of all combinations of (A, B, C) and (D, E, F) (Figure S5). The F-value ranged from 0.97 to 1.0 for all the combinations.

Table S1 Nanopore modules used to measure the nanoparticles.

Diameter	1st measurement	2nd measurement	3rd measurement
200 nm	A	B	C
220 nm	D	E	F

Table S2 Number of waveforms obtained from nanoparticle measurements and number of waveforms used for machine learning.

Diameter	Number of nanopores for measurements	Extracted waveforms	Waveforms used for AI training
200 nm	3	695	695
220 nm	3	1808	695

Pulses extracted automatically using Aipore-ONE^{TM} extraction software.

2. Machine learning to identify nanoparticles and cultured coronaviruses

Machine learning algorithm was implemented on the waveforms obtained from nanoparticles with diameters of 200 nm and 220 nm, four types of cultured coronavirus, cultured SARS-CoV-2 and cultured influenza as denoted in Figure 1k. Figures S1 and S2 represent a detailed description of Figure 1k. The under sampling method and 10-fold cross validation methods shown in Figure S2 are described in Figures S3 and S4, respectively. The calculated scores are F-value, Recall, and Precision. Each score is defined by Equations 1 to 4 (Figure S5).

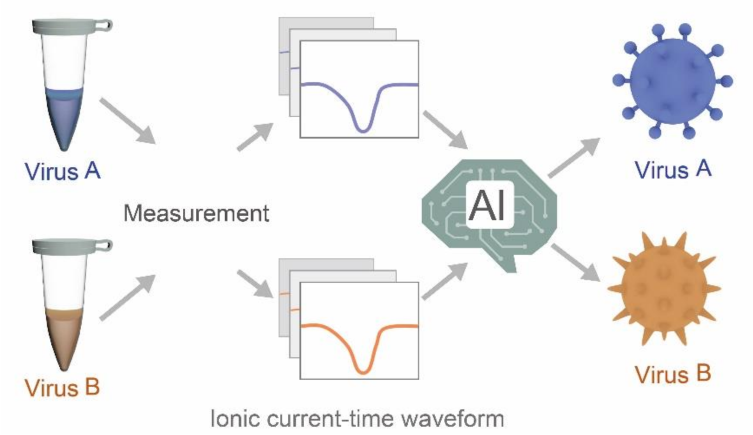

Figure S1. Schematic flowchart implemented on 2-types of nanoparticles and cultured coronaviruses. After acquiring the ionic current-time waveform data of the viruses (nanoparticles) of A and B, the waveform data of A and B are machine-learned to distinguish between A and B. Machine learning is performed until the highest identification accuracy is obtained using the F-value as an index. The F-value is calculated using equations (1), (2), and (4) in Figure S5.

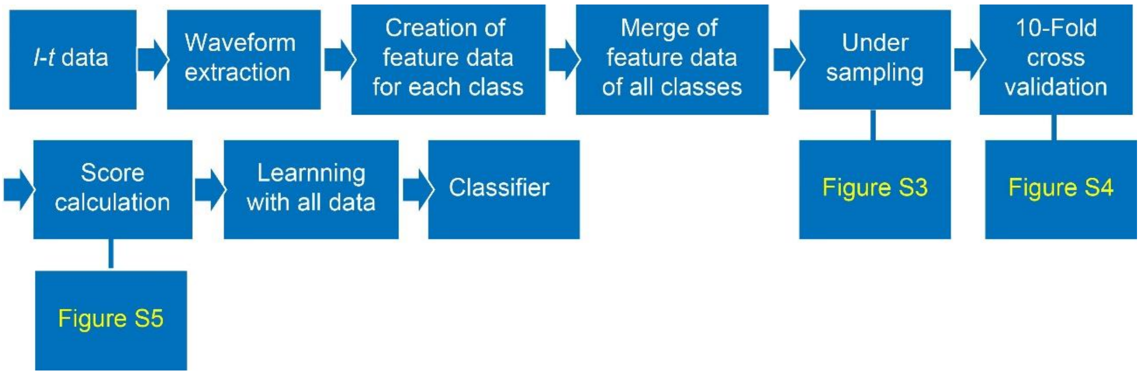

Figure S2 Details on the machine learning algorithm implemented on the nanoparticles and cultured coronaviruses. All the obtained measurement data are transferred to the server and processed as follows. Each waveform is automatically extracted from the measured data by the waveform extraction software installed in Aipore-ONE^{TM}. Features of each nanoparticle or each cultured virus are generated. The features of the two nanoparticles or four cultured viruses are merged. The number of waveforms for each nanoparticle and each virus is adjusted to the minimum number of waveforms to avoid overfitting due to non-uniform data volume. This method is an under sampling method, the outline of which is described in Figure S3. Subsequently, the waveform data is randomly divided into 10 parts; 9 data groups are used for learning and 1 data group is used for testing. As shown in Figure S4, a 10-fold iteration is conducted and the average score is calculated which is defined in Figure S5. In order to improve the accuracy, machine learning procedure is performed using all the under-sampled waveforms. The classifier that gives the highest F-value is considered the most effective to identify the nanoparticles or cultured viruses. Most of the optimized classifiers correspond to the rotation forest and random forest algorithms. Utilizing the popular WEKA software package[1], 71 types of classifiers are implemented in this study.

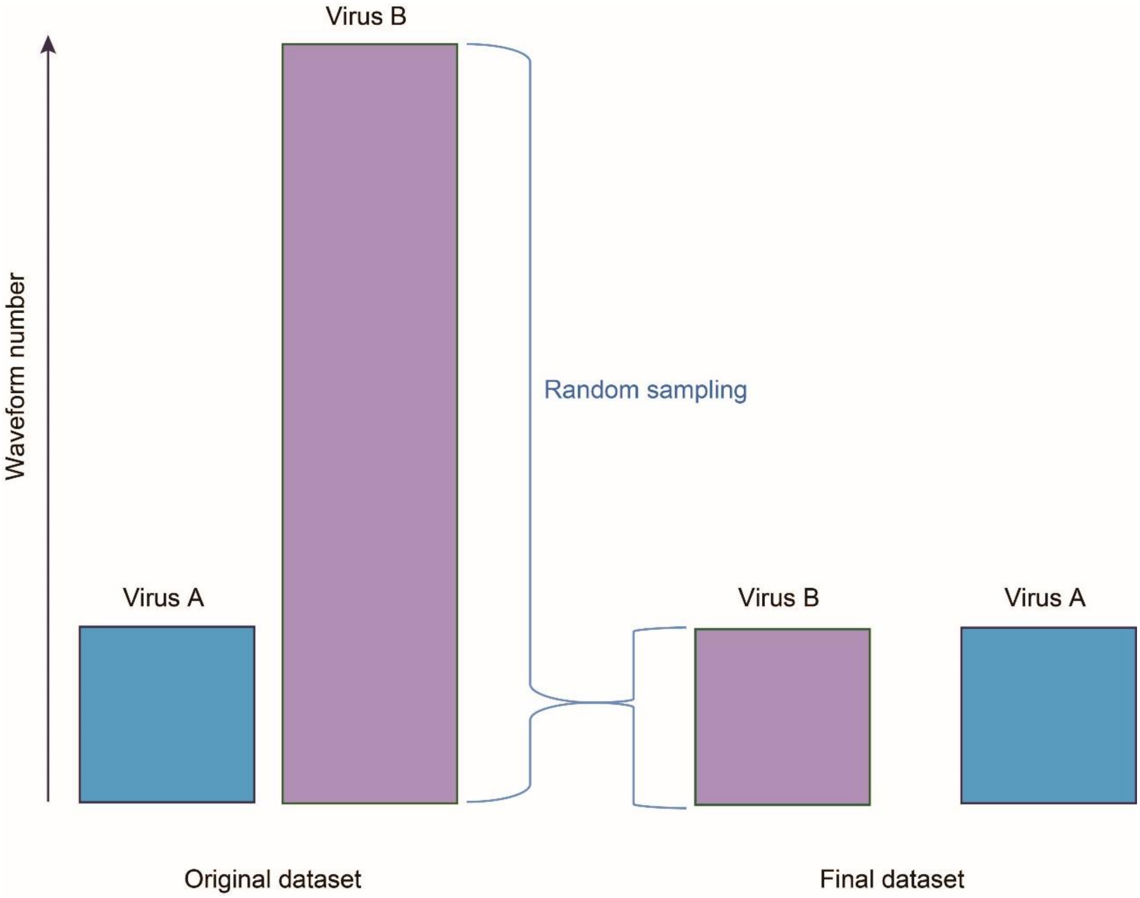

Figure S3 Under sampling method. This method adopts the procedure of aligning the number of waveforms with the sample having the smallest number of waveforms. The method randomly extracts the virus B data and aids in eliminating the bias included while implementing the machine learning algorithm.

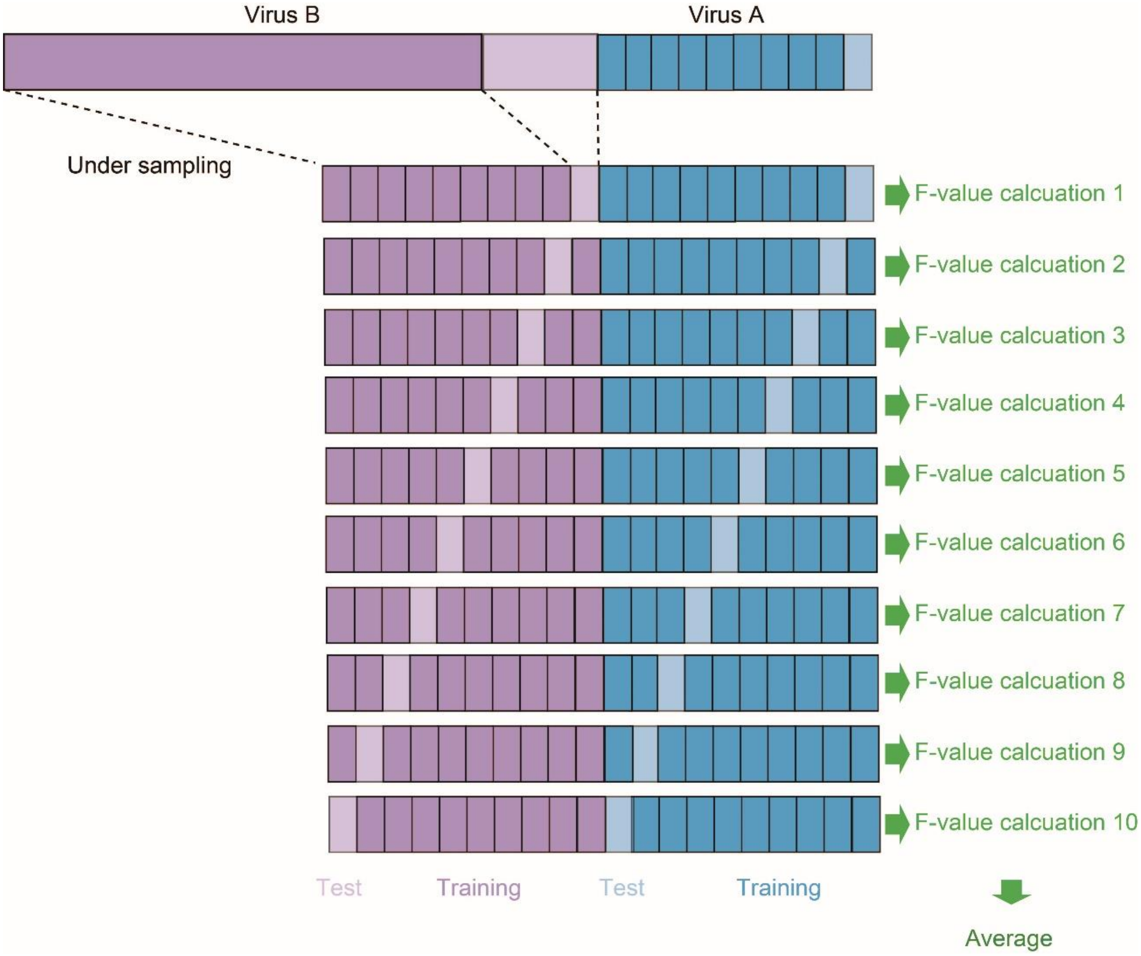

Figure S4 10-Fold cross validation method. In this method, machine learning and identification are performed, and the F-value is calculated. The F-value obtained is the average of 10 values. The data is divided into 10 parts; 9 data groups are used for learning, and 1 data group is used for identification.

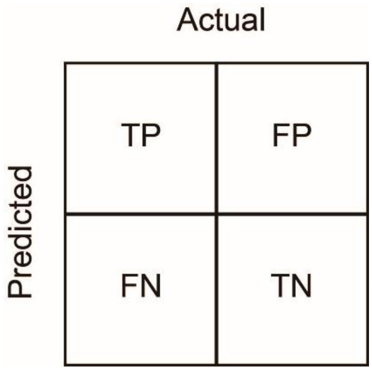

Figure S5 Confusion matrix calculated by machine learning. TP and TN show true positive and true negative, respectively. FP and FN indicate false positive and false negative, respectively. Based on this confusion matrix, the scores defined by equations (1-4) are calculated.

$$\mathrm{Precision}(P) = \frac{\mathrm{TP}}{\mathrm{TP+FP}} \tag{1}$$

$$\mathrm{Recall}(R) = \mathrm{Sensitivity} = \frac{\mathrm{TP}}{\mathrm{TP+FN}} \tag{2}$$

$$\mathrm{Specificity} = \frac{\mathrm{TN}}{\mathrm{FP+TN}} \tag{3}$$

$$\mathrm{F-value} = \frac{2\mathrm{Recall}\times\mathrm{Precision}}{\mathrm{Recall+Precision}} = \frac{2}{\frac{1}{\mathrm{P}}+\frac{1}{\mathrm{R}}} \tag{4}$$

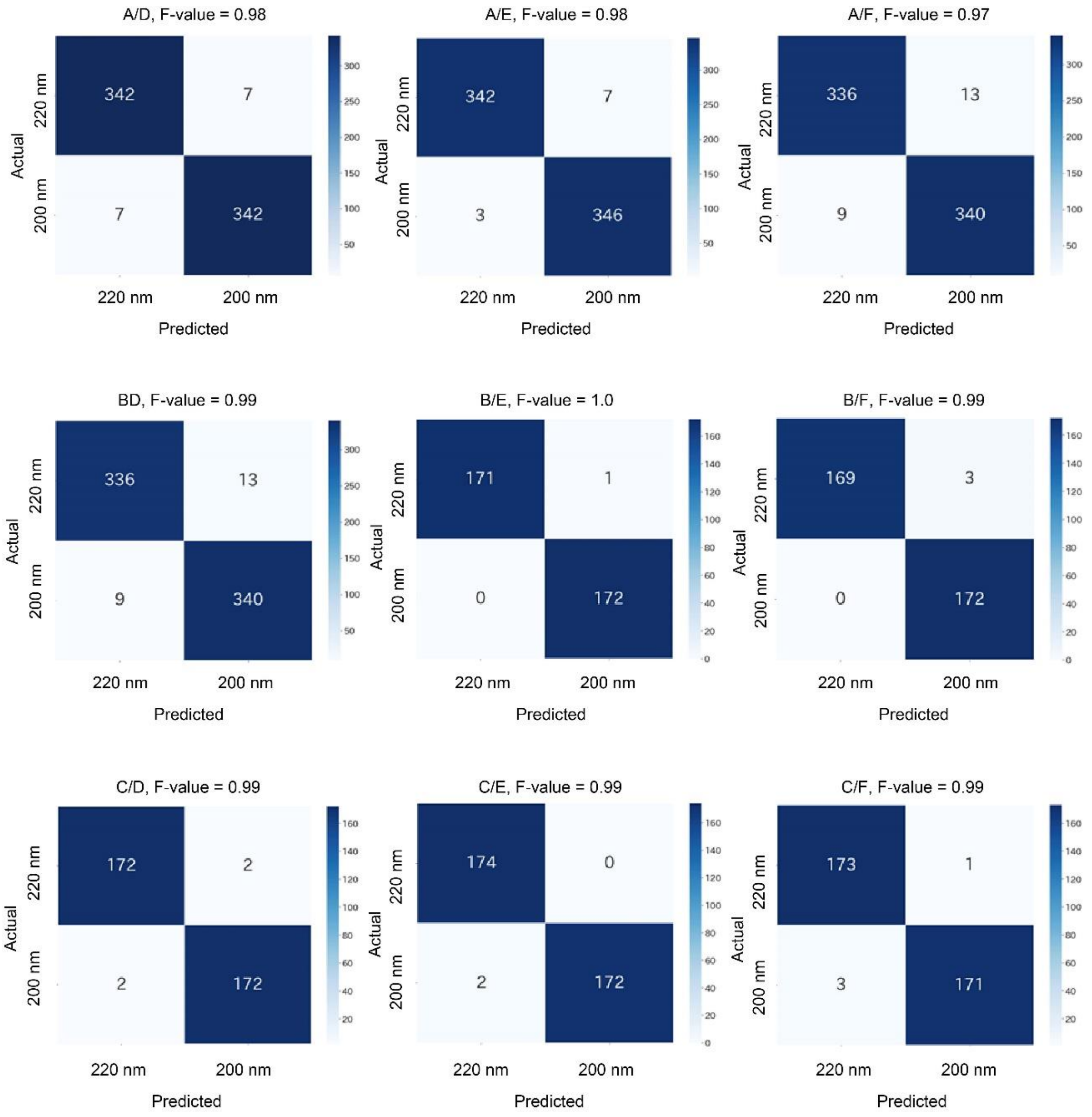

Figure S6 Confusion matrixes of nanoparticles with diameter of 200 nm and 220 nm. The numbers in the matrix elements indicate the number of waveforms obtained by the measurement. The darker the diagonal line, the higher is the identification accuracy. The F-value is calculated from the confusion matrices. The colour bar indicates that the darker the blue, the greater the number of pulses. The number of the colour bar indicated the number of waveforms.

Table S3 Number of waveforms obtained from nanoparticle measurements employing the same nanopores and number of waveforms as those used for machine learning.

Nanopore number	Diameter	Extracted waveforms	Waveforms used for AI training
1	200 nm	891	796
	220 nm	796	796
2	200 nm	68	687
	220 nm	1089	687
3	200 nm	596	595
	220 nm	595	595

Pulses extracted automatically using Aipore-ONE^{TM} extraction software.

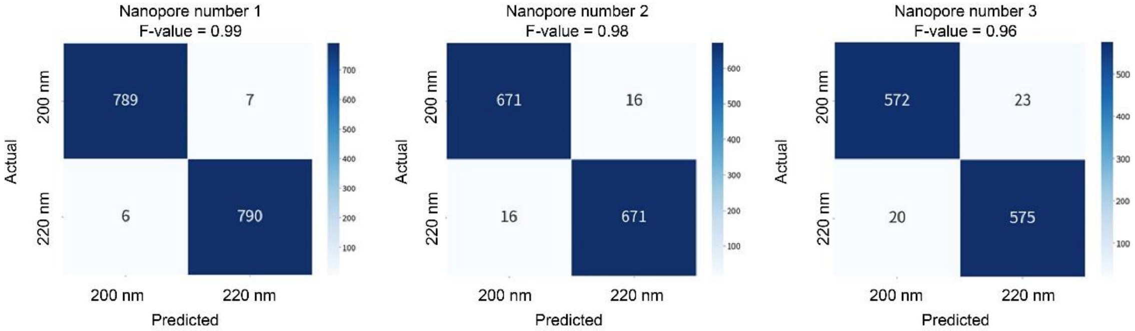

Figure S7 Confusion matrix and F-values during the measurement of 200 and 220 nm nanoparticles using the same nanopores. After measuring the 200 nm nanoparticles for 5 min, the nanopores were washed 3 times with a 1 × PBS buffer solution. Subsequently, the 220 nm nanoparticles were measured for 5 min. Three nanopores were used. The applied voltage was 0.1 V. High F-values (0.99, 0.98, and 0.97) indicate that they do not identify differences in the nanopores, but in 200 and 220 nm nanoparticles. Nanopore numbers correspond to that listed in Table S3. The colour bar indicates that the darker the blue, the greater the number of pulses. The number of the colour bar indicated the number of waveforms.

3. Identification of four cultured coronaviruses

Table S4 Number of waveforms obtained from the measurement of four coronaviruses and the number of waveforms used for machine learning

Virus	Number of nanopores for measurements	Extracted waveforms	Waveforms used for AI training
SARS-CoV	4	1051	640
MERS-CoV	9	645	640
SARS-CoV-2	6	765	640
HCoV-229E	5	2271	640

Pulses extracted automatically using Aipore-ONE^{TM} extraction software.

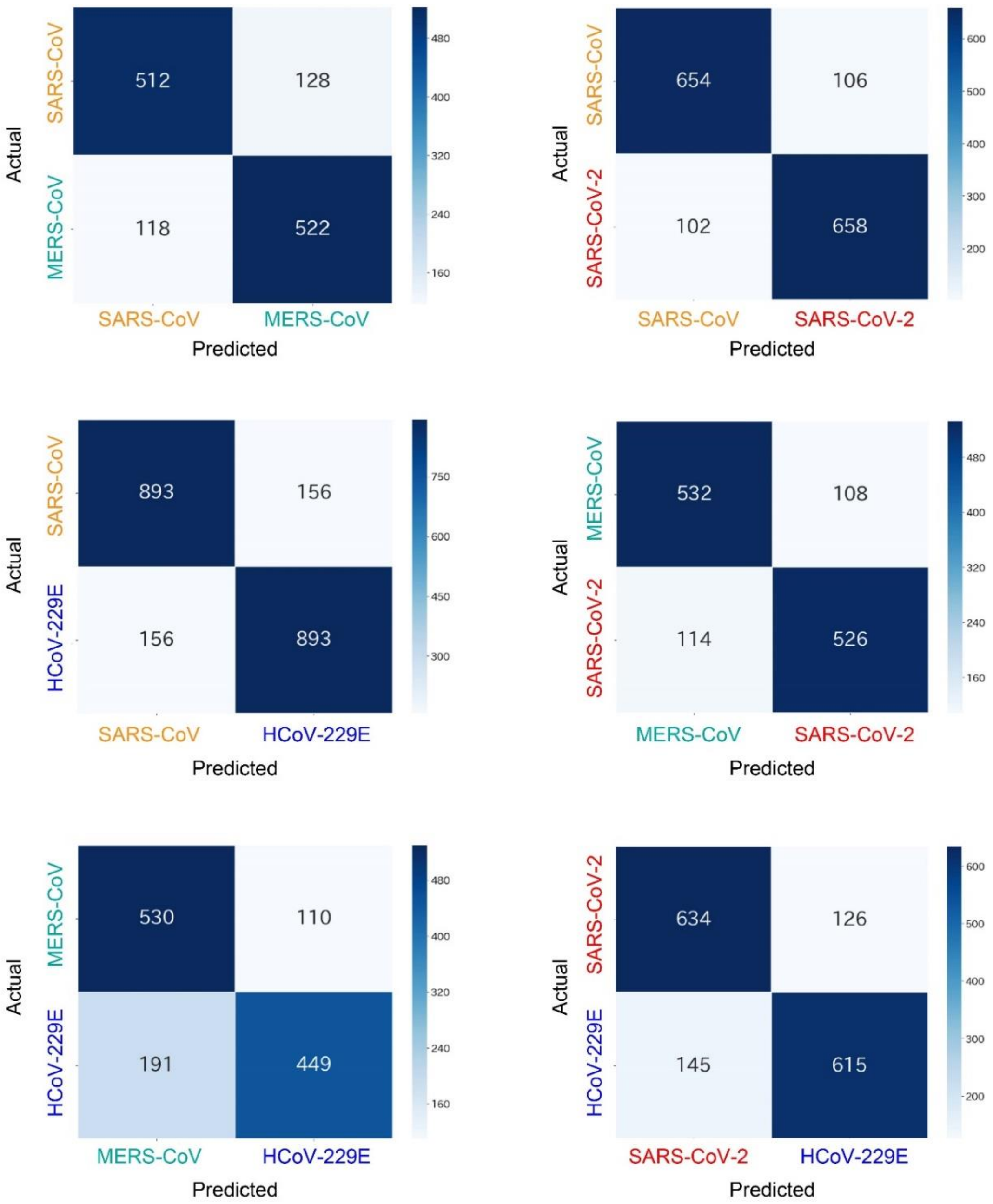

Figure S8 Confusion matrixes 2 types of cultured viruses. The numbers in the matrix elements indicate the number of waveforms obtained by measurement. The darker the diagonal line, the higher the identification accuracy. The F-value was calculated from these confusion matrices. The colour bar indicates that the darker the blue, the greater the number of pulses. The number of the colour bar indicated the number of waveforms.

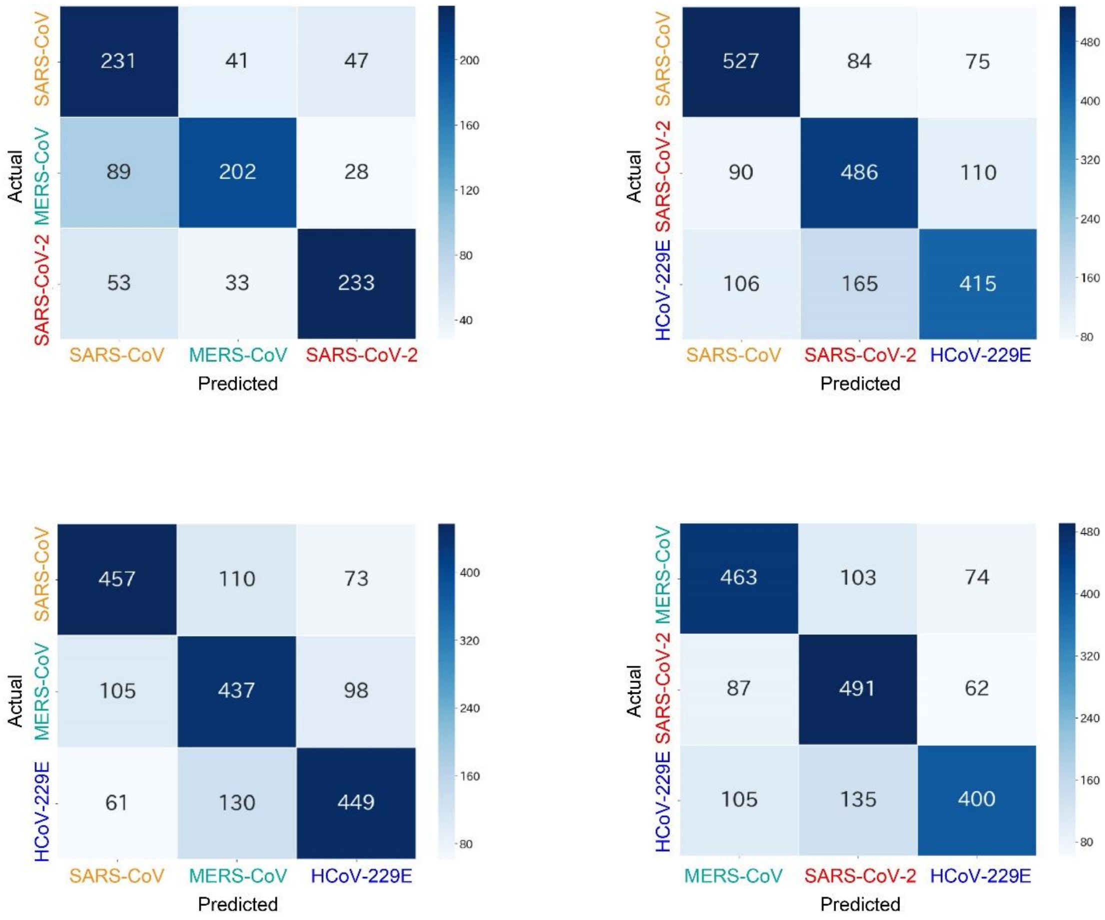

Figure S9 Confusion matrixes 3 types of cultured viruses. The numbers in the matrix elements indicate the number of waveforms obtained by measurement. The darker the diagonal line, the higher the identification accuracy. The F-value was calculated from these confusion matrices. The colour bar indicates that the darker the blue, the greater the number of pulses. The number of the colour bar indicated the number of waveforms.

4. Machine learning to identify clinical specimens

In clinical specimens, it is necessary to make a positive/negative judgment of the waveform and a positive/negative judgment of the specimens. For this reason, positive/negative judgments of waveforms and specimens were performed using an algorithm different from nanoparticles and cultured viruses (Figure 3d, 3e, S9, and S10). In machine learning of clinical specimens, it is assumed that the waveforms obtained from PCR-negative specimens are noise waveforms, and the waveforms obtained from PCR-positive specimens are the waveforms of the new coronavirus and noise waveforms. In this way, the PUC method is used in which noise is learned and the waveform of the virus is extracted from the waveform obtained from the PCR-positive specimen.

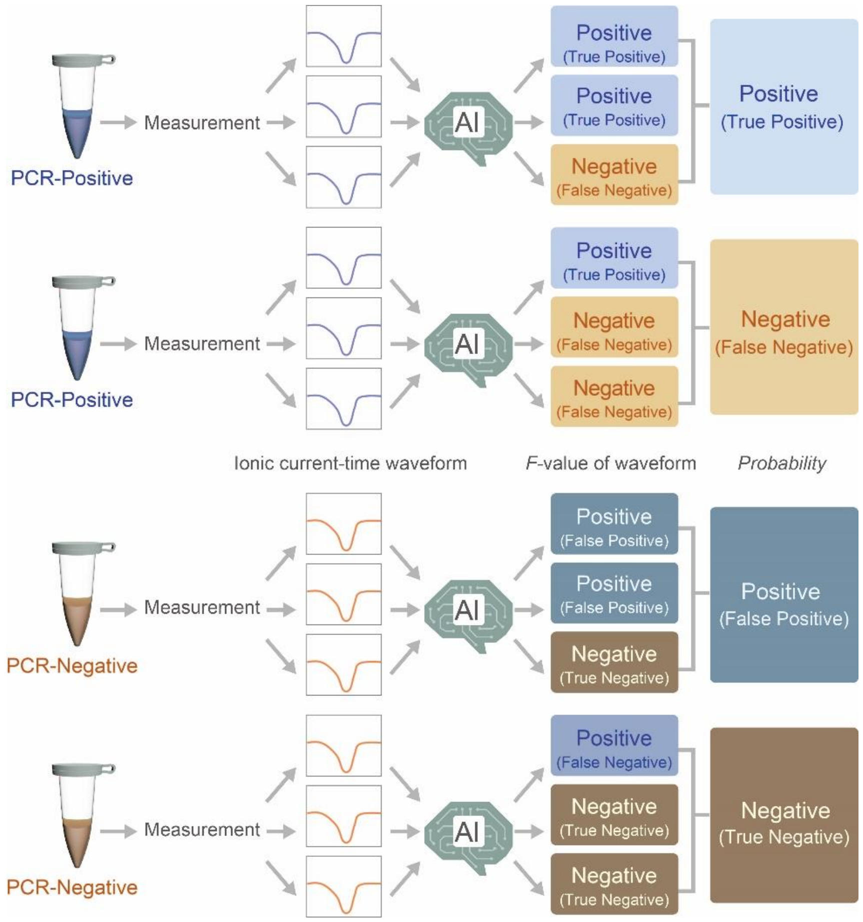

Figure S10. Schematic flowchart of machine learning of clinical specimens. After the PCR-positive and the PCR-negative samples are measured and ionic current-time waveforms are obtained (the waveforms are machine-learned), machine learning is used until the maximum accuracy for distinguishing between the positive and negative with a single waveform is obtained using the F-value as an index. Using equations (1), (2), and (4) in Figure S5, the F-value of one waveform is calculated. Since one sample gives a large number of waveforms, a large number of waveforms obtained from one sample are used to determine whether the sample is positive or negative. Machine learning is used until the highest probability that distinguishes between the positive and negative of one sample is obtained. Sensitivity and specificity are calculated using equations (2) and (3) in Figure S5.

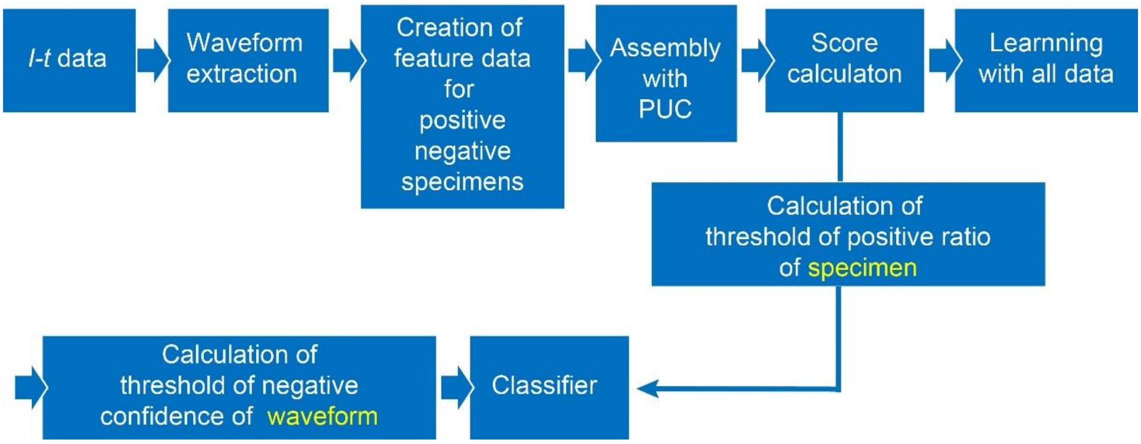

Figure S11 Detailed algorithm of learning process of clinical specimens. The positive ratio is the ratio of the number of positive waveforms in a specimen unit, and is defined by the ratio between the number of waveforms judged to be positive in one specimen to the total number of waveforms in specimen sample. A target specimen is classified as positive for the new coronavirus when the positive ratio exceeds a threshold value. The negative confidence threshold is the limiting value used for each waveform and on this basis the negative waveform is determined by the equation,

Negative waveform = negative confidence of waveform > negative confidence threshold

On the other hand, the positive waveform is determined by the equation,

Positive waveform = negative confidence of waveform < negative confidence threshold

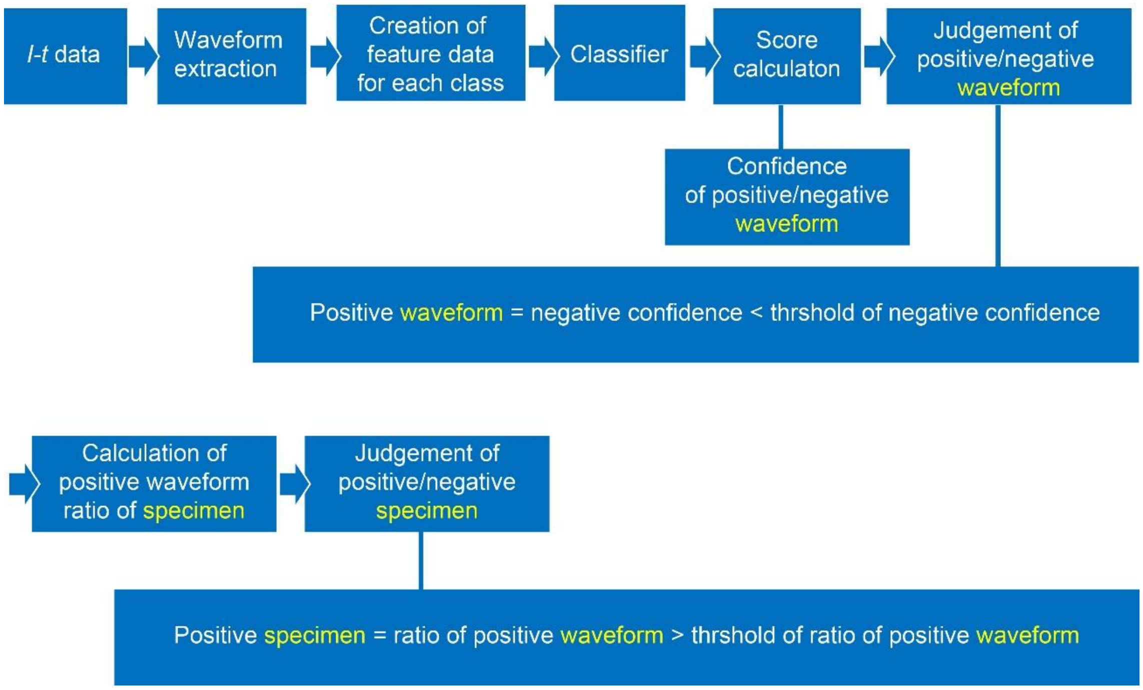

Figure S12 Detailed algorithm of diagnosis process of clinical specimens. The positive ratio is the ratio of the number of positive waveforms in a specimen unit, and is defined by (the number of waveforms judged to be positive in one specimen)/(the total number of waveforms in specimen sample). The threshold value of the positive ratio is a threshold value for determining the target specimen as positive when the positive ratio exceeds this value. The negative confidence threshold is the confidence threshold used for each waveform. For example, the negative waveform is determined by the following logic.

Negative waveform = negative confidence of waveform > negative confidence threshold

On the contrary, the positive waveform is determined by the following logic. Positive waveform = negative confidence of waveform < negative confidence threshold

5. Identification of SARS-Co-2 in clinical specimens of saliva

Table S5 Details on saliva samples used while implementing machine learning algorithm

Specimens	Number of nanopores for measurements	Extracted waveforms	Waveforms used for AI training
PCR-positive	48	23,973	13,687
PCR-negative	42	20,781	20,776

Pulses extracted automatically using Aipore-ONE^{TM} extraction software.

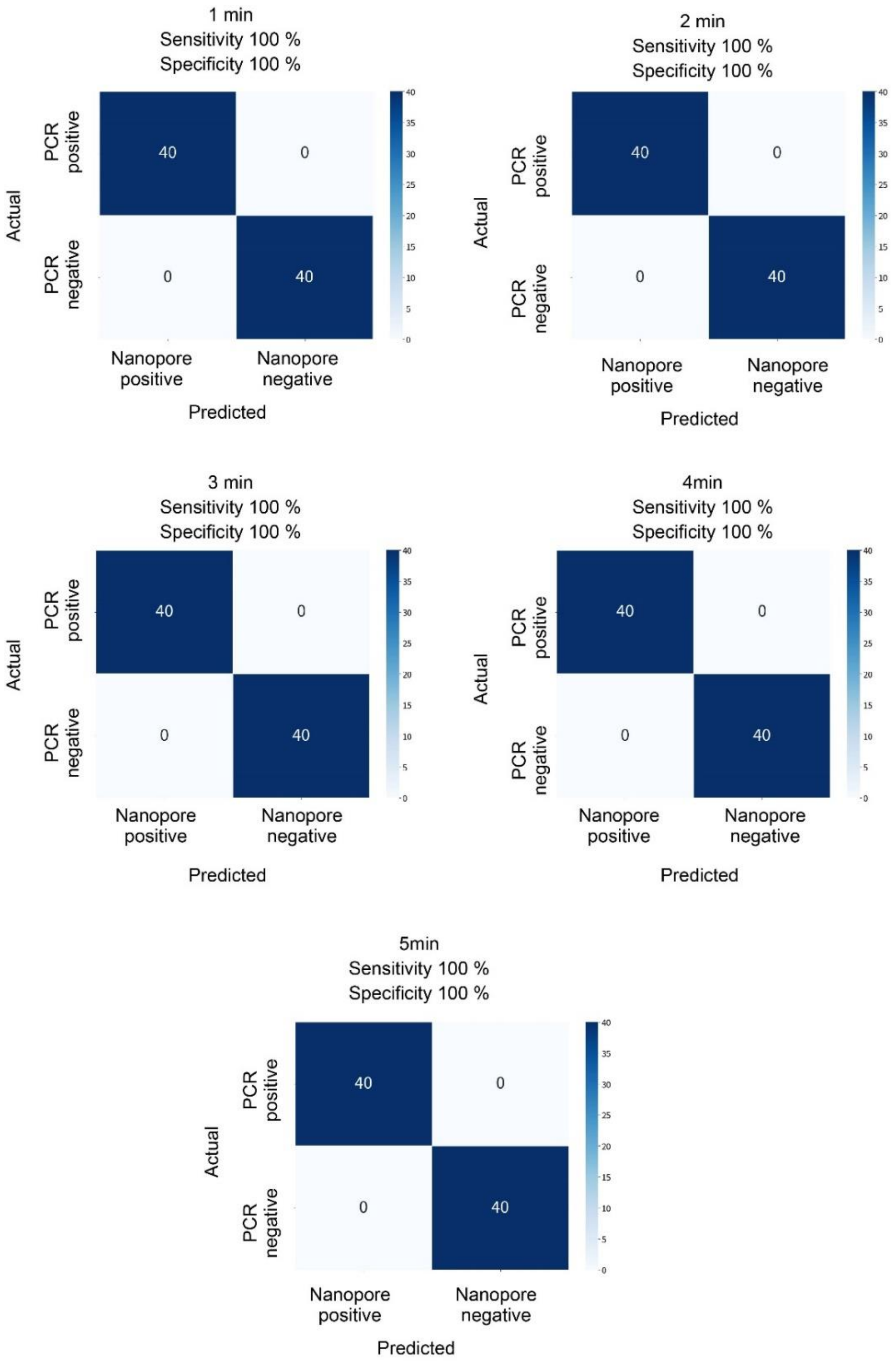

Figure S13 Time dependence of the confusion matrixes of saliva in the learning process. The numbers in the matrix cells indicate the number of specimens. The colour bar indicates that the darker the blue, the greater the number of pulses. The number of the colour bar indicated the number of specimens.

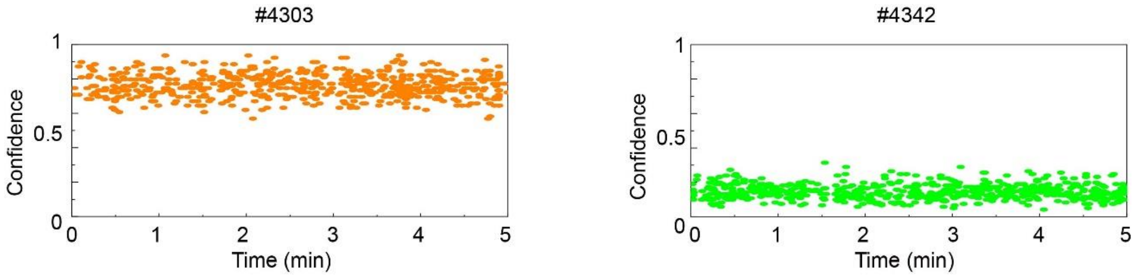

Figure S14 Waveform confidence in saliva specimens as a function of time in the learning process. Orange and green spots represent the waveforms judged to be positive and negative, respectively. #4304 and #4342 represent the sample numbers in Table S5.

Table S6 Details of the saliva samples used for the diagnostic process

Specimens	Number of nanopores for measurements	Extracted waveforms	Waveforms used for AI training
PCR-positive	58	28,560	12,172
PCR-negative	59	19,115	19,115

Pulses extracted automatically using Aipore-ONE^{TM} extraction software.

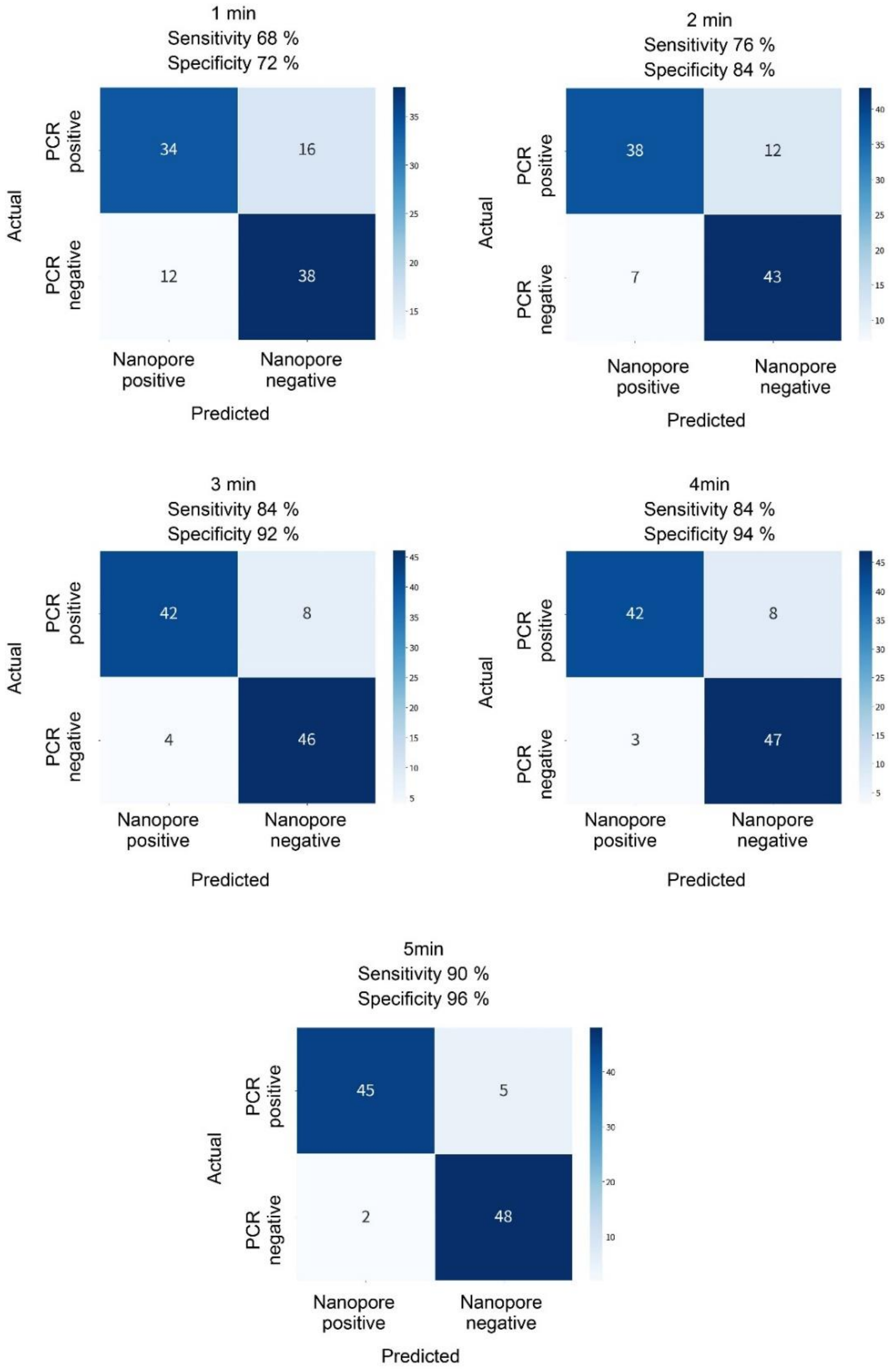

Figure S15 Time dependence of confusion matrixes of saliva in diagnostic process. The numbers in the matrix cells indicate the number of the specimens. The colour bar indicates that the darker the blue, the greater the number of pulses. The number of the colour bar indicated the number of specimens.

Distinguishing between cultured new coronavirus and cultured influenza virus.

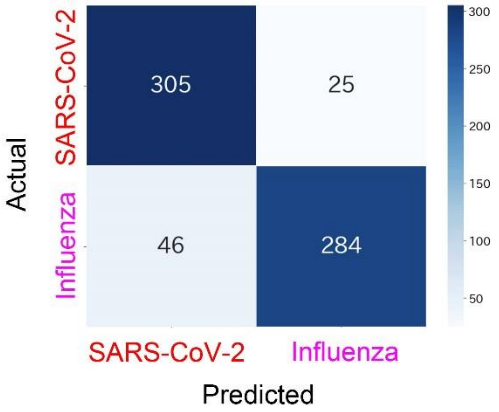

Figure S16 Confusion matrix of cultured SARS-CoV-2 and influenza A virus (N1H1). The numbers in the matrix indicate the number of waveforms obtained by measurement. The F-value was calculated from this confusion matrix. The colour bar indicates that the darker the blue, the greater the number of pulses. The number of the colour bar indicated the number of waveforms.

Table S7 PCR primers used in this study.

Primer name	Sequence (5'-3')
Primers for HCoV-229E	
229E-RdRp-F	TTGAAGGTGTCTCTGTTTGG
229E-RdRp-R	CTATGACACCTGAAGCAACT
229E-RdRp-probe	FAM-TGCTTTGTTGCTTCTTCCAC-TAMRA
229E-RdRp-sc-F	AAACCTGTGTTTATTAGTGC
229E-RdRp-sc-R	AGCAAAGTACGCCACATCGCCAAT
Primers for SARS-CoV-2	
2019-nCoV_N1-F	GACCCCAAAATCAGCGAAAT
2019-nCoV_N1-R	TCTGGTTACTGCCAGTTGAATCTG
2019-nCoV_N1-P	ACCCCGCATTACGTTTGGTGGACC
2019-nCoV_N2-F	TTACAAACATTGGCCGCAAA
2019-nCoV_N2-R	GCGCGACATTCCGAAGAA
2019-nCoV_N2-P	ACAATTTGCCCCCAGCGCTTCAG

6. Supplementary Reference

1. Holmes, G., Donkin, A., & Witten, I. H. *WEKA: a Machine Learning Workbench*, ANZIIS '94 - Australian New Zealnd Intelligent Information Systems Conference, Brisbane, Queensland, Australia, Nov 29-Dec 2, 1994: Institute of Electrical and Electronics Engineers: New York, 1994.
