# Supplementary material for: Combining machine learning and nanopore construction creates an artificial intelligence nanopore for coronavirus detection
Source: Nat Commun. 2021 Jun 17;12:3726. doi: 10.1038/s41467-021-24001-2 (PMC8211865; doi:10.1038/s41467-021-24001-2)
Supplement: Supplementary file 3 — Description of Additional Supplementary Files [file 41467_2021_24001_MOESM3_ESM.pdf]

Description of Additional Supplementary Files

File Name: Supplementary Data 1
Description: Errata for diagnosis obtained by machine learning following a 5 min measurement of saliva in the learning process.

File Name: Supplementary Data 2
Description: Errata for diagnosis obtained by machine learning following a 5 min measurement of saliva in the diagnostic process.
