# Supplementary material for: Combining machine learning and nanopore construction creates an artificial intelligence nanopore for coronavirus detection
Source: Nat Commun. 2021 Jun 17;12:3726. doi: 10.1038/s41467-021-24001-2 (PMC8211865; doi:10.1038/s41467-021-24001-2)
Supplement: Supplementary file 4 — Supplementary Data 1-2 [file 41467_2021_24001_MOESM4_ESM.zip › Supplementary-Data.docx]

**Supplementary Data for**

**Combining machine learning and nanopore construction creates an artificial intelligence nanopore for coronavirus detection**

Masateru Taniguchi, Shohei Minami, Chikako Ono, Rina Hamajima, Ayumi Morimura, Shigeto Hamaguchi, Yukihiro Akeda, Yuta Kanai, Takeshi Kobayashi, Wataru Kamitani, Yutaka Terada, Koichiro Suzuki, Nobuaki Hatori, Yoshiaki Yamagishi, Nobuei Washizu, Hiroyasu Takei, Osamu Sakamoto, Norihiko Naono, Kenji Tatematsu, Takashi Washio, Yoshiharu Matsuura & Kazunori Tomono

The Supplementary Data includes:

1. Errata for diagnosis obtained by machine learning following a 5 min measurement of saliva in the learning process (Data 1)
2. Errata for diagnosis obtained by machine learning following a 5 min measurement of saliva in the diagnostic process. (Data 2)

**Supplementary Data 1 Errata for diagnosis obtained by machine learning following a 5 min measurement of saliva in the learning process.**

| Specimen number | PCR results | Nanopore results | Positive confidence | Positive  ratio |
| --- | --- | --- | --- | --- |
| 4342 | Negative | Negative | 1.0000 | 0.0000 |
| 4341 | Negative | Negative | 1.0000 | 0.0000 |
| 4340 | Negative | Negative | 1.0000 | 0.0000 |
| 4339 | Negative | Negative | 1.0000 | 0.0000 |
| 4338 | Negative | Negative | 1.0000 | 0.0000 |
| 4337 | Negative | Negative | 1.0000 | 0.0000 |
| 4336 | Negative | Negative | 1.0000 | 0.0000 |
| 4335 | Negative | Negative | 1.0000 | 0.0000 |
| 4334 | Negative | Negative | 1.0000 | 0.0000 |
| 4333 | Negative | Negative | 1.0000 | 0.0000 |
| 4332 | Negative | Negative | 1.0000 | 0.0000 |
| 4331 | Negative | Negative | 1.0000 | 0.0000 |
| 4330 | Negative | Negative | 1.0000 | 0.0000 |
| 4329 | Negative | Negative | 1.0000 | 0.0000 |
| 4328 | Negative | Negative | 1.0000 | 0.0000 |
| 4327 | Negative | Negative | 1.0000 | 0.0000 |
| 4326 | Negative | Negative | 1.0000 | 0.0000 |
| 4325 | Negative | Negative | 1.0000 | 0.0000 |
| 4324 | Negative | Negative | 1.0000 | 0.0000 |
| 4323 | Negative | Negative | 1.0000 | 0.0000 |
| 4322 | Negative | Negative | 1.0000 | 0.0000 |
| 4321 | Negative | Negative | 1.0000 | 0.0000 |
| 4320 | Negative | Negative | 1.0000 | 0.0000 |
| 4319 | Negative | Negative | 1.0000 | 0.0000 |
| 4318 | Negative | Negative | 1.0000 | 0.0000 |
| 4317 | Negative | Negative | 1.0000 | 0.0000 |
| 4316 | Negative | Negative | 1.0000 | 0.0000 |
| 4315 | Negative | Negative | 1.0000 | 0.0000 |
| 4314 | Negative | Negative | 1.0000 | 0.0000 |
| 4313 | Negative | Negative | 1.0000 | 0.0000 |
| 4312 | Negative | Negative | 1.0000 | 0.0000 |
| 4311 | Negative | Negative | 1.0000 | 0.0000 |
| 4310 | Negative | Negative | 1.0000 | 0.0000 |
| 4309 | Negative | Negative | 1.0000 | 0.0000 |
| 4308 | Negative | Negative | 1.0000 | 0.0000 |
| 4307 | Negative | Negative | 1.0000 | 0.0000 |
| 4306 | Negative | Negative | 1.0000 | 0.0000 |
| 4305 | Negative | Negative | 1.0000 | 0.0000 |
| 4304 | Negative | Negative | 1.0000 | 0.0000 |
| 4303 | Positive | Positive | 1.0000 | 1.0000 |
| 4302 | Positive | Positive | 1.0000 | 1.0000 |
| 4301 | Positive | Positive | 1.0000 | 1.0000 |
| 4300 | Positive | Positive | 1.0000 | 1.0000 |
| 4299 | Positive | Positive | 1.0000 | 1.0000 |
| 4298 | Positive | Positive | 1.0000 | 1.0000 |
| 4297 | Positive | Positive | 1.0000 | 1.0000 |
| 4296 | Positive | Positive | 1.0000 | 1.0000 |
| 4295 | Positive | Positive | 1.0000 | 1.0000 |
| 4294 | Positive | Positive | 1.0000 | 1.0000 |
| 4293 | Positive | Positive | 1.0000 | 1.0000 |
| 4292 | Positive | Positive | 1.0000 | 1.0000 |
| 4291 | Positive | Positive | 1.0000 | 1.0000 |
| 4290 | Positive | Positive | 0.9985 | 0.9976 |
| 4289 | Positive | Positive | 1.0000 | 1.0000 |
| 4288 | Positive | Positive | 1.0000 | 1.0000 |
| 4287 | Positive | Positive | 1.0000 | 1.0000 |
| 4286 | Positive | Positive | 1.0000 | 1.0000 |
| 4285 | Positive | Positive | 1.0000 | 1.0000 |
| 4284 | Positive | Positive | 1.0000 | 1.0000 |
| 4283 | Positive | Positive | 1.0000 | 1.0000 |
| 4282 | Positive | Positive | 1.0000 | 1.0000 |
| 4281 | Positive | Positive | 1.0000 | 1.0000 |
| 4280 | Positive | Positive | 1.0000 | 1.0000 |
| 4279 | Positive | Positive | 1.0000 | 1.0000 |
| 4278 | Positive | Positive | 1.0000 | 1.0000 |
| 4277 | Positive | Positive | 1.0000 | 1.0000 |
| 4276 | Positive | Positive | 1.0000 | 1.0000 |
| 4275 | Positive | Positive | 1.0000 | 1.0000 |
| 4274 | Positive | Positive | 0.8807 | 0.8062 |
| 4273 | Positive | Positive | 1.0000 | 1.0000 |
| 4272 | Positive | Positive | 0.7418 | 0.5805 |
| 4271 | Positive | Positive | 1.0000 | 1.0000 |
| 4270 | Positive | Positive | 1.0000 | 1.0000 |
| 4269 | Positive | Positive | 1.0000 | 1.0000 |
| 4268 | Positive | Positive | 1.0000 | 1.0000 |
| 4267 | Positive | Positive | 1.0000 | 1.0000 |
| 4266 | Positive | Positive | 1.0000 | 1.0000 |
| 4265 | Positive | Positive | 0.8790 | 0.8033 |
| 4264 | Positive | Positive | 1.0000 | 1.0000 |
| 4343 | Negative | Negative | 1.0000 | 0.0000 |

**Supplementary Data 2 Errata for diagnosis obtained by machine learning following a 5 min measurement of saliva in the diagnostic process.**

| Specimen number | PCR results | Nanopore results | Positive confidence | Positive  ratio |
| --- | --- | --- | --- | --- |
| 4442 | Negative | Negative | 0.5327 | 0.1636 |
| 4441 | Negative | Negative | 0.8180 | 0.0637 |
| 4440 | Negative | Negative | 0.5803 | 0.1469 |
| 4439 | Negative | Negative | 0.5489 | 0.1579 |
| 4438 | Negative | Negative | 0.7581 | 0.0847 |
| 4437 | Negative | Negative | 0.6237 | 0.1317 |
| 4436 | Negative | Negative | 0.6167 | 0.1341 |
| 4435 | Negative | Negative | 0.7044 | 0.1034 |
| 4434 | Negative | Negative | 0.7143 | 0.1000 |
| 4433 | Negative | Negative | 0.6889 | 0.1089 |
| 4432 | Negative | Positive | 0.5152 | 0.2000 |
| 4431 | Negative | Negative | 0.8152 | 0.0647 |
| 4430 | Negative | Negative | 0.5604 | 0.1538 |
| 4429 | Negative | Negative | 0.6989 | 0.1054 |
| 4428 | Negative | Negative | 0.5155 | 0.1696 |
| 4427 | Negative | Negative | 0.6703 | 0.1154 |
| 4426 | Negative | Negative | 0.5122 | 0.1707 |
| 4425 | Negative | Negative | 0.6474 | 0.1234 |
| 4424 | Negative | Negative | 0.7283 | 0.0951 |
| 4423 | Negative | Negative | 0.6560 | 0.1204 |
| 4422 | Negative | Negative | 0.6728 | 0.1145 |
| 4421 | Negative | Negative | 0.7351 | 0.0927 |
| 4420 | Negative | Positive | 0.5202 | 0.2083 |
| 4419 | Negative | Negative | 0.7771 | 0.0780 |
| 4418 | Negative | Negative | 0.7819 | 0.0763 |
| 4417 | Negative | Negative | 0.7447 | 0.0893 |
| 4416 | Negative | Negative | 0.6032 | 0.1389 |
| 4415 | Negative | Negative | 0.5172 | 0.1690 |
| 4414 | Negative | Negative | 0.5031 | 0.1739 |
| 4413 | Negative | Negative | 0.7013 | 0.1046 |
| 4412 | Negative | Negative | 0.6304 | 0.1294 |
| 4411 | Negative | Negative | 0.6070 | 0.1376 |
| 4410 | Negative | Negative | 0.7027 | 0.1041 |
| 4409 | Negative | Negative | 0.7008 | 0.1047 |
| 4408 | Negative | Negative | 0.7894 | 0.0737 |
| 4407 | Negative | Negative | 0.7518 | 0.0869 |
| 4406 | Negative | Negative | 0.7577 | 0.0848 |
| 4405 | Negative | Negative | 0.5414 | 0.1605 |
| 4404 | Negative | Negative | 0.5390 | 0.1614 |
| 4403 | Negative | Negative | 0.7403 | 0.0909 |
| 4402 | Negative | Negative | 0.5367 | 0.1622 |
| 4401 | Negative | Negative | 0.6703 | 0.1154 |
| 4400 | Negative | Negative | 0.8260 | 0.0609 |
| 4399 | Negative | Negative | 0.6447 | 0.1244 |
| 4398 | Negative | Negative | 0.6438 | 0.1247 |
| 4397 | Negative | Negative | 0.7754 | 0.0786 |
| 4396 | Negative | Negative | 0.6073 | 0.1375 |
| 4395 | Negative | Negative | 0.6906 | 0.1083 |
| 4394 | Negative | Negative | 0.7584 | 0.0846 |
| 4393 | Positive | Positive | 0.5726 | 0.2949 |
| 4392 | Positive | Positive | 0.5338 | 0.2308 |
| 4391 | Positive | Negative | 0.5303 | 0.1644 |
| 4390 | Positive | Positive | 0.6633 | 0.4444 |
| 4389 | Positive | Positive | 0.5455 | 0.2500 |
| 4388 | Positive | Positive | 0.5709 | 0.2920 |
| 4387 | Positive | Positive | 0.5112 | 0.1935 |
| 4386 | Positive | Positive | 0.6459 | 0.4157 |
| 4385 | Positive | Negative | 0.6662 | 0.1168 |
| 4384 | Positive | Positive | 0.5286 | 0.2222 |
| 4383 | Positive | Positive | 0.5074 | 0.1872 |
| 4382 | Positive | Positive | 0.5257 | 0.2174 |
| 4381 | Positive | Positive | 0.5722 | 0.2941 |
| 4380 | Positive | Positive | 0.5671 | 0.2857 |
| 4379 | Positive | Positive | 0.5240 | 0.2146 |
| 4378 | Positive | Positive | 0.6979 | 0.5015 |
| 4377 | Positive | Positive | 0.5214 | 0.2103 |
| 4376 | Positive | Positive | 0.5365 | 0.2353 |
| 4375 | Positive | Positive | 0.7980 | 0.6667 |
| 4374 | Positive | Positive | 0.5144 | 0.1988 |
| 4373 | Positive | Positive | 0.6970 | 0.5000 |
| 4372 | Positive | Positive | 0.5098 | 0.1912 |
| 4371 | Positive | Positive | 0.5960 | 0.3333 |
| 4370 | Positive | Positive | 0.5506 | 0.2585 |
| 4369 | Positive | Positive | 0.5152 | 0.2000 |
| 4368 | Positive | Positive | 0.5553 | 0.2662 |
| 4367 | Positive | Positive | 0.5096 | 0.1909 |
| 4366 | Positive | Positive | 0.5066 | 0.1858 |
| 4365 | Positive | Positive | 0.5134 | 0.1971 |
| 4364 | Positive | Positive | 0.5004 | 0.1757 |
| 4363 | Positive | Negative | 0.6581 | 0.1197 |
| 4362 | Positive | Positive | 0.5469 | 0.2524 |
| 4361 | Positive | Positive | 0.5380 | 0.2376 |
| 4360 | Positive | Negative | 0.6160 | 0.1344 |
| 4359 | Positive | Positive | 0.5576 | 0.2700 |
| 4358 | Positive | Positive | 0.6674 | 0.4512 |
| 4357 | Positive | Positive | 0.6230 | 0.3780 |
| 4356 | Positive | Positive | 0.5045 | 0.1824 |
| 4355 | Positive | Positive | 0.6378 | 0.4023 |
| 4354 | Positive | Positive | 0.5494 | 0.2565 |
| 4353 | Positive | Positive | 0.5296 | 0.2239 |
| 4352 | Positive | Positive | 0.7152 | 0.5300 |
| 4351 | Positive | Positive | 0.6143 | 0.3636 |
| 4350 | Positive | Positive | 0.6248 | 0.3810 |
| 4349 | Positive | Positive | 0.5317 | 0.2273 |
| 4348 | Positive | Positive | 0.5286 | 0.2222 |
| 4347 | Positive | Positive | 0.5257 | 0.2174 |
| 4346 | Positive | Positive | 0.5349 | 0.2325 |
| 4345 | Positive | Negative | 0.5879 | 0.1442 |
| 4344 | Positive | Positive | 0.5147 | 0.1992 |
| 4443 | Negative | Negative | 0.6765 | 0.1132 |

The blue and yellow markers indicate false negatives and false positives, respectively.
