# Supplementary material for: Combining machine learning and nanopore construction creates an artificial intelligence nanopore for coronavirus detection
Source: Nat Commun. 2021 Jun 17;12:3726. doi: 10.1038/s41467-021-24001-2 (PMC8211865; doi:10.1038/s41467-021-24001-2)
Supplement: Supplementary file 5 — Reporting-Summary [file 41467_2021_24001_MOESM5_ESM.pdf]

nature research

Corresponding author(s): Masateru Taniguchi, Yoshiharu Matsuura, Kazunori Tomono

Last updated by author(s): May 27, 2021

Reporting Summary

Nature Research wishes to improve the reproducibility of the work that we publish. This form provides structure for consistency and transparency in reporting. For further information on Nature Research policies, see our Editorial Policies and the Editorial Policy Checklist.

Statistics

For all statistical analyses, confirm that the following items are present in the figure legend, table legend, main text, or Methods section.

n/a	Confirmed	
☐	☒	The exact sample size (n) for each experimental group/condition, given as a discrete number and unit of measurement
☐	☒	A statement on whether measurements were taken from distinct samples or whether the same sample was measured repeatedly
☒	☐	The statistical test(s) used AND whether they are one- or two-sided<br>*Only common tests should be described solely by name; describe more complex techniques in the Methods section.*
☒	☐	A description of all covariates tested
☐	☒	A description of any assumptions or corrections, such as tests of normality and adjustment for multiple comparisons
☐	☒	A full description of the statistical parameters including central tendency (e.g. means) or other basic estimates (e.g. regression coefficient) AND variation (e.g. standard deviation) or associated estimates of uncertainty (e.g. confidence intervals)
☒	☐	For null hypothesis testing, the test statistic (e.g. F, t, r) with confidence intervals, effect sizes, degrees of freedom and P value noted<br>*Give P values as exact values whenever suitable.*
☒	☐	For Bayesian analysis, information on the choice of priors and Markov chain Monte Carlo settings
☐	☒	For hierarchical and complex designs, identification of the appropriate level for tests and full reporting of outcomes
☐	☒	Estimates of effect sizes (e.g. Cohen's d, Pearson's r), indicating how they were calculated

Our web collection on statistics for biologists contains articles on many of the points above.

Software and code

Policy information about availability of computer code

Data collection	No software was used.
Data analysis	All of the machine learning was done with Aipore-OneTM, commercial software from Aipore Inc. We include a README file in Zenodo (https://doi.org/10.5281/zenodo. 4761714) that enables researchers to log in to the Aipore server through a web browser to train the AI model on Aipore-OneTM.

For manuscripts utilizing custom algorithms or software that are central to the research but not yet described in published literature, software must be made available to editors and reviewers. We strongly encourage code deposition in a community repository (e.g. GitHub). See the Nature Research guidelines for submitting code & software for further information.

Data

Policy information about availability of data

All manuscripts must include a data availability statement. This statement should provide the following information, where applicable:

- Accession codes, unique identifiers, or web links for publicly available datasets
- A list of figures that have associated raw data
- A description of any restrictions on data availability

Source data are provided with this paper.
Figures 1f-1j, 1l,2a, 2b, 2d-2g, 3a-3c, 3f-3k, Supplementary Figures S6-S9,S13-S16, Supplementary Tables S1-S6.
The measurement data of the current-time profile of the nanoparticles, four types of viruses, and clinical specimens obtained in this study are available on Zenodo (https://doi.org/10.5281/zenodo.4761714).

Field-specific reporting

Please select the one below that is the best fit for your research. If you are not sure, read the appropriate sections before making your selection.

☒ Life sciences ☐ Behavioural & social sciences ☐ Ecological, evolutionary & environmental sciences

For a reference copy of the document with all sections, see nature.com/documents/nr-reporting-summary-flat.pdf

Life sciences study design

All studies must disclose on these points even when the disclosure is negative.

Sample size	40 PCR negative specimens and 40 PCR positive specimens were used for the learning process. 50 PCR negative specimens and 50 PCR positive specimens were used for the diagnostic process. The sample size was determined by the maximum available clinical and laboratory information in Osaka University Hospital. No prior sample size calculations was performed.
Data exclusions	No data were excluded from the analysis.
Replication	Specimens were acquired as residual samples from clinical examination with the approval of Institutional Review Board, Osaka University Hospital, Osaka University, Japan. The need to obtain informed consent was waived by the IRB committee since the samples were residual from a clinical examination without using any identifiable information of the individuals or the application of any intervention, in accordance with the Ethical Guidelines for Medical and Health Research Involving Human Subjects. (Public Notice of the Ministry of Education, Culture, Sports, Science and Technology and the Ministry of Health, Labor and Welfare No. 3 of 2014). No experimental replication was performed for specimen measurements.
Randomization	A 10-fold cross validation using the current vector was conducted.
Blinding	During the learning process, the investigators were not blinded because investigators cannot do machine learning without knowing whether specimen is PCR positive or PCR negative. During the diagnostic process, the investigators were blinded.

Reporting for specific materials, systems and methods

We require information from authors about some types of materials, experimental systems and methods used in many studies. Here, indicate whether each material, system or method listed is relevant to your study. If you are not sure if a list item applies to your research, read the appropriate section before selecting a response.

Materials & experimental systems

n/a	Involved in the study	
☒	☐	Antibodies
☐	☒	Eukaryotic cell lines
☒	☐	Palaeontology and archaeology
☒	☐	Animals and other organisms
☐	☒	Human research participants
☒	☐	Clinical data
☒	☐	Dual use research of concern

Methods

n/a	Involved in the study	
☒	☐	ChIP-seq
☒	☐	Flow cytometry
☒	☐	MRI-based neuroimaging

Eukaryotic cell lines

Policy information about cell lines

Cell line source(s)	1. HeLa cells from ATCC<br>2. African green monkey kidney Vero cells. We purchased Vero cell line from ATCC® CCL-81™.<br>3. MDCK cells from ECACC 84121903
Authentication	1. No Authentication<br>2. We used STR profiling to authenticate Vero cell line.<br>3. No Authentication
Mycoplasma contamination	1. HeLa cells were not tested for mycoplasma contamination<br>2. The cell line was tested negative.<br>3. The cell line was tested negative.
Commonly misidentified lines<br>(See ICLAC register)	We declare that no commonly misidentified cell lines were used in the study.

Human research participants

Policy information about studies involving human research participants

Population characteristics	*Describe the covariate-relevant population characteristics of the human research participants (e.g. age, gender, genotypic information, past and current diagnosis and treatment categories). If you filled out the behavioural & social sciences study design questions and have nothing to add here, write "See above."*
Recruitment	*Describe how participants were recruited. Outline any potential self-selection bias or other biases that may be present and how these are likely to impact results.*
Ethics oversight	*Identify the organization(s) that approved the study protocol.*

Note that full information on the approval of the study protocol must also be provided in the manuscript.
