# Supplementary material for: Combining machine learning and nanopore construction creates an artificial intelligence nanopore for coronavirus detection
Source: Nat Commun. 2021 Jun 17;12:3726. doi: 10.1038/s41467-021-24001-2 (PMC8211865; doi:10.1038/s41467-021-24001-2)
Supplement: Supplementary file 6 — Source Data [file 41467_2021_24001_MOESM6_ESM.zip › Source-Data.docx]

**Source Data for**

**Combining machine learning and nanopore construction creates an artificial intelligence nanopore for coronavirus detection**

Masateru Taniguchi, Shohei Minami, Chikako Ono, Rina Hamajima, Ayumi Morimura, Shigeto Hamaguchi, Yukihiro Akeda, Yuta Kanai, Takeshi Kobayashi, Wataru Kamitani, Yutaka Terada, Koichiro Suzuki, Nobuaki Hatori, Yoshiaki Yamagishi, Nobuei Washizu, Hiroyasu Takei, Osamu Sakamoto, Norihiko Naono, Kenji Tatematsu, Takashi Washio, Yoshiharu Matsuura & Kazunori Tomono

The measurement data of the current-time profile of the nanoparticles, four types of viruses, and clinical specimens obtained in this study are available on Zenodo ([https://doi.org/10.5281/zenodo. 4761714](https://doi.org/10.5281/zenodo.4750951)). The table indicates correspondence between data in the database and samples and figures in the main text, Supplementary Information, and Supplementary Data.

| Zip name | Folder name | File name | Sample | Figure(s) |
| --- | --- | --- | --- | --- |
| Nanoparticles_I-t_data-20210427T153558Z-001.zip | 20201118_A101_Thermo200nm_x100_1 | 20201118_A101_Thermo200nm_x100_1_001.dat  20201118_A101_Thermo200nm_x100_1_002dat  20201118_A101_Thermo200nm_x100_1_003.dat | 200 nm-nanoparticles | Figs. 1f-1l, S6  Tables S1. S2 |
|  | 20201118_A101_Thermo200nm_x100_2 | 20201118_A101_Thermo200nm_x100_2_001.dat  20201118_A101_Thermo200nm_x100_2_002.dat  20201118_A101_Thermo200nm_x100_2_003.dat | 200 nm-nanoparticles | Figs. 1f-1l, S6  Tables S1. S2 |
|  | 20201118_A101_Thermo200nm_x100_3 | 20201118_A101_Thermo200nm_x100_3_001.dat  20201118_A101_Thermo200nm_x100_3_002.dat  20201118_A101_Thermo200nm_x100_3_003.dat | 200 nm-nanoparticles | Figs. 1f-1l, S6  Tables S1. S2 |
|  | 20201118_A101_Thermo220nm_x100_2 | 20201118_A101_Thermo220nm_x100_2_001.dat  20201118_A101_Thermo220nm_x100_2_002.dat  20201118_A101_Thermo220nm_x100_2_003.dat | 220 nm-nanoparticles | Figs. 1f-1l, S6  Tables S1. S2 |
|  | 20201118_A101_Thermo220nm_x100_3 | 20201118_A101_Thermo220nm_x100_3_001.dat  20201118_A101_Thermo220nm_x100_3_001.dat  20201118_A101_Thermo220nm_x100_3_001.dat | 220 nm-nanoparticles | Figs. 1f-1l, S6  Tables S1. S2 |
|  | 20201118_A101_Thermo220nm_x100_4 | 20201118_A101_Thermo220nm_x100_4_001.dat  20201118_A101_Thermo220nm_x100_4_002.dat  20201118_A101_Thermo220nm_x100_4_003.dat | 220 nm-nanoparticles | Figs. 1f-1l, S6  Tables S1. S2 |
| Nanoparticles_I-t data-20210209T115422Z-001.zip | A_200nm_1st | A_200nm_1st_002.dat  A_200nm_1st_004.dat  A_200nm_1st_005.dat | 200 nm-nanoparticles | Fig. S7  Table S3 |
|  | A_200nm_2st | A_200nm_2st_002.dat  A_200nm_2st_003.dat  A_200nm_2st_004.dat  A_200nm_2st_005.dat | 200 nm nanoparticles | Fig. S7  Table S3 |
|  | A_200nm_3rd | A_200nm_3rd_001.dat  A_200nm_3rd_002.dat  A_200nm_3rd_003.dat  A_200nm_3rd_004.dat  A_200nm_3rd_005.dat | 200 nm nanoparticles | Fig. S7  Table S3 |
|  | A_220nm_1st | A_220nm_1st_003.dat  A_220nm_1st_005.dat | 220 nm-nanoparticles | Fig. S7  Table S3 |
|  | A_220nm_2st | A_220nm_2st_004.dat  A_220nm_2st_005.dat | 220 nm nanoparticles | Fig. S7  Table S3 |
|  | A_220nm_3rd | A_220nm_3rd_001.dat  A_220nm_3rd_004.dat  A_220nm_3rd_005.dat | 220 nm nanoparticles | Fig. S7  Table S3 |
| Nanoparticles_I-t data-20210209T115422Z-002.zip | A_200nm_1st | A_200nm_1st_001.dat  A_200nm_1st_003dat | 200 nm-nanoparticles | Fig. S7  Table S3 |
|  | A_200nm_2st | A_200nm_2st_001.dat | 200 nm nanoparticles | Fig. S7  Table S3 |
|  | A_220nm_1st | A_220nm_1st_001.dat  A_220nm_1st_002.dat  A_220nm_1st_004.dat | 220 nm-nanoparticles | Fig. S7  Table S3 |
|  | A_220nm_2st | A_220nm_2st_001.dat  A_220nm_2st_002.dat  A_220nm_2st_003.dat | 220 nm nanoparticles | Fig. S7  Table S3 |
|  | A_220nm_3rd | A_220nm_3rd_002.dat  A_220nm_3rd_003.dat | 220 nm nanoparticles | Fig. S7  Table S3 |
| Cultured corona virus_I-t data-20210209T120053Z-001.zip | HCoV-229E/20200622154659 | NK-2-1-1st-FIL_004.dat  NK-2-1-1st-FIL_006.dat | Cultured HCoV-229E | Figs. 2a, 2b, 2d-2g, S8, S9  Tables S4 |
|  | HCoV-229E/20200622161115 | NK-2-1-3rd-FIL_004.dat  NK-2-1-3rd-FIL_005.dat  NK-2-1-3rd-FIL_006.dat  NK-2-1-3rd-FIL_007.dat  NK-2-1-3rd-FIL_008.dat  NK-2-1-3rd-FIL_009.dat  NK-2-1-3rd-FIL_010.dat  NK-2-1-3rd-FIL_013.dat | Cultured HCoV-229E | Figs. 2a, 2b, 2d-2g, S8, S9  Tables S4 |
|  | HCoV-229E/20200622164240 | NK-2-1-4th-FIL_001.dat  NK-2-1-4th-FIL_002.dat | Cultured HCoV-229E | Figs. 2a, 2b, 2d-2g, S8, S9  Tables S4 |
|  | MERS-CoV/20200708140539 | NK-2-1-MERSx2-3rd_003.dat | Cultured MERS-CoV | Figs. 2a, 2b, 2d-2g, S8, S9  Tables S4 |
|  | MERS-CoV/20200708142922 | NK-2-1-MERSx2-6th_001.dat | Cultured MERS-CoV | Figs. 2a, 2b, 2d-2g, S8, S9  Tables S4 |
|  | MERS-CoV/20200708144806 | NK-2-1-MERSx2-9th_005.dat | Cultured MERS-CoV | Figs. 2a, 2b, 2d-2g, S8, S9  Tables S4 |
|  | MERS-CoV/20200708145355 | NK-2-1-MERSx2-9th_003.dat | Cultured MERS-CoV | Figs. 2a, 2b, 2d-2g, S8, S9  Tables S4 |
|  | SARS-CoV/20200707143029 | NK-2-1-SARSx10-2nd_001.dat | Cultured SARS-CoV | Figs. 2a, 2b, 2d-2g, S8, S9  Tables S4 |
|  | SARS-CoV/20200707145745 | NK-2-1-SARSx10-4th_008.dat | Cultured SARS-CoV | Figs. 2a, 2b, 2d-2g, S8, S9  Tables S4 |
|  | SARS-CoV-2/20200709164824 | NK-2-1-SARS2x2-5th_004.dat | Cultured SARS-CoV-2 | Figs. 2a, 2b, 2d-2g, S8, S9  Tables S4 |
|  | SARS-CoV-2/20200709170243 | NK-2-1-SARS2x2-5th_007.dat | Cultured SARS-CoV-2 | Figs. 2a, 2b, 2d-2g, S8, S9  Tables S4 |
| Cultured corona virus_I-t data-20210209T120053Z-002.zip | HCoV-229E/20200622154659 | NK-2-1-1st-FIL_001.dat  NK-2-1-1st-FIL_002.dat  NK-2-1-1st-FIL_003.dat  NK-2-1-1st-FIL_005.dat | Cultured HCoV-229E | Figs. 2a, 2b, 2d-2g, S8, S9  Tables S4 |
|  | HCoV-229E/20200622161115 | NK-2-1-3rd-FIL_001.dat  NK-2-1-3rd-FIL_002.dat NK-2-1-3rd-FIL_003.dat  NK-2-1-3rd-FIL_011.dat | Cultured HCoV-229E | Figs. 2a, 2b, 2d-2g, S8, S9  Tables S4 |
|  | MERS-CoV/20200708140539 | NK-2-1-MERSx2-3rd_001.dat  NK-2-1-MERSx2-3rd_002.dat | Cultured MERS-CoV | Figs. 2a, 2b, 2d-2g, S8, S9  Tables S4 |
|  | MERS-CoV/20200708140846 | NK-2-1-MERSx2-3rd_001.dat | Cultured MERS-CoV | Figs. 2a, 2b, 2d-2g, S8, S9  Tables S4 |
|  | MERS-CoV/20200708142549 | NK-2-1-MERSx2-6th_003.dat | Cultured MERS-CoV | Figs. 2a, 2b, 2d-2g, S8, S9  Tables S4 |
|  | MERS-CoV/20200708144046 | NK-2-1-MERSx2-8th_005.dat | Cultured MERS-CoV | Figs. 2a, 2b, 2d-2g, S8, S9  Tables S4 |
|  | MERS-CoV/20200708145355 | NK-2-1-MERSx2-9th_001.dat | Cultured MERS-CoV | Figs. 2a, 2b, 2d-2g, S8, S9  Tables S4 |
|  | SARS-CoV-2/20200709164824 | NK-2-1-SARS2x2-5th_002.dat  NK-2-1-SARS2x2-5th_003.dat | Cultured SARS-CoV-2 | Figs. 2a, 2b, 2d-2g, S8, S9  Tables S4 |
| Cultured corona virus_I-t data-20210209T120053Z-003.zip | MERS-CoV/20200708144046 | NK-2-1-MERSx2-8th_001.dat  NK-2-1-MERSx2-8th_002.dat  NK-2-1-MERSx2-8th_003.dat  NK-2-1-MERSx2-8th_004.dat | Cultured MERS-CoV | Figs. 2a, 2b, 2d-2g, S8, S9  Tables S4 |
|  | MERS-CoV/20200708144806 | NK-2-1-MERSx2-9th_001.dat  NK-2-1-MERSx2-9th_002.dat  NK-2-1-MERSx2-9th_003.dat  NK-2-1-MERSx2-9th_004.dat | Cultured MERS-CoV | Figs. 2a, 2b, 2d-2g, S8, S9  Tables S4 |
|  | MERS-CoV/20200708145355 | NK-2-1-MERSx2-9th_002.dat | Cultured MERS-CoV | Figs. 2a, 2b, 2d-2g, S8, S9  Tables S4 |
|  | SARS-CoV/20200707141746 | NK-2-1-SARSx10-1st_002.dat  NK-2-1-SARSx10-1st_004.dat | Cultured SARS-CoV | Figs. 2a, 2b, 2d-2g, S8, S9  Tables S4 |
|  | SARS-CoV/20200707142324 | NK-2-1-SARSx10-1st_002.dat | Cultured SARS-CoV | Figs. 2a, 2b, 2d-2g, S8, S9  Tables S4 |
|  | SARS-CoV/20200707143029 | NK-2-1-SARSx10-2nd_009.dat | Cultured SARS-CoV | Figs. 2a, 2b, 2d-2g, S8, S9  Tables S4 |
|  | SARS-CoV/20200707144609 | NK-2-1-SARSx10-3rd_007.dat | Cultured SARS-CoV | Figs. 2a, 2b, 2d-2g, S8, S9  Tables S4 |
|  | SARS-CoV-2/ 20200709164824 | NK-2-1-SARS2x2-5th_001.dat | Cultured SARS-CoV-2 | Figs. 2a, 2b, 2d-2g, S8, S9  Tables S4 |
|  | SARS-CoV-2/20200709170243 | NK-2-1-SARS2x2-5th_004.dat  NK-2-1-SARS2x2-5th_005.dat  NK-2-1-SARS2x2-5th_006.dat | Cultured SARS-CoV-2 | Figs. 2a, 2b, 2d-2g, S8, S9  Tables S4 |
|  | SARS-CoV-2/20200709171240 | NK-2-1-SARS2x2-5th_005.dat | Cultured SARS-CoV-2 | Figs. 2a, 2b, 2d-2g, S8, S9  Tables S4 |
| Cultured corona virus_I-t data-20210209T120053Z-004.zip | HCoV-229E/20200622161115 | NK-2-1-3rd-FIL_012.dat | Cultured HCoV-229E | Figs. 2a, 2b, 2d-2g, S8, S9  Tables S4 |
|  | HCoV-229E/20200721161425 | NK-2-1-bias-01-229E-1st_001.dat | Cultured HCoV-229E | Figs. 2a, 2b, 2d-2g, S8, S9  Tables S4 |
|  | SARS-CoV/20200707141746 | NK-2-1-SARSx10-1st_001.dat  NK-2-1-SARSx10-1st_003.dat | Cultured SARS-CoV | Figs. 2a, 2b, 2d-2g, S8, S9  Tables S4 |
|  | SARS-CoV/20200707142324 | NK-2-1-SARSx10-1st_001.dat | Cultured SARS-CoV | Figs. 2a, 2b, 2d-2g, S8, S9  Tables S4 |
|  | SARS-CoV/20200707144609 | NK-2-1-SARSx10-3rd_001.dat  NK-2-1-SARSx10-3rd_002.dat  NK-2-1-SARSx10-3rd_003.dat  NK-2-1-SARSx10-3rd_004.dat  NK-2-1-SARSx10-3rd_005.dat  NK-2-1-SARSx10-3rd_006.dat | Cultured SARS-CoV | Figs. 2a, 2b, 2d-2g, S8, S9  Tables S4 |
|  | SARS-CoV-2/20200709170243 | NK-2-1-SARS2x2-5th_001.dat  NK-2-1-SARS2x2-5th_002.dat  NK-2-1-SARS2x2-5th_003.dat | Cultured SARS-CoV-2 | Figs. 2a, 2b, 2d-2g, S8, S9  Tables S4 |
| Cultured corona virus_I-t data-20210209T120053Z-005.zip | HCoV-229E/20200721144423 | NK-2-1-bias-01-229E-1st_002.dat | Cultured HCoV-229E | Figs. 2a, 2b, 2d-2g, S8, S9  Tables S4 |
|  | HCoV-229E/20200721150255 | NK-2-1-bias-01-229E-1st_002.dat | Cultured HCoV-229E | Figs. 2a, 2b, 2d-2g, S8, S9  Tables S4 |
|  | MERS-CoV/20200708142549 | NK-2-1-MERSx2-6th_002.dat | Cultured MERS-CoV | Figs. 2a, 2b, 2d-2g, S8, S9  Tables S4 |
|  | SARS-CoV/20200707145745 | NK-2-1-SARSx10-4th_001.dat  NK-2-1-SARSx10-4th_002.dat  NK-2-1-SARSx10-4th_004.dat  NK-2-1-SARSx10-4th_007.dat | Cultured SARS-CoV | Figs. 2a, 2b, 2d-2g, S8, S9  Tables S4 |
| Cultured corona virus_I-t data-20210209T120053Z-007.zip | HCoV-229E/20200721150255 | NK-2-1-bias-01-229E-1st_001.dat | Cultured HCoV-229E | Figs. 2a, 2b, 2d-2g, S8, S9  Tables S4 |
|  | MERS-CoV/20200708135350 | NK-2-1-MERSx2-2nd_005.dat  NK-2-1-MERSx2-2nd_006.dat  NK-2-1-MERSx2-2nd_008.dat | Cultured MERS-CoV | Figs. 2a, 2b, 2d-2g, S8, S9  Tables S4 |
|  | MERS-CoV/20200708142549 | NK-2-1-MERSx2-6th_001.dat | Cultured MERS-CoV | Figs. 2a, 2b, 2d-2g, S8, S9  Tables S4 |
|  | SARS-CoV/20200707143029 | NK-2-1-SARSx10-2nd_005.dat  NK-2-1-SARSx10-2nd_006.dat | Cultured SARS-CoV | Figs. 2a, 2b, 2d-2g, S8, S9  Tables S4 |
|  | SARS-CoV/20200707145745 | NK-2-1-SARSx10-4th_006.dat | Cultured SARS-CoV | Figs. 2a, 2b, 2d-2g, S8, S9  Tables S4 |
| Cultured corona virus_I-t data-20210209T120053Z-008.zip | HCoV-229E/20200721140708 | NK-2-1-bias-01-229E-1st_002.dat | Cultured HCoV-229E | Figs. 2a, 2b, 2d-2g, S8, S9  Tables S4 |
|  | MERS-CoV/20200708135350 | NK-2-1-MERSx2-2nd_001.dat  NK-2-1-MERSx2-2nd_002.dat  NK-2-1-MERSx2-2nd_003.dat  NK-2-1-MERSx2-2nd_004.dat  NK-2-1-MERSx2-2nd_007.dat | Cultured MERS-CoV | Figs. 2a, 2b, 2d-2g, S8, S9  Tables S4 |
|  | SARS-CoV/20200707143029 | NK-2-1-SARSx10-2nd_002.dat  NK-2-1-SARSx10-2nd_007.dat | Cultured SARS-CoV | Figs. 2a, 2b, 2d-2g, S8, S9  Tables S4 |
|  | SARS-CoV-2/20200709171240 | NK-2-1-SARS2x2-5th_001.dat  NK-2-1-SARS2x2-5th_002.dat  NK-2-1-SARS2x2-5th_003.dat  NK-2-1-SARS2x2-5th_004dat | Cultured SARS-CoV-2 | Figs. 2a, 2b, 2d-2g, S8, S9  Tables S4 |
| Cultured corona virus_I-t data-20210209T120053Z-009.zip | HCoV-229E/20200721134852 | NK-2-1-bias-01-229E-1st_002.dat | Cultured HCoV-229E | Figs. 2a, 2b, 2d-2g, S8, S9  Tables S4 |
|  | HCoV-229E/20200721151936 | NK-2-1-bias-01-229E-1st_002.dat | Cultured HCoV-229E | Figs. 2a, 2b, 2d-2g, S8, S9  Tables S4 |
|  | SARS-CoV/20200707143029 | NK-2-1-SARSx10-2nd_003.dat  NK-2-1-SARSx10-2nd_004.dat  NK-2-1-SARSx10-2nd_008.dat | Cultured SARS-CoV | Figs. 2a, 2b, 2d-2g, S8, S9  Tables S4 |
| Cultured corona virus_I-t data-20210209T120053Z-010.zip | HCoV-229E/20200721140708 | NK-2-1-bias-01-229E-1st_001.dat | Cultured HCoV-229E | Figs. 2a, 2b, 2d-2g, S8, S9  Tables S4 |
|  | HCoV-229E/20200721142400 | NK-2-1-bias-01-229E-1st_002.dat | Cultured HCoV-229E | Figs. 2a, 2b, 2d-2g, S8, S9  Tables S4 |
| Cultured corona virus_I-t data-20210209T120053Z-011.zip | HCoV-229E/20200721133140 | NK-2-1-bias-01-229E-1st_002.dat | Cultured HCoV-229E | Figs. 2a, 2b, 2d-2g, S8, S9  Tables S4 |
|  | HCoV-229E/20200721151936 | NK-2-1-bias-01-229E-1st_001.dat | Cultured HCoV-229E | Figs. 2a, 2b, 2d-2g, S8, S9  Tables S4 |
| Cultured corona virus_I-t data-20210209T120053Z-012.zip | HCoV-229E/20200721155354 | NK-2-1-bias-01-229E-1st_001.dat  NK-2-1-bias-01-229E-1st_002.dat | Cultured HCoV-229E | Figs. 2a, 2b, 2d-2g, S8, S9  Tables S4 |
| Cultured corona virus_I-t data-20210209T120053Z-013.zip | HCoV-229E/20200721142400 | NK-2-1-bias-01-229E-1st_001.dat | Cultured HCoV-229E | Figs. 2a, 2b, 2d-2g, S8, S9  Tables S4 |
| Cultured corona virus_I-t data-20210209T120053Z-014.zip | HCoV-229E/20200721133140 | NK-2-1-bias-01-229E-1st_001.dat | Cultured HCoV-229E | Figs. 2a, 2b, 2d-2g, S8, S9  Tables S4 |
| Cultured corona virus_I-t data-20210209T120053Z-015.zip | HCoV-229E/20200721134852 | NK-2-1-bias-01-229E-1st_001.dat | Cultured HCoV-229E | Figs. 2a, 2b, 2d-2g, S8, S9  Tables S4 |
| Cultured corona virus_I-t data-20210209T120053Z-016.zip | HCoV-229E/ 20200721144423 | NK-2-1-bias-01-229E-1st_001.dat1.1 | Cultured HCoV-229E | Figs. 2a, 2b, 2d-2g, S8, S9  Tables S4 |
| Saliva(n=100 HO)_I-t data-20210209T093219Z-001.zip | test/Negative/HD-112720-20 | HD-112720-20_005.dat | PCR negative saliva for test | Figs. 3g, 3j, 3k S15, Table S6, Data S2 |
|  | test/Negative/HD-113020-23 | HD-113020-23_005.dat | PCR negative saliva for test | Figs. 3g, 3j, 3k S15, Table S6, Data S2 |
|  | test/Negative/HD-120220-57 | HD-120220-57_003.dat | PCR negative saliva for test | Figs. 3g, 3j, 3k S15, Table S6, Data S2 |
|  | test/Negative/HD-120420-3 | HD-120420-3_005.dat | PCR negative saliva for test | Figs. 3g, 3j, 3k S15, Table S6, Data S2 |
|  | test/Negative/HD-120720-11 | HD-120720-11_004.dat | PCR negative saliva for test | Figs. 3g, 3j, 3k S15, Table S6, Data S2 |
|  | test/Negative/HD-120720-25 | HD-120720-25_003.dat  HD-120720-25_005.dat | PCR negative saliva for test | Figs. 3g, 3j, 3k S15, Table S6, Data S2 |
|  | test/Negative/HD-120720-28 | HD-120720-28_004.dat | PCR negative saliva for test | Figs. 3g, 3j, 3k S15, Table S6, Data S2 |
|  | test/Negative/HD-120720-35 | HD-120720-35_001.dat  HD-120720-35_002.dat  HD-120720-35_003.dat  HD-120720-35_005.dat | PCR negative saliva for test | Figs. 3g, 3j, 3k S15, Table S6, Data S2 |
|  | test/Negative/HD-120720-45 | HD-120720-45_001.dat  HD-120720-45_002.dat  HD-120720-45_004.dat  HD-120720-45_005.dat | PCR negative saliva for test | Figs. 3g, 3j, 3k S15, Table S6, Data S2 |
|  | test/Positive/F10 day3 | F10 day3_003.dat | PCR positive saliva for test | Figs. 3g, 3j, 3k S15, Table S6, Data S2 |
|  | test/Positive/ Test/Positive/F9 day1 | F9 day1_007.dat | PCR positive saliva for test | Figs. 3g, 3j, 3k S15, Table S6, Data S2 |
|  | training/Negative/HD-112720-58 | HD-112720-58_005.dat | PCR negative saliva for training | Figs. 3a-3c, 3f, 3h, 3i, S13, S14, Table S5, Data S1 |
|  | Training/Negative/HD-113020-28 | HD-113020-28_002.dat | PCR negative saliva for training | Figs. 3a-3c, 3f, 3h, 3i, S13, S14, Table S5, Data S1 |
|  | training/Negative/HD-113020-3 | HD-113020-3_005.dat | PCR negative saliva for training | Figs. 3a-3c, 3f, 3h, 3i, S13, S14, Table S5, Data S1 |
|  | training/Negative/HD-120420-23 | HD-120420-23_002.dat  HD-120420-23_005.dat | PCR negative saliva for training | Figs. 3a-3c, 3f, 3h, 3i, S13, S14, Table S5, Data S1 |
|  | training/Negative/HD-120420-32 | HD-120420-32_005.dat | PCR negative saliva for training | Figs. 3a-3c, 3f, 3h, 3i, S13, S14, Table S5, Data S1 |
|  | training/Negative/HD-120420-41 | HD-120420-41_001.dat  HD-120420-41_003.dat  HD-120420-41_004.dat  HD-120420-41_005.dat | PCR negative saliva for training | Figs. 3a-3c, 3f, 3h, 3i, S13, S14, Table S5, Data S1 |
|  | training/Negative/HD-120720-12 | HD-120720-12_005.dat | PCR negative saliva for training | Figs. 3a-3c, 3f, 3h, 3i, S13, S14, Table S5, Data S1 |
|  | training/Negative/HD-120720-21 | HD-120720-21_004.dat  HD-120720-21_005.dat | PCR negative saliva for training | Figs. 3a-3c, 3f, 3h, 3i, S13, S14, Table S5, Data S1 |
|  | training/Negative/HD-120720-6 | HD-120720-6_005.dat | PCR negative saliva for training | Figs. 3a-3c, 3f, 3h, 3i, S13, S14, Table S5, Data S1 |
|  | training/Positive/AS-2-2-bias+01_BK-973_045fil_TI_1st | AS-2-2-bias+01_BK-973_045fil_TI_1st_007.dat | PCR positive saliva for training | Figs. 3a-3c, 3f, 3h, 3i, S13, S14, Table S5, Data S1 |
|  | training/Positive/F2 day7 | F2 day7_009.dat | PCR positive saliva for training | Figs. 3a-3c, 3f, 3h, 3i, S13, S14, Table S5, Data S1 |
|  | training/Positive/F6 day5 | F6 day5_001.dat | PCR positive saliva for training | Figs. 3a-3c, 3f, 3h, 3i, S13, S14, Table S5, Data S1 |
| Saliva(n=100 HO)_I-t data-20210209T093219Z-002.zip | test/Negative/HD-112720-25 | HD-112720-25_005.dat | PCR negative saliva for test | Figs. 3g, 3j, 3k S15, Table S6, Data S2 |
|  | test/Negative/HD-113020-17 | HD-113020-17_004.dat | PCR negative saliva for test | Figs. 3g, 3j, 3k S15, Table S6, Data S2 |
|  | test/Negative/HD-113020-45 | HD-113020-45_005.dat | PCR negative saliva for test | Figs. 3g, 3j, 3k S15, Table S6, Data S2 |
|  | test/Negative/HD-120720-11 | HD-120720-11_005.dat | PCR negative saliva for test | Figs. 3g, 3j, 3k S15, Table S6, Data S2 |
|  | test/Negative/HD-120720-25 | HD-120720-25_001.dat  HD-120720-25_002.dat  HD-120720-25_004.dat | PCR negative saliva for test | Figs. 3g, 3j, 3k S15, Table S6, Data S2 |
|  | test/Negative/HD-120720-35 | HD-120720-35_004.dat | PCR negative saliva for test | Figs. 3g, 3j, 3k S15, Table S6, Data S2 |
|  | test/Negative/HD-120720-45 | HD-120720-45_003.dat | PCR negative saliva for test | Figs. 3g, 3j, 3k S15, Table S6, Data S2 |
|  | test/Positive/AS-2-2-bias+01_BK-950_045fil_TI_1st | AS-2-2-bias+01_BK-950_045fil_TI_1st_003.dat | PCR positive saliva for test | Figs. 3g, 3j, 3k S15, Table S6, Data S2 |
|  | test/Positive/F3 day3 | F3 day3_004.dat | PCR positive saliva for test | Figs. 3g, 3j, 3k S15, Table S6, Data S2 |
|  | training/Negative/HD-113020-1 | HD-113020-1_005.dat | PCR negative saliva for training | Figs. 3a-3c, 3f, 3h, 3i, S13, S14, Table S5, Data S1 |
|  | training/Negative/HD-113020-28 | HD-113020-28_005.dat | PCR negative saliva for training | Figs. 3a-3c, 3f, 3h, 3i, S13, S14, Table S5, Data S1 |
|  | training/Negative/HD-113020-5 | HD-113020-5_001.dat | PCR negative saliva for training | Figs. 3a-3c, 3f, 3h, 3i, S13, S14, Table S5, Data S1 |
|  | training/Negative/HD-120220-19 | HD-120220-19_005.dat | PCR negative saliva for training | Figs. 3a-3c, 3f, 3h, 3i, S13, S14, Table S5, Data S1 |
|  | training/Negative/HD-120220-28 | HD-120220-28_005.dat | PCR negative saliva for training | Figs. 3a-3c, 3f, 3h, 3i, S13, S14, Table S5, Data S1 |
|  | training/Negative/HD-120220-47 | HD-120220-47_005.dat | PCR negative saliva for training | Figs. 3a-3c, 3f, 3h, 3i, S13, S14, Table S5, Data S1 |
|  | training/Negative/HD-120420-23 | HD-120420-23_001.dat  HD-120420-23_003.dat  HD-120420-23_004.dat | PCR negative saliva for training | Figs. 3a-3c, 3f, 3h, 3i, S13, S14, Table S5, Data S1 |
|  | training/Negative/HD-120420-41 | HD-120420-41_002.dat | PCR negative saliva for training | Figs. 3a-3c, 3f, 3h, 3i, S13, S14, Table S5, Data S1 |
|  | training/Negative/HD-120420-7 | HD-120420-7_005.dat | PCR negative saliva for training | Figs. 3a-3c, 3f, 3h, 3i, S13, S14, Table S5, Data S1 |
|  | training/Negative/HD-120720-21 | HD-120720-21_001.dat  HD-120720-21_002.dat  HD-120720-21_003.dat | PCR negative saliva for training | Figs. 3a-3c, 3f, 3h, 3i, S13, S14, Table S5, Data S1 |
|  | training/Negative/HD-120720-6 | HD-120720-6_003.dat  HD-120720-6_004.dat | PCR negative saliva for training | Figs. 3a-3c, 3f, 3h, 3i, S13, S14, Table S5, Data S1 |
|  | training/Positive/AS-2-2-bias+01_BK-1099_045fil_TI | AS-2-2-bias+01_BK-1099_045fil_TI_001.dat | PCR positive saliva for training | Figs. 3a-3c, 3f, 3h, 3i, S13, S14, Table S5, Data S1 |
|  | training/Positive/AS-2-2-bias+01_BK-1125_045fil_TI | AS-2-2-bias+01_BK-1125_045fil_TI_004.dat | PCR positive saliva for training | Figs. 3a-3c, 3f, 3h, 3i, S13, S14, Table S5, Data S1 |
|  | training/Positive/F11 day3 | F11 day3_008.dat | PCR positive saliva for training | Figs. 3a-3c, 3f, 3h, 3i, S13, S14, Table S5, Data S1 |
|  | training/Positive/F2 | F2_001.dat | PCR positive saliva for training | Figs. 3a-3c, 3f, 3h, 3i, S13, S14, Table S5, Data S1 |
|  | training/Positive/F4 day7 | F4 day7_009.dat | PCR positive saliva for training | Figs. 3a-3c, 3f, 3h, 3i, S13, S14, Table S5, Data S1 |
| Saliva(n=100 HO)_I-t data-20210209T093219Z-003.zip | test/Negative/HD-113020-47 | HD-113020-47_002.dat | PCR negative saliva for test | Figs. 3g, 3j, 3k S15, Table S6, Data S2 |
|  | test/Positive/AS-2-2-bias+01_BK-965_045fil_TI_1st | AS-2-2-bias+01_BK-965_045fil_TI_1st_008.dat | PCR positive saliva for test | Figs. 3g, 3j, 3k S15, Table S6, Data S2 |
|  | test/Positive/F1 | F1_005.dat | PCR positive saliva for test | Figs. 3g, 3j, 3k S15, Table S6, Data S2 |
|  | test/Positive/F7 day14 | F7 day14_001.dat  F7 day14_009.dat | PCR positive saliva for test | Figs. 3g, 3j, 3k S15, Table S6, Data S2 |
|  | training/Negative/HD-112720-18 | HD-112720-18_004.dat | PCR negative saliva for training | Figs. 3a-3c, 3f, 3h, 3i, S13, S14, Table S5, Data S1 |
|  | training/Negative/HD-112720-29 | HD-112720-29_005.dat | PCR negative saliva for training | Figs. 3a-3c, 3f, 3h, 3i, S13, S14, Table S5, Data S1 |
|  | training/Negative/HD-112720-58 | HD-112720-58_001.dat | PCR negative saliva for training | Figs. 3a-3c, 3f, 3h, 3i, S13, S14, Table S5, Data S1 |
|  | training/Negative/HD-120220-47 | HD-120220-47_002.dat  HD-120220-47_003.dat  HD-120220-47_004.dat | PCR negative saliva for training | Figs. 3a-3c, 3f, 3h, 3i, S13, S14, Table S5, Data S1 |
|  | training/Negative/HD-120420-17 | HD-120420-17_001.dat  HD-120420-17_005.dat | PCR negative saliva for training | Figs. 3a-3c, 3f, 3h, 3i, S13, S14, Table S5, Data S1 |
|  | training/Negative/HD-120420-32 | HD-120420-32_001.dat  HD-120420-32_002.dat  HD-120420-32_003.dat  HD-120420-32_004.dat | PCR negative saliva for training | Figs. 3a-3c, 3f, 3h, 3i, S13, S14, Table S5, Data S1 |
|  | training/Negative/HD-120420-7 | HD-120420-7_001.dat | PCR negative saliva for training | Figs. 3a-3c, 3f, 3h, 3i, S13, S14, Table S5, Data S1 |
|  | training/Negative/HD-120720-46 | HD-120720-46_005.dat | PCR negative saliva for training | Figs. 3a-3c, 3f, 3h, 3i, S13, S14, Table S5, Data S1 |
|  | training/Negative/HD-120720-6 | HD-120720-6_001.dat  HD-120720-6_002.dat | PCR negative saliva for training | Figs. 3a-3c, 3f, 3h, 3i, S13, S14, Table S5, Data S1 |
|  | training/Positive/AS-2-2-bias+01_BK-1094_045fil_TI | AS-2-2-bias+01_BK-1094_045fil_TI_008.dat | PCR positive saliva for training | Figs. 3a-3c, 3f, 3h, 3i, S13, S14, Table S5, Data S1 |
|  | training/Positive/AS-2-2-bias+01_BK-989_045fil_TI_1st | AS-2-2-bias+01_BK-989_045fil_TI_1st_003.dat | PCR positive saliva for training | Figs. 3a-3c, 3f, 3h, 3i, S13, S14, Table S5, Data S1 |
|  | training/Positive/F16 day1 | F16 day1_001.dat  F16 day1_002.dat  F16 day1_003.dat  F16 day1_006.dat  F16 day1_009.dat | PCR positive saliva for training | Figs. 3a-3c, 3f, 3h, 3i, S13, S14, Table S5, Data S1 |
| Saliva(n=100 HO)_I-t data-20210209T093219Z-004.zip | test/Negative/HD-112720-39 | HD-112720-39_005.dat | PCR negative saliva for test | Figs. 3g, 3j, 3k S15, Table S6, Data S2 |
|  | test/Negative/HD-112720-45 | HD-112720-45_003.dat | PCR negative saliva for test | Figs. 3g, 3j, 3k S15, Table S6, Data S2 |
|  | test/Negative/HD-120220-57 | HD-120220-57_002.dat | PCR negative saliva for test | Figs. 3g, 3j, 3k S15, Table S6, Data S2 |
|  | test/Negative/HD-120420-18 | HD-120420-18_002.dat | PCR negative saliva for test | Figs. 3g, 3j, 3k S15, Table S6, Data S2 |
|  | test/Negative/HD-120720-28 | HD-120720-28_005.dat | PCR negative saliva for test | Figs. 3g, 3j, 3k S15, Table S6, Data S2 |
|  | test/Positive/F10 day1① | F10 day1①_008.dat | PCR positive saliva for test | Figs. 3g, 3j, 3k S15, Table S6, Data S2 |
|  | test/Positive/F13 day3 | F13 day3_007.dat | PCR positive saliva for test | Figs. 3g, 3j, 3k S15, Table S6, Data S2 |
|  | test/Positive/F7 day14 | F7 day14_005.dat | PCR positive saliva for test | Figs. 3g, 3j, 3k S15, Table S6, Data S2 |
|  | training/Negative/HD-112720-21 | HD-112720-21_005.dat | PCR negative saliva for training | Figs. 3a-3c, 3f, 3h, 3i, S13, S14, Table S5, Data S1 |
|  | training/Negative/HD-112720-44 | HD-112720-44_005.dat | PCR negative saliva for training | Figs. 3a-3c, 3f, 3h, 3i, S13, S14, Table S5, Data S1 |
|  | training/Negative/HD-113020-43 | HD-113020-43_005.dat | PCR negative saliva for training | Figs. 3a-3c, 3f, 3h, 3i, S13, S14, Table S5, Data S1 |
|  | training/Negative/HD-113020-5 | HD-113020-5_005.dat | PCR negative saliva for training | Figs. 3a-3c, 3f, 3h, 3i, S13, S14, Table S5, Data S1 |
|  | training/Negative/HD-113020-7 | HD-113020-7_002.dat | PCR negative saliva for training | Figs. 3a-3c, 3f, 3h, 3i, S13, S14, Table S5, Data S1 |
|  | training/Negative/HD-120220-21 | HD-120220-21_005.dat | PCR negative saliva for training | Figs. 3a-3c, 3f, 3h, 3i, S13, S14, Table S5, Data S1 |
|  | training/Negative/HD-120220-47 | HD-120220-47_001.dat | PCR negative saliva for training | Figs. 3a-3c, 3f, 3h, 3i, S13, S14, Table S5, Data S1 |
|  | training/Negative/HD-120420-17 | HD-120420-17_002.dat  HD-120420-17_003.dat  HD-120420-17_004.dat | PCR negative saliva for training | Figs. 3a-3c, 3f, 3h, 3i, S13, S14, Table S5, Data S1 |
|  | training/Negative/HD-120420-7 | HD-120420-7_002.dat  HD-120420-7_003.dat  HD-120420-7_004.dat | PCR negative saliva for training | Figs. 3a-3c, 3f, 3h, 3i, S13, S14, Table S5, Data S1 |
|  | training/Negative/HD-120720-12 | HD-120720-12_003.dat  HD-120720-12_004.dat | PCR negative saliva for training | Figs. 3a-3c, 3f, 3h, 3i, S13, S14, Table S5, Data S1 |
|  | training/Negative/HD-120720-17 | HD-120720-17_005.dat | PCR negative saliva for training | Figs. 3a-3c, 3f, 3h, 3i, S13, S14, Table S5, Data S1 |
|  | training/Negative/HD-120720-58 | HD-120720-58_005.dat | PCR negative saliva for training | Figs. 3a-3c, 3f, 3h, 3i, S13, S14, Table S5, Data S1 |
|  | training/Negative/HD-120720-7 | HD-120720-7_005.dat | PCR negative saliva for training | Figs. 3a-3c, 3f, 3h, 3i, S13, S14, Table S5, Data S1 |
|  | training/Positive/F15 day1 | F15 day1_009.dat | PCR positive saliva for training | Figs. 3a-3c, 3f, 3h, 3i, S13, S14, Table S5, Data S1 |
|  | training/Positive/F16 day1 | F16 day1_004.dat  F16 day1_005.dat  F16 day1_007.dat  F16 day1_008.dat | PCR positive saliva for training | Figs. 3a-3c, 3f, 3h, 3i, S13, S14, Table S5, Data S1 |
|  | training/Positive/F5 day5 | F5 day5_009.dat | PCR positive saliva for training | Figs. 3a-3c, 3f, 3h, 3i, S13, S14, Table S5, Data S1 |
| Saliva(n=100 HO)_I-t data-20210209T093219Z-005.zip | test/Negative/HD-112720-30 | HD-112720-30_001.dat | PCR negative saliva for test | Figs. 3g, 3j, 3k S15, Table S6, Data S2 |
|  | test/Negative/HD-112720-45 | HD-112720-45_005.dat | PCR negative saliva for test | Figs. 3g, 3j, 3k S15, Table S6, Data S2 |
|  | test/Negative/HD-113020-33 | HD-113020-33_005.dat | PCR negative saliva for test | Figs. 3g, 3j, 3k S15, Table S6, Data S2 |
|  | test/Negative/HD-120220-20 | HD-120220-20_004.dat | PCR negative saliva for test | Figs. 3g, 3j, 3k S15, Table S6, Data S2 |
|  | test/Negative/HD-120220-57 | HD-120220-57_005.dat | PCR negative saliva for test | Figs. 3g, 3j, 3k S15, Table S6, Data S2 |
|  | test/Negative/HD-120220-58 | HD-120220-58_004.dat | PCR negative saliva for test | Figs. 3g, 3j, 3k S15, Table S6, Data S2 |
|  | test/Negative/HD-120720-22 | HD-120720-22_005.dat | PCR negative saliva for test | Figs. 3g, 3j, 3k S15, Table S6, Data S2 |
|  | test/Negative/HD-120720-28 | HD-120720-28_003.dat | PCR negative saliva for test | Figs. 3g, 3j, 3k S15, Table S6, Data S2 |
|  | test/Positive/AS-2-2-bias+01_BK-1154_045fil_TI | AS-2-2-bias+01_BK-1154_045fil_TI_006.dat | PCR positive saliva for test | Figs. 3g, 3j, 3k S15, Table S6, Data S2 |
|  | test/Positive/AS-2-2-bias+01_BK-958_045fil_TI_1st | AS-2-2-bias+01_BK-958_045fil_TI_1st_009.dat | PCR positive saliva for test | Figs. 3g, 3j, 3k S15, Table S6, Data S2 |
|  | test/Positive/AS-2-2-bias+01_BK-972_045fil_TI_1st | AS-2-2-bias+01_BK-972_045fil_TI_1st_009.dat | PCR positive saliva for test | Figs. 3g, 3j, 3k S15, Table S6, Data S2 |
|  | test/Positive/F7 day14 | F7 day14_002.dat | PCR positive saliva for test | Figs. 3g, 3j, 3k S15, Table S6, Data S2 |
|  | training/Negative/HD-112720-29 | HD-112720-29_001.dat | PCR negative saliva for training | Figs. 3a-3c, 3f, 3h, 3i, S13, S14, Table S5, Data S1 |
|  | training/Negative/HD-113020-5 | HD-113020-5_002.dat | PCR negative saliva for training | Figs. 3a-3c, 3f, 3h, 3i, S13, S14, Table S5, Data S1 |
|  | training/Negative/HD-120220-19 | HD-120220-19_004.dat | PCR negative saliva for training | Figs. 3a-3c, 3f, 3h, 3i, S13, S14, Table S5, Data S1 |
|  | training/Negative/HD-120220-21 | HD-120220-21_001.dat  HD-120220-21_002.dat  HD-120220-21_003.dat  HD-120220-21_004.dat | PCR negative saliva for training | Figs. 3a-3c, 3f, 3h, 3i, S13, S14, Table S5, Data S1 |
|  | training/Negative/HD-120220-28 | HD-120220-28_001.dat  HD-120220-28_002.dat  HD-120220-28_003.dat  HD-120220-28_004.dat | PCR negative saliva for training | Figs. 3a-3c, 3f, 3h, 3i, S13, S14, Table S5, Data S1 |
|  | training/Negative/HD-120420-33 | HD-120420-33_005.dat | PCR negative saliva for training | Figs. 3a-3c, 3f, 3h, 3i, S13, S14, Table S5, Data S1 |
|  | training/Negative/HD-120720-12 | HD-120720-12_001.dat  HD-120720-12_002.dat | PCR negative saliva for training | Figs. 3a-3c, 3f, 3h, 3i, S13, S14, Table S5, Data S1 |
|  | training/Negative/HD-120720-58 | HD-120720-58_003.dat  HD-120720-58_004.dat | PCR negative saliva for training | Figs. 3a-3c, 3f, 3h, 3i, S13, S14, Table S5, Data S1 |
|  | training/Positive/AS-2-2-bias+01_BK-1099_045fil_TI | AS-2-2-bias+01_BK-1099_045fil_TI_003.dat | PCR positive saliva for training | Figs. 3a-3c, 3f, 3h, 3i, S13, S14, Table S5, Data S1 |
|  | training/Positive/AS-2-2-bias+01_BK-948_045fil_TI_1st | AS-2-2-bias+01_BK-948_045fil_TI_1st_004.dat | PCR positive saliva for training | Figs. 3a-3c, 3f, 3h, 3i, S13, S14, Table S5, Data S1 |
|  | training/Positive/AS-2-2-bias+01_BK-954_045fil_TI_1st | AS-2-2-bias+01_BK-954_045fil_TI_1st_005.dat | PCR positive saliva for training | Figs. 3a-3c, 3f, 3h, 3i, S13, S14, Table S5, Data S1 |
|  | training/Positive/F15 day1 | F15 day1_006.dat  F15 day1_007.dat | PCR positive saliva for training | Figs. 3a-3c, 3f, 3h, 3i, S13, S14, Table S5, Data S1 |
|  | training/Positive/F4 day7 | F4 day7_008.dat | PCR positive saliva for training | Figs. 3a-3c, 3f, 3h, 3i, S13, S14, Table S5, Data S1 |
|  | training/Positive/F5 day5 | F5 day5_002.dat | PCR positive saliva for training | Figs. 3a-3c, 3f, 3h, 3i, S13, S14, Table S5, Data S1 |
| Saliva(n=100 HO)_I-t data-20210209T093219Z-006.zip | test/Negative/HD-113020-2 | HD-113020-2_005.dat | PCR negative saliva for test | Figs. 3g, 3j, 3k S15, Table S6, Data S2 |
|  | test/Negative/HD-120220-12 | HD-120220-12_001.dat | PCR negative saliva for test | Figs. 3g, 3j, 3k S15, Table S6, Data S2 |
|  | test/Negative/HD-120720-28 | HD-120720-28_001.dat  HD-120720-28_002.dat | PCR negative saliva for test | Figs. 3g, 3j, 3k S15, Table S6, Data S2 |
|  | test/Positive/F10 day3 | F10 day3_001.dat | PCR positive saliva for test | Figs. 3g, 3j, 3k S15, Table S6, Data S2 |
|  | test/Positive/F11 day1 | F11 day1_007.dat  F11 day1_009.dat | PCR positive saliva for test | Figs. 3g, 3j, 3k S15, Table S6, Data S2 |
|  | test/Positive/F3 day | F3 day3_007.dat | PCR positive saliva for test | Figs. 3g, 3j, 3k S15, Table S6, Data S2 |
|  | test/Positive/F5 day3 | F5 day3_007.dat | PCR positive saliva for test | Figs. 3g, 3j, 3k S15, Table S6, Data S2 |
|  | training/Negative/HD-112720-23 | HD-112720-23_005.dat | PCR negative saliva for training | Figs. 3a-3c, 3f, 3h, 3i, S13, S14, Table S5, Data S1 |
|  | training/Negative/HD-112720-41 | HD-112720-41_003.dat | PCR negative saliva for training | Figs. 3a-3c, 3f, 3h, 3i, S13, S14, Table S5, Data S1 |
|  | training/Negative/HD-112720-46 | HD-112720-46_005.dat | PCR negative saliva for training | Figs. 3a-3c, 3f, 3h, 3i, S13, S14, Table S5, Data S1 |
|  | training/Negative/HD-113020-43 | HD-113020-43_002.dat | PCR negative saliva for training | Figs. 3a-3c, 3f, 3h, 3i, S13, S14, Table S5, Data S1 |
|  | training/Negative/HD-120220-19 | HD-120220-19_001.dat  HD-120220-19_002.dat  HD-120220-19_003.dat | PCR negative saliva for training | Figs. 3a-3c, 3f, 3h, 3i, S13, S14, Table S5, Data S1 |
|  | training/Negative/HD-120420-44 | HD-120420-44_005.dat | PCR negative saliva for training | Figs. 3a-3c, 3f, 3h, 3i, S13, S14, Table S5, Data S1 |
|  | training/Negative/HD-120720-58 | HD-120720-58_001.dat  HD-120720-58_002.dat | PCR negative saliva for training | Figs. 3a-3c, 3f, 3h, 3i, S13, S14, Table S5, Data S1 |
|  | training/Positive/F1 day5 | F1 day5_003.dat | PCR positive saliva for training | Figs. 3a-3c, 3f, 3h, 3i, S13, S14, Table S5, Data S1 |
|  | training/Positive/F15 day1 | F15 day1_001.dat  F15 day1_003.dat  F15 day1_004.dat  F15 day1_008.dat | PCR positive saliva for training | Figs. 3a-3c, 3f, 3h, 3i, S13, S14, Table S5, Data S1 |
|  | training/Positive/F5 day1 | F5 day1_002.dat | PCR positive saliva for training | Figs. 3a-3c, 3f, 3h, 3i, S13, S14, Table S5, Data S1 |
|  | training/Positive/F8 day1 | F8 day1_002.dat  F8 day1_005.dat  F8 day1_007.dat  F8 day1_009.dat | PCR positive saliva for training | Figs. 3a-3c, 3f, 3h, 3i, S13, S14, Table S5, Data S1 |
| Saliva(n=100 HO)_I-t data-20210209T093219Z-007.zip | test/Negative/HD-113020-25 | HD-113020-25_005.dat | PCR negative saliva for test | Figs. 3g, 3j, 3k S15, Table S6, Data S2 |
|  | test/Negative/HD-113020-27 | HD-113020-27_005.dat | PCR negative saliva for test | Figs. 3g, 3j, 3k S15, Table S6, Data S2 |
|  | test/Negative/HD-120220-6 | HD-120220-6_004.dat | PCR negative saliva for test | Figs. 3g, 3j, 3k S15, Table S6, Data S2 |
|  | test/Negative/HD-120420-18 | HD-120420-18_001.dat | PCR negative saliva for test | Figs. 3g, 3j, 3k S15, Table S6, Data S2 |
|  | test/Negative/HD-120420-43 | HD-120420-43_005.dat | PCR negative saliva for test | Figs. 3g, 3j, 3k S15, Table S6, Data S2 |
|  | test/Negative/HD-120720-10 | HD-120720-10_005.dat | PCR negative saliva for test | Figs. 3g, 3j, 3k S15, Table S6, Data S2 |
|  | test/Negative/HD-120720-11 | HD-120720-11_001.dat | PCR negative saliva for test | Figs. 3g, 3j, 3k S15, Table S6, Data S2 |
|  | test/Positive/F1 | F1_007.dat | PCR positive saliva for test | Figs. 3g, 3j, 3k S15, Table S6, Data S2 |
|  | test/Positive/F17 day1 | F17 day1_006.dat | PCR positive saliva for test | Figs. 3g, 3j, 3k S15, Table S6, Data S2 |
|  | training/Negative/HD-113020-3 | HD-113020-3_004.dat | PCR negative saliva for training | Figs. 3a-3c, 3f, 3h, 3i, S13, S14, Table S5, Data S1 |
|  | training/Negative/HD-113020-8 | HD-113020-8_005.dat | PCR negative saliva for training | Figs. 3a-3c, 3f, 3h, 3i, S13, S14, Table S5, Data S1 |
|  | training/Negative/HD-120420-33 | HD-120420-33_001.dat  HD-120420-33_002.dat  HD-120420-33_003.dat  HD-120420-33_004.dat | PCR negative saliva for training | Figs. 3a-3c, 3f, 3h, 3i, S13, S14, Table S5, Data S1 |
|  | training/Negative/HD-120720-17 | HD-120720-17_001.dat  HD-120720-17_002.dat  HD-120720-17_003.dat  HD-120720-17_004.dat | PCR negative saliva for training | Figs. 3a-3c, 3f, 3h, 3i, S13, S14, Table S5, Data S1 |
|  | training/Negative/HD-120720-7 | HD-120720-7_004.dat | PCR negative saliva for training | Figs. 3a-3c, 3f, 3h, 3i, S13, S14, Table S5, Data S1 |
|  | training/Positive/AS-2-2-bias+01_BK-948_045fil_TI_1st | AS-2-2-bias+01_BK-948_045fil_TI_1st_003.dat | PCR positive saliva for training | Figs. 3a-3c, 3f, 3h, 3i, S13, S14, Table S5, Data S1 |
|  | training/Positive/AS-2-2-bias+01_BK-954_045fil_TI_1st | AS-2-2-bias+01_BK-954_045fil_TI_1st_001.dat | PCR positive saliva for training | Figs. 3a-3c, 3f, 3h, 3i, S13, S14, Table S5, Data S1 |
|  | training/Positive/F1 day5 | F1 day5_001.dat | PCR positive saliva for training | Figs. 3a-3c, 3f, 3h, 3i, S13, S14, Table S5, Data S1 |
|  | training/Positive/F15 day1 | F15 day1_002.dat  F15 day1_005.dat | PCR positive saliva for training | Figs. 3a-3c, 3f, 3h, 3i, S13, S14, Table S5, Data S1 |
|  | training/Positive/F5 day5 | F5 day5_001.dat  F5 day5_005.dat | PCR positive saliva for training | Figs. 3a-3c, 3f, 3h, 3i, S13, S14, Table S5, Data S1 |
|  | training/Positive/F8 day1 | F8 day1_001.dat  F8 day1_003.dat  F8 day1_004.dat  F8 day1_006.dat | PCR positive saliva for training | Figs. 3a-3c, 3f, 3h, 3i, S13, S14, Table S5, Data S1 |
| Saliva(n=100 HO)_I-t data-20210209T093219Z-008.zip | test/Negative/HD-112720-32 | HD-112720-32_004.dat | PCR negative saliva for test | Figs. 3g, 3j, 3k S15, Table S6, Data S2 |
|  | test/Negative/HD-113020-23 | HD-113020-23_004.dat | PCR negative saliva for test | Figs. 3g, 3j, 3k S15, Table S6, Data S2 |
|  | test/Negative/HD-120220-37 | HD-120220-37_003.dat | PCR negative saliva for test | Figs. 3g, 3j, 3k S15, Table S6, Data S2 |
|  | test/Negative/HD-120720-10 | HD-120720-10_001.dat  HD-120720-10_002.dat  HD-120720-10_003.dat  HD-120720-10_004.dat | PCR negative saliva for test | Figs. 3g, 3j, 3k S15, Table S6, Data S2 |
|  | test/Negative/HD-120720-11 | HD-120720-11_002.dat  HD-120720-11_003.dat | PCR negative saliva for test | Figs. 3g, 3j, 3k S15, Table S6, Data S2 |
|  | test/Negative/HD-120720-33 | HD-120720-33_005.dat | PCR negative saliva for test | Figs. 3g, 3j, 3k S15, Table S6, Data S2 |
|  | test/Positive/AS-2-2-bias+01_BK-1095_045fil_TI | AS-2-2-bias+01_BK-1095_045fil_TI_001.dat | PCR positive saliva for test | Figs. 3g, 3j, 3k S15, Table S6, Data S2 |
|  | test/Positive/F11 day1 | F11 day1_004.dat | PCR positive saliva for test | Figs. 3g, 3j, 3k S15, Table S6, Data S2 |
|  | test/Positive/F13 day1 | F13 day1_009.dat | PCR positive saliva for test | Figs. 3g, 3j, 3k S15, Table S6, Data S2 |
|  | test/Positive/F17 day1 | F17 day1_001.dat | PCR positive saliva for test | Figs. 3g, 3j, 3k S15, Table S6, Data S2 |
|  | test/Positive/F3 day3 | F3 day3_001.dat | PCR positive saliva for test | Figs. 3g, 3j, 3k S15, Table S6, Data S2 |
|  | test/Positive/F5 day14 | F5 day14_009.dat | PCR positive saliva for test | Figs. 3g, 3j, 3k S15, Table S6, Data S2 |
|  | test/Positive/F5 day3 | F5 day3_008.dat | PCR positive saliva for test | Figs. 3g, 3j, 3k S15, Table S6, Data S2 |
|  | training/Negative/HD-112720-10 | HD-112720-10_005.dat | PCR negative saliva for training | Figs. 3a-3c, 3f, 3h, 3i, S13, S14, Table S5, Data S1 |
|  | training/Negative/HD-112720-41 | HD-112720-41_005.dat | PCR negative saliva for training | Figs. 3a-3c, 3f, 3h, 3i, S13, S14, Table S5, Data S1 |
|  | training/Negative/HD-112720-57 | HD-112720-57_005.dat | PCR negative saliva for training | Figs. 3a-3c, 3f, 3h, 3i, S13, S14, Table S5, Data S1 |
|  | training/Negative/HD-112720-58 | HD-112720-58_002.dat | PCR negative saliva for training | Figs. 3a-3c, 3f, 3h, 3i, S13, S14, Table S5, Data S1 |
|  | training/Negative/HD-120420-44 | HD-120420-44_002.dat  HD-120420-44_003.dat  HD-120420-44_004.dat | PCR negative saliva for training | Figs. 3a-3c, 3f, 3h, 3i, S13, S14, Table S5, Data S1 |
|  | training/Negative/HD-120720-46 | HD-120720-46_002.dat  HD-120720-46_004.dat | PCR negative saliva for training | Figs. 3a-3c, 3f, 3h, 3i, S13, S14, Table S5, Data S1 |
|  | training/Negative/HD-120720-7 | HD-120720-7_001.dat  HD-120720-7_002.dat  HD-120720-7_003.dat | PCR negative saliva for training | Figs. 3a-3c, 3f, 3h, 3i, S13, S14, Table S5, Data S1 |
|  | training/Positive/AS-2-2-bias+01_BK-1117_045fil_TI | AS-2-2-bias+01_BK-1117_045fil_TI_005.dat | PCR positive saliva for training | Figs. 3a-3c, 3f, 3h, 3i, S13, S14, Table S5, Data S1 |
|  | training/Positive/AS-2-2-bias+01_BK-991_045fil_TI_1st | AS-2-2-bias+01_BK-991_045fil_TI_1st_008.dat | PCR positive saliva for training | Figs. 3a-3c, 3f, 3h, 3i, S13, S14, Table S5, Data S1 |
|  | training/Positive/F1 day3 | F1 day3_003.dat | PCR positive saliva for training | Figs. 3a-3c, 3f, 3h, 3i, S13, S14, Table S5, Data S1 |
|  | training/Positive/F5 day1 | F5 day1_007.dat | PCR positive saliva for training | Figs. 3a-3c, 3f, 3h, 3i, S13, S14, Table S5, Data S1 |
|  | training/Positive/F8 day1 | F8 day1_008.dat | PCR positive saliva for training | Figs. 3a-3c, 3f, 3h, 3i, S13, S14, Table S5, Data S1 |
| Saliva(n=100 HO)_I-t data-20210209T093219Z-009.zip | test/Negative/HD-113020-2 | HD-113020-2_004.dat | PCR negative saliva for test | Figs. 3g, 3j, 3k S15, Table S6, Data S2 |
|  | test/Negative/HD-113020-36 | HD-113020-36_005.dat | PCR negative saliva for test | Figs. 3g, 3j, 3k S15, Table S6, Data S2 |
|  | test/Negative/HD-120220-12 | HD-120220-12_005.dat | PCR negative saliva for test | Figs. 3g, 3j, 3k S15, Table S6, Data S2 |
|  | test/Negative/HD-120220-20 | HD-120220-20_002.dat | PCR negative saliva for test | Figs. 3g, 3j, 3k S15, Table S6, Data S2 |
|  | test/Negative/HD-120220-37 | HD-120220-37_005.dat | PCR negative saliva for test | Figs. 3g, 3j, 3k S15, Table S6, Data S2 |
|  | test/Negative/HD-120420-6 | HD-120420-6_005.dat | PCR negative saliva for test | Figs. 3g, 3j, 3k S15, Table S6, Data S2 |
|  | test/Negative/HD-120720-22 | HD-120720-22_001.dat  HD-120720-22_002.dat  HD-120720-22_003.dat  HD-120720-22_004.dat | PCR negative saliva for test | Figs. 3g, 3j, 3k S15, Table S6, Data S2 |
|  | test/Positive/AS-2-2-bias+01_BK-964_045fil_TI_3rd | AS-2-2-bias+01_BK-964_045fil_TI_3rd_009.dat | PCR positive saliva for test | Figs. 3g, 3j, 3k S15, Table S6, Data S2 |
|  | test/Positive/AS-2-2-bias+01_BK-967_045fil_TI_1st | AS-2-2-bias+01_BK-967_045fil_TI_1st_009.dat | PCR positive saliva for test | Figs. 3g, 3j, 3k S15, Table S6, Data S2 |
|  | test/Positive/F11 day1 | F11 day1_006.dat | PCR positive saliva for test | Figs. 3g, 3j, 3k S15, Table S6, Data S2 |
|  | test/Positive/F2 day14 | F2 day14_008.dat | PCR positive saliva for test | Figs. 3g, 3j, 3k S15, Table S6, Data S2 |
|  | test/Positive/F5 day7 | F5 day7_002.dat | PCR positive saliva for test | Figs. 3g, 3j, 3k S15, Table S6, Data S2 |
|  | test/Positive/ F9 day | F9 day1_008.dat | PCR positive saliva for test | Figs. 3g, 3j, 3k S15, Table S6, Data S2 |
|  | training/Negative/HD-112720-46 | HD-112720-46_001.dat  HD-112720-46_002.dat  HD-112720-46_003.dat  HD-112720-46_004.dat | PCR negative saliva for training | Figs. 3a-3c, 3f, 3h, 3i, S13, S14, Table S5, Data S1 |
|  | training/Negative/HD-112720-57 | HD-112720-57_002.dat  HD-112720-57_004.dat | PCR negative saliva for training | Figs. 3a-3c, 3f, 3h, 3i, S13, S14, Table S5, Data S1 |
|  | training/Negative/HD-113020-5 | HD-113020-5_003.dat  HD-113020-5_004.dat | PCR negative saliva for training | Figs. 3a-3c, 3f, 3h, 3i, S13, S14, Table S5, Data S1 |
|  | training/Negative/HD-113020-8 | HD-113020-8_004.dat | PCR negative saliva for training | Figs. 3a-3c, 3f, 3h, 3i, S13, S14, Table S5, Data S1 |
|  | training/Negative/HD-120420-44 | HD-120420-44_001.dat | PCR negative saliva for training | Figs. 3a-3c, 3f, 3h, 3i, S13, S14, Table S5, Data S1 |
|  | training/Negative/HD-120720-46 | HD-120720-46_001.dat  HD-120720-46_003.dat | PCR negative saliva for training | Figs. 3a-3c, 3f, 3h, 3i, S13, S14, Table S5, Data S1 |
|  | training/Positive/AS-2-2-bias+01_BK-1117_045fil_TI | AS-2-2-bias+01_BK-1117_045fil_TI_001.dat | PCR positive saliva for training | Figs. 3a-3c, 3f, 3h, 3i, S13, S14, Table S5, Data S1 |
|  | training/Positive/AS-2-2-bias+01_BK-970_045fil_TI_1st | AS-2-2-bias+01_BK-970_045fil_TI_1st_001.dat | PCR positive saliva for training | Figs. 3a-3c, 3f, 3h, 3i, S13, S14, Table S5, Data S1 |
|  | training/Positive/F2 | F2_002.dat | PCR positive saliva for training | Figs. 3a-3c, 3f, 3h, 3i, S13, S14, Table S5, Data S1 |
|  | training/Positive/F2 day3 | F2 day3_001.dat | PCR positive saliva for training | Figs. 3a-3c, 3f, 3h, 3i, S13, S14, Table S5, Data S1 |
|  | training/Positive/F2 day5 | F2 day5_006.dat | PCR positive saliva for training | Figs. 3a-3c, 3f, 3h, 3i, S13, S14, Table S5, Data S1 |
|  | training/Positive/F2 day7 | F2 day7_001.dat | PCR positive saliva for training | Figs. 3a-3c, 3f, 3h, 3i, S13, S14, Table S5, Data S1 |
| Saliva(n=100 HO)_I-t data-20210209T093219Z-010.zip | test/Negative/HD-112720-32 | HD-112720-32_003.dat | PCR negative saliva for test | Figs. 3g, 3j, 3k S15, Table S6, Data S2 |
|  | test/Negative/HD-113020-45 | HD-113020-45_002.dat | PCR negative saliva for test | Figs. 3g, 3j, 3k S15, Table S6, Data S2 |
|  | test/Negative/HD-120220-12 | HD-120220-12_003.dat | PCR negative saliva for test | Figs. 3g, 3j, 3k S15, Table S6, Data S2 |
|  | test/Negative/HD-120220-7 | HD-120220-7_005.dat | PCR negative saliva for test | Figs. 3g, 3j, 3k S15, Table S6, Data S2 |
|  | test/Negative/HD-120420-13 | HD-120420-13_001.dat | PCR negative saliva for test | Figs. 3g, 3j, 3k S15, Table S6, Data S2 |
|  | test/Positive/AS-2-2-bias+01_BK-1096_045fil_TI | AS-2-2-bias+01_BK-1096_045fil_TI_002.dat | PCR positive saliva for test | Figs. 3g, 3j, 3k S15, Table S6, Data S2 |
|  | test/Positive/AS-2-2-bias+01_BK-1149_045fil_TI | AS-2-2-bias+01_BK-1149_045fil_TI_001.dat | PCR positive saliva for test | Figs. 3g, 3j, 3k S15, Table S6, Data S2 |
|  | test/Positive/F11 day1 | F11 day1_001.dat | PCR positive saliva for test | Figs. 3g, 3j, 3k S15, Table S6, Data S2 |
|  | test/Positive/F17 day1 | F17 day1_005.dat | PCR positive saliva for test | Figs. 3g, 3j, 3k S15, Table S6, Data S2 |
|  | test/Positive/F5 day3 | F5 day3_003.dat | PCR positive saliva for test | Figs. 3g, 3j, 3k S15, Table S6, Data S2 |
|  | training/Negative/HD-112720-23 | HD-112720-23_001.dat  HD-112720-23_002.dat  HD-112720-23_003.dat  HD-112720-23_004.dat | PCR negative saliva for training | Figs. 3a-3c, 3f, 3h, 3i, S13, S14, Table S5, Data S1 |
|  | training/Negative/HD-112720-44 | HD-112720-44_001.dat  HD-112720-44_002.dat  HD-112720-44_003.dat  HD-112720-44_004.dat | PCR negative saliva for training | Figs. 3a-3c, 3f, 3h, 3i, S13, S14, Table S5, Data S1 |
|  | training/Negative/HD-112720-57 | HD-112720-57_001.dat  HD-112720-57_003.dat | PCR negative saliva for training | Figs. 3a-3c, 3f, 3h, 3i, S13, S14, Table S5, Data S1 |
|  | training/Negative/HD-112720-58 | HD-112720-58_003.dat  HD-112720-58_004.dat | PCR negative saliva for training | Figs. 3a-3c, 3f, 3h, 3i, S13, S14, Table S5, Data S1 |
|  | training/Negative/HD-113020-1 | HD-113020-1_001.dat | PCR negative saliva for training | Figs. 3a-3c, 3f, 3h, 3i, S13, S14, Table S5, Data S1 |
|  | training/Negative/HD-113020-7 | HD-113020-7_005.dat | PCR negative saliva for training | Figs. 3a-3c, 3f, 3h, 3i, S13, S14, Table S5, Data S1 |
|  | training/Negative/HD-113020-8 | HD-113020-8_001.dat  HD-113020-8_002.dat  HD-113020-8_003.dat | PCR negative saliva for training | Figs. 3a-3c, 3f, 3h, 3i, S13, S14, Table S5, Data S1 |
|  | training/Negative/HD-120220-4 | HD-120220-4_005.dat | PCR negative saliva for training | Figs. 3a-3c, 3f, 3h, 3i, S13, S14, Table S5, Data S1 |
|  | training/Positive/AS-2-2-bias+01_BK-973_045fil_TI_1st | AS-2-2-bias+01_BK-973_045fil_TI_1st_004.dat | PCR positive saliva for training | Figs. 3a-3c, 3f, 3h, 3i, S13, S14, Table S5, Data S1 |
|  | training/Positive/F2 day3 | F2 day3_007.dat | PCR positive saliva for training | Figs. 3a-3c, 3f, 3h, 3i, S13, S14, Table S5, Data S1 |
| Saliva(n=100 HO)_I-t data-20210209T093219Z-011.zip | test/Negative/HD-112720-17 | HD-112720-17_001.dat | PCR negative saliva for test | Figs. 3g, 3j, 3k S15, Table S6, Data S2 |
|  | test/Negative/HD-112720-32 | HD-112720-32_001.dat | PCR negative saliva for test | Figs. 3g, 3j, 3k S15, Table S6, Data S2 |
|  | test/Negative/HD-113020-36 | HD-113020-36_002.dat  HD-113020-36_003.dat  HD-113020-36_004.dat | PCR negative saliva for test | Figs. 3g, 3j, 3k S15, Table S6, Data S2 |
|  | test/Negative/HD-120220-12 | HD-120220-12_004.dat | PCR negative saliva for test | Figs. 3g, 3j, 3k S15, Table S6, Data S2 |
|  | test/Negative/HD-120220-33 | HD-120220-33_005.dat | PCR negative saliva for test | Figs. 3g, 3j, 3k S15, Table S6, Data S2 |
|  | test/Negative/HD-120220-7 | HD-120220-7_004.dat | PCR negative saliva for test | Figs. 3g, 3j, 3k S15, Table S6, Data S2 |
|  | test/Negative/HD-120420-18 | HD-120420-18_005.dat | PCR negative saliva for test | Figs. 3g, 3j, 3k S15, Table S6, Data S2 |
|  | test/Negative/HD-120420-3 | HD-120420-3_004.dat | PCR negative saliva for test | Figs. 3g, 3j, 3k S15, Table S6, Data S2 |
|  | test/Positive/F5 day3 | F5 day3_009.dat | PCR positive saliva for test | Figs. 3g, 3j, 3k S15, Table S6, Data S2 |
|  | test/Positive/F9 day1 | F9 day1_009.dat | PCR positive saliva for test | Figs. 3g, 3j, 3k S15, Table S6, Data S2 |
|  | training/Negative/HD-112720-18 | HD-112720-18_005.dat | PCR negative saliva for training | Figs. 3a-3c, 3f, 3h, 3i, S13, S14, Table S5, Data S1 |
|  | training/Negative/HD-112720-21 | HD-112720-21_001.dat  HD-112720-21_002.dat  HD-112720-21_003.dat  HD-112720-21_004.dat | PCR negative saliva for training | Figs. 3a-3c, 3f, 3h, 3i, S13, S14, Table S5, Data S1 |
|  | training/Negative/HD-112720-29 | HD-112720-29_004.dat | PCR negative saliva for training | Figs. 3a-3c, 3f, 3h, 3i, S13, S14, Table S5, Data S1 |
|  | training/Negative/HD-113020-1 | HD-113020-1_002.dat  HD-113020-1_003.dat  HD-113020-1_004.dat | PCR negative saliva for training | Figs. 3a-3c, 3f, 3h, 3i, S13, S14, Table S5, Data S1 |
|  | training/Negative/HD-113020-3 | HD-113020-3_003.dat | PCR negative saliva for training | Figs. 3a-3c, 3f, 3h, 3i, S13, S14, Table S5, Data S1 |
|  | training/Positive/AS-2-2-bias+01_BK-1100_045fil_TI | AS-2-2-bias+01_BK-1100_045fil_TI_009.dat | PCR positive saliva for training | Figs. 3a-3c, 3f, 3h, 3i, S13, S14, Table S5, Data S1 |
|  | training/Positive/AS-2-2-bias+01_BK-1123_045fil_TI | AS-2-2-bias+01_BK-1123_045fil_TI_008.dat | PCR positive saliva for training | Figs. 3a-3c, 3f, 3h, 3i, S13, S14, Table S5, Data S1 |
|  | training/Positive/AS-2-2-bias+01_BK-948_045fil_TI_1st | AS-2-2-bias+01_BK-948_045fil_TI_1st_002.dat | PCR positive saliva for training | Figs. 3a-3c, 3f, 3h, 3i, S13, S14, Table S5, Data S1 |
|  | training/Positive/AS-2-2-bias+01_BK-992_045fil_TI_1st | AS-2-2-bias+01_BK-992_045fil_TI_1st_002.dat | PCR positive saliva for training | Figs. 3a-3c, 3f, 3h, 3i, S13, S14, Table S5, Data S1 |
|  | training/Positive/F1 day5 | F1 day5_002.dat | PCR positive saliva for training | Figs. 3a-3c, 3f, 3h, 3i, S13, S14, Table S5, Data S1 |
|  | training/Positive/F11 day3 | F11 day3_001.dat  F11 day3_003.dat  F11 day3_009.dat | PCR positive saliva for training | Figs. 3a-3c, 3f, 3h, 3i, S13, S14, Table S5, Data S1 |
|  | training/Positive/F4 day7 | F4 day7_002.dat | PCR positive saliva for training | Figs. 3a-3c, 3f, 3h, 3i, S13, S14, Table S5, Data S1 |
| Saliva(n=100 HO)_I-t data-20210209T093219Z-012.zip | test/Negative/HD-112720-1 | HD-112720-1_005.dat | PCR negative saliva for test | Figs. 3g, 3j, 3k S15, Table S6, Data S2 |
|  | test/Negative/HD-112720-35 | HD-112720-35_001.dat | PCR negative saliva for test | Figs. 3g, 3j, 3k S15, Table S6, Data S2 |
|  | test/Negative/HD-113020-17 | HD-113020-17_003.dat | PCR negative saliva for test | Figs. 3g, 3j, 3k S15, Table S6, Data S2 |
|  | test/Negative/HD-113020-2 | HD-113020-2_002.dat  HD-113020-2_003.dat | PCR negative saliva for test | Figs. 3g, 3j, 3k S15, Table S6, Data S2 |
|  | test/Negative/HD-113020-36 | HD-113020-36_001.dat | PCR negative saliva for test | Figs. 3g, 3j, 3k S15, Table S6, Data S2 |
|  | test/Negative/HD-113020-47 | HD-113020-47_005.dat | PCR negative saliva for test | Figs. 3g, 3j, 3k S15, Table S6, Data S2 |
|  | test/Negative/HD-120220-20 | HD-120220-20_005.dat | PCR negative saliva for test | Figs. 3g, 3j, 3k S15, Table S6, Data S2 |
|  | test/Negative/HD-120220-7 | HD-120220-7_001.dat  HD-120220-7_002.dat  HD-120220-7_003.dat | PCR negative saliva for test | Figs. 3g, 3j, 3k S15, Table S6, Data S2 |
|  | test/Negative/HD-120420-22 | HD-120420-22_005.dat | PCR negative saliva for test | Figs. 3g, 3j, 3k S15, Table S6, Data S2 |
|  | test/Positive/AS-2-2-bias+01_BK-958_045fil_TI_1st | AS-2-2-bias+01_BK-958_045fil_TI_1st_002.dat | PCR positive saliva for test | Figs. 3g, 3j, 3k S15, Table S6, Data S2 |
|  | test/Positive/F1 | F1_003.dat134.2 | PCR positive saliva for test | Figs. 3g, 3j, 3k S15, Table S6, Data S2 |
|  | test/Positive/F11 day1 | F11 day1_002.dat | PCR positive saliva for test | Figs. 3g, 3j, 3k S15, Table S6, Data S2 |
|  | test/Positive/F3 day3 | F3 day3_009.dat | PCR positive saliva for test | Figs. 3g, 3j, 3k S15, Table S6, Data S2 |
|  | test/Positive/F7 day14 | F7 day14_008.dat | PCR positive saliva for test | Figs. 3g, 3j, 3k S15, Table S6, Data S2 |
|  | test/Positive/F9 day1 | F9 day1_005.dat | PCR positive saliva for test | Figs. 3g, 3j, 3k S15, Table S6, Data S2 |
|  | training/Negative/HD-112720-29 | HD-112720-29_002.dat  HD-112720-29_003.dat | PCR negative saliva for training | Figs. 3a-3c, 3f, 3h, 3i, S13, S14, Table S5, Data S1 |
|  | training/Negative/HD-112720-41 | HD-112720-41_001.dat  HD-112720-41_002.dat  HD-112720-41_004.dat | PCR negative saliva for training | Figs. 3a-3c, 3f, 3h, 3i, S13, S14, Table S5, Data S1 |
|  | training/Negative/HD-113020-3 | HD-113020-3_001.dat  HD-113020-3_002.dat | PCR negative saliva for training | Figs. 3a-3c, 3f, 3h, 3i, S13, S14, Table S5, Data S1 |
|  | training/Negative/HD-113020-44 | HD-113020-44_005.dat | PCR negative saliva for training | Figs. 3a-3c, 3f, 3h, 3i, S13, S14, Table S5, Data S1 |
|  | training/Positive/AS-2-2-bias+01_BK-1100_045fil_TI | AS-2-2-bias+01_BK-1100_045fil_TI_002.dat | PCR positive saliva for training | Figs. 3a-3c, 3f, 3h, 3i, S13, S14, Table S5, Data S1 |
|  | training/Positive/AS-2-2-bias+01_BK-959_045fil_TI_1st | AS-2-2-bias+01_BK-959_045fil_TI_1st_004.dat | PCR positive saliva for training | Figs. 3a-3c, 3f, 3h, 3i, S13, S14, Table S5, Data S1 |
|  | training/Positive/F11 day3 | F11 day3_004.dat  F11 day3_006.dat  F11 day3_007.dat | PCR positive saliva for training | Figs. 3a-3c, 3f, 3h, 3i, S13, S14, Table S5, Data S1 |
|  | training/Positive/F2 day7 | F2 day7_002.dat | PCR positive saliva for training | Figs. 3a-3c, 3f, 3h, 3i, S13, S14, Table S5, Data S1 |
| Saliva(n=100 HO)_I-t data-20210209T093219Z-013.zip | test/Negative/HD-112720-32 | HD-112720-32_005.dat | PCR negative saliva for test | Figs. 3g, 3j, 3k S15, Table S6, Data S2 |
|  | test/Negative/HD-112720-35 | HD-112720-35_005.dat | PCR negative saliva for test | Figs. 3g, 3j, 3k S15, Table S6, Data S2 |
|  | test/Negative/HD-113020-17 | HD-113020-17_005.dat | PCR negative saliva for test | Figs. 3g, 3j, 3k S15, Table S6, Data S2 |
|  | test/Negative/HD-113020-2 | HD-113020-2_001.dat | PCR negative saliva for test | Figs. 3g, 3j, 3k S15, Table S6, Data S2 |
|  | test/Negative/HD-113020-23 | HD-113020-23_003.dat | PCR negative saliva for test | Figs. 3g, 3j, 3k S15, Table S6, Data S2 |
|  | test/Negative/HD-113020-25 | HD-113020-25_004.dat | PCR negative saliva for test | Figs. 3g, 3j, 3k S15, Table S6, Data S2 |
|  | test/Negative/HD-113020-45 | HD-113020-45_001.dat  HD-113020-45_003.dat  HD-113020-45_004.dat | PCR negative saliva for test | Figs. 3g, 3j, 3k S15, Table S6, Data S2 |
|  | test/Negative/HD-120220-33 | HD-120220-33_001.dat  HD-120220-33_002.dat  HD-120220-33_003.dat  HD-120220-33_004.dat | PCR negative saliva for test | Figs. 3g, 3j, 3k S15, Table S6, Data S2 |
|  | test/Negative/HD-120220-37 | HD-120220-37_002.dat  HD-120220-37_004.dat | PCR negative saliva for test | Figs. 3g, 3j, 3k S15, Table S6, Data S2 |
|  | test/Negative/HD-120220-57 | HD-120220-57_001.dat  HD-120220-57_004.dat | PCR negative saliva for test | Figs. 3g, 3j, 3k S15, Table S6, Data S2 |
|  | test/Negative/HD-120420-43 | HD-120420-43_001.dat | PCR negative saliva for test | Figs. 3g, 3j, 3k S15, Table S6, Data S2 |
|  | test/Positive/AS-2-2-bias+01_BK-965_045fil_TI_1st | AS-2-2-bias+01_BK-965_045fil_TI_1st_007.dat | PCR positive saliva for test | Figs. 3g, 3j, 3k S15, Table S6, Data S2 |
|  | test/Positive/F7 day14 | F7 day14_003.dat | PCR positive saliva for test | Figs. 3g, 3j, 3k S15, Table S6, Data S2 |
|  | training/Negative/HD-113020-28 | HD-113020-28_004.dat134.2 MB | PCR negative saliva for training | Figs. 3a-3c, 3f, 3h, 3i, S13, S14, Table S5, Data S1 |
|  | training/Negative/HD-113020-7 | HD-113020-7_004.dat | PCR negative saliva for training | Figs. 3a-3c, 3f, 3h, 3i, S13, S14, Table S5, Data S1 |
|  | training/Positive/AS-2-2-bias+01_BK-959_045fil_TI_1st | AS-2-2-bias+01_BK-959_045fil_TI_1st_008.dat | PCR positive saliva for training | Figs. 3a-3c, 3f, 3h, 3i, S13, S14, Table S5, Data S1 |
|  | training/Positive/F1 day5 | F1 day5_005.dat | PCR positive saliva for training | Figs. 3a-3c, 3f, 3h, 3i, S13, S14, Table S5, Data S1 |
|  | training/Positive/F11 day3 | F11 day3_002.dat  F11 day3_005.dat | PCR positive saliva for training | Figs. 3a-3c, 3f, 3h, 3i, S13, S14, Table S5, Data S1 |
|  | training/Positive/F2 | F2_007.dat | PCR positive saliva for training | Figs. 3a-3c, 3f, 3h, 3i, S13, S14, Table S5, Data S1 |
| Saliva(n=100 HO)_I-t data-20210209T093219Z-014.zip | test/Negative/HD-112720-45 | HD-112720-45_004.dat | PCR negative saliva for test | Figs. 3g, 3j, 3k S15, Table S6, Data S2 |
|  | test/Negative/HD-113020-23 | HD-113020-23_001.dat  HD-113020-23_002.dat | PCR negative saliva for test | Figs. 3g, 3j, 3k S15, Table S6, Data S2 |
|  | test/Negative/HD-113020-25 | HD-113020-25_001.dat  HD-113020-25_002.dat  HD-113020-25_003.dat | PCR negative saliva for test | Figs. 3g, 3j, 3k S15, Table S6, Data S2 |
|  | test/Negative/HD-113020-27 | HD-113020-27_001.dat  HD-113020-27_002.dat  HD-113020-27_003.dat  HD-113020-27_004.dat | PCR negative saliva for test | Figs. 3g, 3j, 3k S15, Table S6, Data S2 |
|  | test/Negative/HD-113020-47 | HD-113020-47_001.dat  HD-113020-47_003.dat  HD-113020-47_004.dat | PCR negative saliva for test | Figs. 3g, 3j, 3k S15, Table S6, Data S2 |
|  | test/Negative/HD-120220-12 | HD-120220-12_002.dat | PCR negative saliva for test | Figs. 3g, 3j, 3k S15, Table S6, Data S2 |
|  | test/Negative/HD-120220-20 | HD-120220-20_003.dat | PCR negative saliva for test | Figs. 3g, 3j, 3k S15, Table S6, Data S2 |
|  | test/Negative/HD-120220-37 | HD-120220-37_001.dat | PCR negative saliva for test | Figs. 3g, 3j, 3k S15, Table S6, Data S2 |
|  | test/Negative/HD-120420-13 | HD-120420-13_005.dat | PCR negative saliva for test | Figs. 3g, 3j, 3k S15, Table S6, Data S2 |
|  | test/Negative/HD-120720-4 | HD-120720-4_002.dat | PCR negative saliva for test | Figs. 3g, 3j, 3k S15, Table S6, Data S2 |
|  | test/Positive/AS-2-2-bias+01_BK-965_045fil_TI_1st | AS-2-2-bias+01_BK-965_045fil_TI_1st_009.dat | PCR positive saliva for test | Figs. 3g, 3j, 3k S15, Table S6, Data S2 |
|  | test/Positive/AS-2-2-bias+01_BK-987_045fil_TI_1st | AS-2-2-bias+01_BK-987_045fil_TI_1st_009.dat | PCR positive saliva for test | Figs. 3g, 3j, 3k S15, Table S6, Data S2 |
|  | test/Positive/F10 day1① | F10 day1①_002.dat | PCR positive saliva for test | Figs. 3g, 3j, 3k S15, Table S6, Data S2 |
|  | test/Positive/F13 day1 | F13 day1_001.dat | PCR positive saliva for test | Figs. 3g, 3j, 3k S15, Table S6, Data S2 |
|  | test/Positive/F17 day1 | F17 day1_009.dat | PCR positive saliva for test | Figs. 3g, 3j, 3k S15, Table S6, Data S2 |
|  | training/Positive/AS-2-2-bias+01_BK-1094_045fil_TI | AS-2-2-bias+01_BK-1094_045fil_TI_006.dat | PCR positive saliva for training | Figs. 3a-3c, 3f, 3h, 3i, S13, S14, Table S5, Data S1 |
|  | training/Positive/AS-2-2-bias+01_BK-954_045fil_TI_1st | AS-2-2-bias+01_BK-954_045fil_TI_1st_009.dat | PCR positive saliva for training | Figs. 3a-3c, 3f, 3h, 3i, S13, S14, Table S5, Data S1 |
|  | training/Positive/AS-2-2-bias+01_BK-989_045fil_TI_1st | AS-2-2-bias+01_BK-989_045fil_TI_1st_002.dat | PCR positive saliva for training | Figs. 3a-3c, 3f, 3h, 3i, S13, S14, Table S5, Data S1 |
|  | training/Positive/F4 day7 | F4 day7_006.dat | PCR positive saliva for training | Figs. 3a-3c, 3f, 3h, 3i, S13, S14, Table S5, Data S1 |
| Saliva(n=100 HO)_I-t data-20210209T093219Z-015.zip | test/Negative/HD-112720-45 | HD-112720-45_001.dat | PCR negative saliva for test | Figs. 3g, 3j, 3k S15, Table S6, Data S2 |
|  | test/Negative/HD-113020-17 | HD-113020-17_001.dat  HD-113020-17_002.dat | PCR negative saliva for test | Figs. 3g, 3j, 3k S15, Table S6, Data S2 |
|  | test/Negative/HD-120220-20 | HD-120220-20_001.dat | PCR negative saliva for test | Figs. 3g, 3j, 3k S15, Table S6, Data S2 |
|  | test/Negative/HD-120420-18 | HD-120420-18_003.dat  HD-120420-18_004.dat | PCR negative saliva for test | Figs. 3g, 3j, 3k S15, Table S6, Data S2 |
|  | test/Negative/HD-120720-4 | HD-120720-4_005.dat | PCR negative saliva for test | Figs. 3g, 3j, 3k S15, Table S6, Data S2 |
|  | test/Positive/AS-2-2-bias+01_BK-984_045fil_TI_1st | AS-2-2-bias+01_BK-984_045fil_TI_1st_003.dat | PCR positive saliva for test | Figs. 3g, 3j, 3k S15, Table S6, Data S2 |
|  | test/Positive/F1 | F1_004.dat | PCR positive saliva for test | Figs. 3g, 3j, 3k S15, Table S6, Data S2 |
|  | test/Positive/F10 day3 | F10 day3_005.dat  F10 day3_006.dat  F10 day3_007.dat | PCR positive saliva for test | Figs. 3g, 3j, 3k S15, Table S6, Data S2 |
|  | test/Positive/F11 day1 | F11 day1_003.dat | PCR positive saliva for test | Figs. 3g, 3j, 3k S15, Table S6, Data S2 |
|  | test/Positive/F13 day3 | F13 day3_006.dat | PCR positive saliva for test | Figs. 3g, 3j, 3k S15, Table S6, Data S2 |
|  | test/Positive/F3 day5 | F3 day5_007.dat | PCR positive saliva for test | Figs. 3g, 3j, 3k S15, Table S6, Data S2 |
|  | test/Positive/F7 day1 | F7 day1_008.dat | PCR positive saliva for test | Figs. 3g, 3j, 3k S15, Table S6, Data S2 |
|  | training/Negative/HD-113020-44 | HD-113020-44_003.dat | PCR positive saliva for training | Figs. 3a-3c, 3f, 3h, 3i, S13, S14, Table S5, Data S1 |
|  | training/Positive/AS-2-2-bias+01_BK-1094_045fil_TI | AS-2-2-bias+01_BK-1094_045fil_TI_001.dat  AS-2-2-bias+01_BK-1094_045fil_TI_002.dat  AS-2-2-bias+01_BK-1094_045fil_TI_003.dat  AS-2-2-bias+01_BK-1094_045fil_TI_007.dat  AS-2-2-bias+01_BK-1094_045fil_TI_009.dat | PCR positive saliva for training | Figs. 3a-3c, 3f, 3h, 3i, S13, S14, Table S5, Data S1 |
|  | training/Positive/AS-2-2-bias+01_BK-948_045fil_TI_1st | AS-2-2-bias+01_BK-948_045fil_TI_1st_001.dat | PCR positive saliva for training | Figs. 3a-3c, 3f, 3h, 3i, S13, S14, Table S5, Data S1 |
|  | training/Positive/AS-2-2-bias+01_BK-954_045fil_TI_1st | AS-2-2-bias+01_BK-954_045fil_TI_1st_003.dat  AS-2-2-bias+01_BK-954_045fil_TI_1st_006.dat  AS-2-2-bias+01_BK-954_045fil_TI_1st_007.dat | PCR positive saliva for training | Figs. 3a-3c, 3f, 3h, 3i, S13, S14, Table S5, Data S1 |
|  | training/Positive/F1 day5 | F1 day5_008.dat | PCR positive saliva for training | Figs. 3a-3c, 3f, 3h, 3i, S13, S14, Table S5, Data S1 |
| Saliva(n=100 HO)_I-t data-20210209T093219Z-016.zip | test/Negative/HD-112720-30 | HD-112720-30_005.dat | PCR negative saliva for test | Figs. 3g, 3j, 3k S15, Table S6, Data S2 |
|  | test/Negative/HD-112720-32 | HD-112720-32_002.dat | PCR negative saliva for test | Figs. 3g, 3j, 3k S15, Table S6, Data S2 |
|  | test/Negative/HD-112720-39 | HD-112720-39_001.dat  HD-112720-39_003.dat  HD-112720-39_004.dat | PCR negative saliva for test | Figs. 3g, 3j, 3k S15, Table S6, Data S2 |
|  | test/Negative/HD-120420-4 | HD-120420-4_005.dat | PCR negative saliva for test | Figs. 3g, 3j, 3k S15, Table S6, Data S2 |
|  | test/Positive/F1 | F1_009.dat | PCR positive saliva for test | Figs. 3g, 3j, 3k S15, Table S6, Data S2 |
|  | test/Positive/F10 day1① | F10 day1①_005.dat | PCR positive saliva for test | Figs. 3g, 3j, 3k S15, Table S6, Data S2 |
|  | test/Positive/F10 day3 | F10 day3_002.dat  F10 day3_004.dat  F10 day3_008.dat  F10 day3_009.dat | PCR positive saliva for test | Figs. 3g, 3j, 3k S15, Table S6, Data S2 |
|  | test/Positive/F11 day1 | F11 day1_008.dat | PCR positive saliva for test | Figs. 3g, 3j, 3k S15, Table S6, Data S2 |
|  | test/Positive/F3 day5 | F3 day5_004.dat | PCR positive saliva for test | Figs. 3g, 3j, 3k S15, Table S6, Data S2 |
|  | test/Positive/F7 day5 | F7 day5_003.dat | PCR positive saliva for test | Figs. 3g, 3j, 3k S15, Table S6, Data S2 |
|  | training/Negative/HD-113020-35 | HD-113020-35_005.dat | PCR negative saliva for training | Figs. 3a-3c, 3f, 3h, 3i, S13, S14, Table S5, Data S1 |
|  | training/Positive/AS-2-2-bias+01_BK-1094_045fil_TI | AS-2-2-bias+01_BK-1094_045fil_TI_004.dat  AS-2-2-bias+01_BK-1094_045fil_TI_005.dat | PCR positive saliva for training | Figs. 3a-3c, 3f, 3h, 3i, S13, S14, Table S5, Data S1 |
|  | training/Positive/AS-2-2-bias+01_BK-948_045fil_TI_1st | AS-2-2-bias+01_BK-948_045fil_TI_1st_009.dat | PCR positive saliva for training | Figs. 3a-3c, 3f, 3h, 3i, S13, S14, Table S5, Data S1 |
|  | training/Positive/AS-2-2-bias+01_BK-954_045fil_TI_1st | AS-2-2-bias+01_BK-954_045fil_TI_1st_002.dat  AS-2-2-bias+01_BK-954_045fil_TI_1st_004.dat  AS-2-2-bias+01_BK-954_045fil_TI_1st_008.dat | PCR positive saliva for training | Figs. 3a-3c, 3f, 3h, 3i, S13, S14, Table S5, Data S1 |
|  | training/Positive/AS-2-2-bias+01_BK-970_045fil_TI_1st | AS-2-2-bias+01_BK-970_045fil_TI_1st_002.dat | PCR positive saliva for training | Figs. 3a-3c, 3f, 3h, 3i, S13, S14, Table S5, Data S1 |
|  | training/Positive/F1 day5 | F1 day5_007.dat | PCR positive saliva for training | Figs. 3a-3c, 3f, 3h, 3i, S13, S14, Table S5, Data S1 |
|  | training/Positive/F2 | F2_004.dat  F2_008.dat  F2_009.dat | PCR positive saliva for training | Figs. 3a-3c, 3f, 3h, 3i, S13, S14, Table S5, Data S1 |
|  | training/Positive/F2 day7 | F2 day7_006.dat | PCR positive saliva for training | Figs. 3a-3c, 3f, 3h, 3i, S13, S14, Table S5, Data S1 |
| Saliva(n=100 HO)_I-t data-20210209T093219Z-017.zip | test/Negative/HD-112720-17 | HD-112720-17_005.dat | PCR negative saliva for test | Figs. 3g, 3j, 3k S15, Table S6, Data S2 |
|  | test/Negative/HD-112720-39 | HD-112720-39_002.dat | PCR negative saliva for test | Figs. 3g, 3j, 3k S15, Table S6, Data S2 |
|  | test/Negative/HD-112720-45 | HD-112720-45_002.dat | PCR negative saliva for test | Figs. 3g, 3j, 3k S15, Table S6, Data S2 |
|  | test/Negative/HD-120220-36 | HD-120220-36_003.dat | PCR negative saliva for test | Figs. 3g, 3j, 3k S15, Table S6, Data S2 |
|  | test/Positive/AS-2-2-bias+01_BK-965_045fil_TI_1st | AS-2-2-bias+01_BK-965_045fil_TI_1st_003.dat | PCR positive saliva for test | Figs. 3g, 3j, 3k S15, Table S6, Data S2 |
|  | test/Positive/AS-2-2-bias+01_BK-969_045fil_TI_1st | AS-2-2-bias+01_BK-969_045fil_TI_1st_009.dat | PCR positive saliva for test | Figs. 3g, 3j, 3k S15, Table S6, Data S2 |
|  | test/Positive/AS-2-2-bias+01_BK-983_045fil_TI_1st | AS-2-2-bias+01_BK-983_045fil_TI_1st_009.dat | PCR positive saliva for test | Figs. 3g, 3j, 3k S15, Table S6, Data S2 |
|  | test/Positive/F1 | F1_001.dat  F1_002.dat  F1_006.dat  F1_008.dat | PCR positive saliva for test | Figs. 3g, 3j, 3k S15, Table S6, Data S2 |
|  | test/Positive/F11 day1 | F11 day1_005.dat | PCR positive saliva for test | Figs. 3g, 3j, 3k S15, Table S6, Data S2 |
|  | test/Positive/F5 day3 | F5 day3_001.dat  F5 day3_004.dat  F5 day3_005.dat | PCR positive saliva for test | Figs. 3g, 3j, 3k S15, Table S6, Data S2 |
|  | test/Positive/F7 day1 | F7 day1_003.dat | PCR positive saliva for test | Figs. 3g, 3j, 3k S15, Table S6, Data S2 |
|  | training/Negative/HD-113020-7 | HD-113020-7_001.dat  HD-113020-7_003.dat | PCR negative saliva for training | Figs. 3a-3c, 3f, 3h, 3i, S13, S14, Table S5, Data S1 |
|  | training/Positive/AS-2-2-bias+01_BK-1099_045fil_TI | AS-2-2-bias+01_BK-1099_045fil_TI_007.dat | PCR positive saliva for training | Figs. 3a-3c, 3f, 3h, 3i, S13, S14, Table S5, Data S1 |
|  | training/Positive/AS-2-2-bias+01_BK-973_045fil_TI_1st | AS-2-2-bias+01_BK-973_045fil_TI_1st_006.dat | PCR positive saliva for training | Figs. 3a-3c, 3f, 3h, 3i, S13, S14, Table S5, Data S1 |
|  | training/Positive/F2 | F2_003.dat  F2_005.dat  F2_006.dat | PCR positive saliva for training | Figs. 3a-3c, 3f, 3h, 3i, S13, S14, Table S5, Data S1 |
|  | training/Positive/F2 day3 | F2 day3_008.dat | PCR positive saliva for training | Figs. 3a-3c, 3f, 3h, 3i, S13, S14, Table S5, Data S1 |
|  | training/Positive/F4 day7 | F4 day7_004.dat  F4 day7_007.dat | PCR positive saliva for training | Figs. 3a-3c, 3f, 3h, 3i, S13, S14, Table S5, Data S1 |
|  | training/Positive/F8 day3 | F8 day3_003.dat | PCR positive saliva for training | Figs. 3a-3c, 3f, 3h, 3i, S13, S14, Table S5, Data S1 |
| Saliva(n=100 HO)_I-t data-20210209T093219Z-018.zip | test/Negative/HD-120420-21 | HD-120420-21_004.dat | PCR negative saliva for test | Figs. 3g, 3j, 3k S15, Table S6, Data S2 |
|  | test/Positive/AS-2-2-bias+01_BK-1103_045fil_TI | AS-2-2-bias+01_BK-1103_045fil_TI_007.dat | PCR positive saliva for test | Figs. 3g, 3j, 3k S15, Table S6, Data S2 |
|  | test/Positive/AS-2-2-bias+01_BK-1152_045fil_TI | AS-2-2-bias+01_BK-1152_045fil_TI_008.dat | PCR positive saliva for test | Figs. 3g, 3j, 3k S15, Table S6, Data S2 |
|  | test/Positive/F13 day1 | F13 day1_004.dat | PCR positive saliva for test | Figs. 3g, 3j, 3k S15, Table S6, Data S2 |
|  | test/Positive/F5 day3 | F5 day3_002.dat  F5 day3_006.dat | PCR positive saliva for test | Figs. 3g, 3j, 3k S15, Table S6, Data S2 |
|  | test/Positive/F5 day7 | F5 day7_001.dat  F5 day7_003.dat  F5 day7_004.dat  F5 day7_005.dat  F5 day7_006.dat  F5 day7_007.dat  F5 day7_008.dat  F5 day7_009.dat | PCR positive saliva for test | Figs. 3g, 3j, 3k S15, Table S6, Data S2 |
|  | test/Positive/F7 day14 | F7 day14_004.dat  F7 day14_006.dat | PCR positive saliva for test | Figs. 3g, 3j, 3k S15, Table S6, Data S2 |
|  | training/Negative/HD-112720-18 | HD-112720-18_003.dat | PCR negative saliva for training | Figs. 3a-3c, 3f, 3h, 3i, S13, S14, Table S5, Data S1 |
|  | training/Negative/HD-113020-35 | HD-113020-35_003.dat | PCR negative saliva for training | Figs. 3a-3c, 3f, 3h, 3i, S13, S14, Table S5, Data S1 |
|  | training/Positive/AS-2-2-bias+01_BK-985_045fil_TI_1st | AS-2-2-bias+01_BK-985_045fil_TI_1st_002.dat | PCR positive saliva for training | Figs. 3a-3c, 3f, 3h, 3i, S13, S14, Table S5, Data S1 |
|  | training/Positive/F1 day5 | F1 day5_006.dat | PCR positive saliva for training | Figs. 3a-3c, 3f, 3h, 3i, S13, S14, Table S5, Data S1 |
|  | training/Positive/F4 day7 | F4 day7_001.dat  F4 day7_003.dat  F4 day7_005.dat | PCR positive saliva for training | Figs. 3a-3c, 3f, 3h, 3i, S13, S14, Table S5, Data S1 |
|  | training/Positive/F6 day3 | F6 day3_009.dat | PCR positive saliva for training | Figs. 3a-3c, 3f, 3h, 3i, S13, S14, Table S5, Data S1 |
| Saliva(n=100 HO)_I-t data-20210209T093219Z-019.zip | test/Negative/HD-120220-58 | HD-120220-58_005.dat | PCR negative saliva for test | Figs. 3g, 3j, 3k S15, Table S6, Data S2 |
|  | test/Negative/HD-120220-6 | HD-120220-6_005.dat | PCR negative saliva for test | Figs. 3g, 3j, 3k S15, Table S6, Data S2 |
|  | test/Negative/HD-120420-43 | HD-120420-43_004.dat | PCR negative saliva for test | Figs. 3g, 3j, 3k S15, Table S6, Data S2 |
|  | test/Positive/AS-2-2-bias+01_BK-1091_045fil_TI | AS-2-2-bias+01_BK-1091_045fil_TI_009.dat | PCR positive saliva for test | Figs. 3g, 3j, 3k S15, Table S6, Data S2 |
|  | test/Positive/F13 day3 | F13 day3_002.dat | PCR positive saliva for test | Figs. 3g, 3j, 3k S15, Table S6, Data S2 |
|  | test/Positive/F14 day1 | F14 day1_007.dat | PCR positive saliva for test | Figs. 3g, 3j, 3k S15, Table S6, Data S2 |
|  | test/Positive/F17 day1 | F17 day1_002.dat  F17 day1_003.dat  F17 day1_004.dat  F17 day1_007.dat  F17 day1_008.dat | PCR positive saliva for test | Figs. 3g, 3j, 3k S15, Table S6, Data S2 |
|  | test/Positive/F3 day5 | F3 day5_001.dat | PCR positive saliva for test | Figs. 3g, 3j, 3k S15, Table S6, Data S2 |
|  | test/Positive/F5 day14 | F5 day14_003.dat | PCR positive saliva for test | Figs. 3g, 3j, 3k S15, Table S6, Data S2 |
|  | test/Positive/F9 day1 | F9 day1_002.dat  F9 day1_004.dat  F9 day1_006.dat | PCR positive saliva for test | Figs. 3g, 3j, 3k S15, Table S6, Data S2 |
|  | training/Negative/HD-113020-43 | HD-113020-43_001.dat  HD-113020-43_003.dat  HD-113020-43_004.dat | PCR negative saliva for training | Figs. 3a-3c, 3f, 3h, 3i, S13, S14, Table S5, Data S1 |
|  | training/Positive/AS-2-2-bias+01_BK-1124_045fil_TI | AS-2-2-bias+01_BK-1124_045fil_TI_004.dat | PCR positive saliva for training | Figs. 3a-3c, 3f, 3h, 3i, S13, S14, Table S5, Data S1 |
|  | training/Positive/AS-2-2-bias+01_BK-989_045fil_TI_1st | AS-2-2-bias+01_BK-989_045fil_TI_1st_005.dat  AS-2-2-bias+01_BK-989_045fil_TI_1st_008.dat  AS-2-2-bias+01_BK-989_045fil_TI_1st_009.dat | PCR positive saliva for training | Figs. 3a-3c, 3f, 3h, 3i, S13, S14, Table S5, Data S1 |
|  | training/Positive/F2 day7 | F2 day7_003.dat | PCR positive saliva for training | Figs. 3a-3c, 3f, 3h, 3i, S13, S14, Table S5, Data S1 |
|  | training/Positive/F6 day1 | F6 day1_008.dat | PCR positive saliva for training | Figs. 3a-3c, 3f, 3h, 3i, S13, S14, Table S5, Data S1 |
|  | training/Positive/F6 day3 | F6 day3_008.dat | PCR positive saliva for training | Figs. 3a-3c, 3f, 3h, 3i, S13, S14, Table S5, Data S1 |
| Saliva(n=100 HO)_I-t data-20210209T093219Z-020.zip | test/Negative/HD-112720-12 | HD-112720-12_005.dat | PCR negative saliva for test | Figs. 3g, 3j, 3k S15, Table S6, Data S2 |
|  | test/Negative/HD-120220-11 | HD-120220-11_005.dat | PCR negative saliva for test | Figs. 3g, 3j, 3k S15, Table S6, Data S2 |
|  | test/Negative/HD-120220-29 | HD-120220-29_005.dat | PCR negative saliva for test | Figs. 3g, 3j, 3k S15, Table S6, Data S2 |
|  | test/Negative/HD-120420-22 | HD-120420-22_004.dat | PCR negative saliva for test | Figs. 3g, 3j, 3k S15, Table S6, Data S2 |
|  | test/Negative/HD-120420-43 | HD-120420-43_002.dat  HD-120420-43_003.dat | PCR negative saliva for test | Figs. 3g, 3j, 3k S15, Table S6, Data S2 |
|  | test/Positive/AS-2-2-bias+01_BK-1154_045fil_TI | AS-2-2-bias+01_BK-1154_045fil_TI_001.dat | PCR positive saliva for test | Figs. 3g, 3j, 3k S15, Table S6, Data S2 |
|  | test/Positive/AS-2-2-bias+01_BK-965_045fil_TI_1st | AS-2-2-bias+01_BK-965_045fil_TI_1st_004.dat | PCR positive saliva for test | Figs. 3g, 3j, 3k S15, Table S6, Data S2 |
|  | test/Positive/AS-2-2-bias+01_BK-987_045fil_TI_1st | AS-2-2-bias+01_BK-987_045fil_TI_1st_001.dat | PCR positive saliva for test | Figs. 3g, 3j, 3k S15, Table S6, Data S2 |
|  | test/Positive/F7 day14 | F7 day14_007.dat | PCR positive saliva for test | Figs. 3g, 3j, 3k S15, Table S6, Data S2 |
|  | test/Positive/F9 day1 | F9 day1_003.dat | PCR positive saliva for test | Figs. 3g, 3j, 3k S15, Table S6, Data S2 |
|  | training/Positive/AS-2-2-bias+01_BK-1099_045fil_TI | AS-2-2-bias+01_BK-1099_045fil_TI_006.dat | PCR positive saliva for training | Figs. 3a-3c, 3f, 3h, 3i, S13, S14, Table S5, Data S1 |
|  | training/Positive/AS-2-2-bias+01_BK-1117_045fil_TI | AS-2-2-bias+01_BK-1117_045fil_TI_002.dat  AS-2-2-bias+01_BK-1117_045fil_TI_006.dat  AS-2-2-bias+01_BK-1117_045fil_TI_007.dat | PCR positive saliva for training | Figs. 3a-3c, 3f, 3h, 3i, S13, S14, Table S5, Data S1 |
|  | training/Positive/AS-2-2-bias+01_BK-948_045fil_TI_1st | AS-2-2-bias+01_BK-948_045fil_TI_1st_005.dat  AS-2-2-bias+01_BK-948_045fil_TI_1st_006.dat  AS-2-2-bias+01_BK-948_045fil_TI_1st_007.dat | PCR positive saliva for training | Figs. 3a-3c, 3f, 3h, 3i, S13, S14, Table S5, Data S1 |
|  | training/Positive/AS-2-2-bias+01_BK-989_045fil_TI_1st | AS-2-2-bias+01_BK-989_045fil_TI_1st_001.dat  AS-2-2-bias+01_BK-989_045fil_TI_1st_004.dat  AS-2-2-bias+01_BK-989_045fil_TI_1st_006.dat  AS-2-2-bias+01_BK-989_045fil_TI_1st_007.dat | PCR positive saliva for training | Figs. 3a-3c, 3f, 3h, 3i, S13, S14, Table S5, Data S1 |
|  | training/Positive/F2 day5 | F2 day5_009.dat | PCR positive saliva for training | Figs. 3a-3c, 3f, 3h, 3i, S13, S14, Table S5, Data S1 |
|  | training/Positive/F4 | F4_002.dat | PCR positive saliva for training | Figs. 3a-3c, 3f, 3h, 3i, S13, S14, Table S5, Data S1 |
|  | training/Positive/F5 day5 | F5 day5_007.dat | PCR positive saliva for training | Figs. 3a-3c, 3f, 3h, 3i, S13, S14, Table S5, Data S1 |
|  | training/Positive/F8 day3 | F8 day3_002.dat | PCR positive saliva for training | Figs. 3a-3c, 3f, 3h, 3i, S13, S14, Table S5, Data S1 |
| Saliva(n=100 HO)_I-t data-20210209T093219Z-021.zip | test/Negative/HD-120420-22 | HD-120420-22_001.dat  HD-120420-22_002.dat  HD-120420-22_003.dat | PCR negative saliva for test | Figs. 3g, 3j, 3k S15, Table S6, Data S2 |
|  | test/Negative/HD-120420-4 | HD-120420-4_001.dat  HD-120420-4_002.dat  HD-120420-4_003.dat  HD-120420-4_004.dat | PCR negative saliva for test | Figs. 3g, 3j, 3k S15, Table S6, Data S2 |
|  | test/Positive/AS-2-2-bias+01_BK-1151_045fil_TI | AS-2-2-bias+01_BK-1151_045fil_TI_003.dat | PCR positive saliva for test | Figs. 3g, 3j, 3k S15, Table S6, Data S2 |
|  | test/Positive/AS-2-2-bias+01_BK-984_045fil_TI_1st | AS-2-2-bias+01_BK-984_045fil_TI_1st_006.dat | PCR positive saliva for test | Figs. 3g, 3j, 3k S15, Table S6, Data S2 |
|  | test/Positive/F12 day1 | F12 day1_007.dat | PCR positive saliva for test | Figs. 3g, 3j, 3k S15, Table S6, Data S2 |
|  | test/Positive/F14 day1 | F14 day1_004.dat  F14 day1_008.dat | PCR positive saliva for test | Figs. 3g, 3j, 3k S15, Table S6, Data S2 |
|  | test/Positive/F9 day1 | F9 day1_001.dat | PCR positive saliva for test | Figs. 3g, 3j, 3k S15, Table S6, Data S2 |
|  | training/Positive/AS-2-2-bias+01_BK-1117_045fil_TI | AS-2-2-bias+01_BK-1117_045fil_TI_008.dat  AS-2-2-bias+01_BK-1117_045fil_TI_009.dat | PCR positive saliva for training | Figs. 3a-3c, 3f, 3h, 3i, S13, S14, Table S5, Data S1 |
|  | training/Positive/AS-2-2-bias+01_BK-959_045fil_TI_1st | AS-2-2-bias+01_BK-959_045fil_TI_1st_001.dat | PCR positive saliva for training | Figs. 3a-3c, 3f, 3h, 3i, S13, S14, Table S5, Data S1 |
|  | training/Positive/AS-2-2-bias+01_BK-973_045fil_TI_1st | AS-2-2-bias+01_BK-973_045fil_TI_1st_003.dat  AS-2-2-bias+01_BK-973_045fil_TI_1st_008.dat  AS-2-2-bias+01_BK-973_045fil_TI_1st_009.dat | PCR positive saliva for training | Figs. 3a-3c, 3f, 3h, 3i, S13, S14, Table S5, Data S1 |
|  | training/Positive/AS-2-2-bias+01_BK-992_045fil_TI_1st | AS-2-2-bias+01_BK-992_045fil_TI_1st_005.dat | PCR positive saliva for training | Figs. 3a-3c, 3f, 3h, 3i, S13, S14, Table S5, Data S1 |
|  | training/Positive/F1 day5 | F1 day5_009.dat | PCR positive saliva for training | Figs. 3a-3c, 3f, 3h, 3i, S13, S14, Table S5, Data S1 |
|  | training/Positive/F2 day3 | F2 day3_002.dat  F2 day3_006.dat | PCR positive saliva for training | Figs. 3a-3c, 3f, 3h, 3i, S13, S14, Table S5, Data S1 |
| Saliva(n=100 HO)_I-t data-20210209T093219Z-022.zip | test/Positive/AS-2-2-bias+01_BK-1154_045fil_TI | AS-2-2-bias+01_BK-1154_045fil_TI_009.dat | PCR positive saliva for test | Figs. 3g, 3j, 3k S15, Table S6, Data S2 |
|  | test/Positive/F10 day1① | F10 day1①_003.dat | PCR positive saliva for test | Figs. 3g, 3j, 3k S15, Table S6, Data S2 |
|  | test/Positive/F2 day14 | F2 day14_005.dat | PCR positive saliva for test | Figs. 3g, 3j, 3k S15, Table S6, Data S2 |
|  | test/Positive/F3 day3 | F3 day3_002.dat  F3 day3_003.dat  F3 day3_005.dat  F3 day3_006.dat  F3 day3_008.dat | PCR positive saliva for test | Figs. 3g, 3j, 3k S15, Table S6, Data S2 |
|  | training/Negative/HD-112720-10 | HD-112720-10_003.dat | PCR negative saliva for training | Figs. 3a-3c, 3f, 3h, 3i, S13, S14, Table S5, Data S1 |
|  | training/Positive/AS-2-2-bias+01_BK-1117_045fil_TI | AS-2-2-bias+01_BK-1117_045fil_TI_003.dat  AS-2-2-bias+01_BK-1117_045fil_TI_004.dat | PCR positive saliva for training | Figs. 3a-3c, 3f, 3h, 3i, S13, S14, Table S5, Data S1 |
|  | training/Positive/AS-2-2-bias+01_BK-948_045fil_TI_1st | AS-2-2-bias+01_BK-948_045fil_TI_1st_008.dat | PCR positive saliva for training | Figs. 3a-3c, 3f, 3h, 3i, S13, S14, Table S5, Data S1 |
|  | training/Positive/AS-2-2-bias+01_BK-973_045fil_TI_1st | AS-2-2-bias+01_BK-973_045fil_TI_1st_001.dat  AS-2-2-bias+01_BK-973_045fil_TI_1st_002.dat  AS-2-2-bias+01_BK-973_045fil_TI_1st_005.dat | PCR positive saliva for training | Figs. 3a-3c, 3f, 3h, 3i, S13, S14, Table S5, Data S1 |
|  | training/Positive/AS-2-2-bias+01_BK-985_045fil_TI_1st | AS-2-2-bias+01_BK-985_045fil_TI_1st_001.dat | PCR positive saliva for training | Figs. 3a-3c, 3f, 3h, 3i, S13, S14, Table S5, Data S1 |
|  | training/Positive/F2 day3 | F2 day3_003.dat  F2 day3_004.dat  F2 day3_005.dat  F2 day3_009.dat | PCR positive saliva for training | Figs. 3a-3c, 3f, 3h, 3i, S13, S14, Table S5, Data S1 |
|  | training/Positive/F8 day3 | F8 day3_006.dat | PCR positive saliva for training | Figs. 3a-3c, 3f, 3h, 3i, S13, S14, Table S5, Data S1 |
| Saliva(n=100 HO)_I-t data-20210209T093219Z-023.zip | test/Negative/HD-112720-25 | HD-112720-25_003.dat | PCR negative saliva for test | Figs. 3g, 3j, 3k S15, Table S6, Data S2 |
|  | test/Negative/HD-120420-21 | HD-120420-21_005.dat | PCR negative saliva for test | Figs. 3g, 3j, 3k S15, Table S6, Data S2 |
|  | test/Negative/HD-120420-29 | HD-120420-29_005.dat | PCR negative saliva for test | Figs. 3g, 3j, 3k S15, Table S6, Data S2 |
|  | test/Positive/AS-2-2-bias+01_BK-987_045fil_TI_1st | AS-2-2-bias+01_BK-987_045fil_TI_1st_007.dat | PCR positive saliva for test | Figs. 3g, 3j, 3k S15, Table S6, Data S2 |
|  | test/Positive/F1 day7 | F1 day7_007.dat | PCR positive saliva for test | Figs. 3g, 3j, 3k S15, Table S6, Data S2 |
|  | test/Positive/F13 day1 | F13 day1_002.dat  F13 day1_003.dat  F13 day1_005.dat  F13 day1_006.dat  F13 day1_007.dat  F13 day1_008.dat | PCR positive saliva for test | Figs. 3g, 3j, 3k S15, Table S6, Data S2 |
|  | training/Negative/HD-112720-36 | HD-112720-36_002.dat | PCR negative saliva for training | Figs. 3a-3c, 3f, 3h, 3i, S13, S14, Table S5, Data S1 |
|  | training/Negative/HD-113020-24 | HD-113020-24_005.dat | PCR negative saliva for training | Figs. 3a-3c, 3f, 3h, 3i, S13, S14, Table S5, Data S1 |
|  | training/Negative/HD-113020-28 | HD-113020-28_001.dat  HD-113020-28_003.dat | PCR negative saliva for training | Figs. 3a-3c, 3f, 3h, 3i, S13, S14, Table S5, Data S1 |
|  | training/Negative/HD-113020-44 | HD-113020-44_001.dat  HD-113020-44_002.dat  HD-113020-44_004.dat | PCR negative saliva for training | Figs. 3a-3c, 3f, 3h, 3i, S13, S14, Table S5, Data S1 |
|  | training/Positive/AS-2-2-bias+01_BK-1100_045fil_TI | AS-2-2-bias+01_BK-1100_045fil_TI_005.dat | PCR positive saliva for training | Figs. 3a-3c, 3f, 3h, 3i, S13, S14, Table S5, Data S1 |
|  | training/Positive/AS-2-2-bias+01_BK-1124_045fil_TI | AS-2-2-bias+01_BK-1124_045fil_TI_002.dat | PCR positive saliva for training | Figs. 3a-3c, 3f, 3h, 3i, S13, S14, Table S5, Data S1 |
|  | training/Positive/AS-2-2-bias+01_BK-1126_045fil_TI | AS-2-2-bias+01_BK-1126_045fil_TI_002.dat | PCR positive saliva for training | Figs. 3a-3c, 3f, 3h, 3i, S13, S14, Table S5, Data S1 |
|  | training/Positive/F1 day5 | F1 day5_004.dat | PCR positive saliva for training | Figs. 3a-3c, 3f, 3h, 3i, S13, S14, Table S5, Data S1 |
|  | training/Positive/F2 day7 | F2 day7_005.dat  F2 day7_007.dat | PCR positive saliva for training | Figs. 3a-3c, 3f, 3h, 3i, S13, S14, Table S5, Data S1 |
| Saliva(n=100 HO)_I-t data-20210209T093219Z-024.zip | test/Negative/HD-112720-25 | HD-112720-25_001.dat  HD-112720-25_002.dat  HD-112720-25_004.dat | PCR negative saliva for test | Figs. 3g, 3j, 3k S15, Table S6, Data S2 |
|  | test/Negative/HD-120420-13 | HD-120420-13_002.dat  HD-120420-13_003.dat  HD-120420-13_004.dat | PCR negative saliva for test | Figs. 3g, 3j, 3k S15, Table S6, Data S2 |
|  | test/Negative/HD-120420-3 | HD-120420-3_001.dat  HD-120420-3_002.dat  HD-120420-3_003.dat | PCR negative saliva for test | Figs. 3g, 3j, 3k S15, Table S6, Data S2 |
|  | test/Negative/HD-120420-36 | HD-120420-36_005.dat | PCR negative saliva for test | Figs. 3g, 3j, 3k S15, Table S6, Data S2 |
|  | test/Negative/HD-120720-4 | HD-120720-4_004.dat | PCR negative saliva for test | Figs. 3g, 3j, 3k S15, Table S6, Data S2 |
|  | test/Positive/AS-2-2-bias+01_BK-990_045fil_TI_1st | AS-2-2-bias+01_BK-990_045fil_TI_1st_002.dat | PCR positive saliva for test | Figs. 3g, 3j, 3k S15, Table S6, Data S2 |
|  | test/Positive/F1 day7 | F1 day7_009.dat | PCR positive saliva for test | Figs. 3g, 3j, 3k S15, Table S6, Data S2 |
|  | test/Positive/F9 day3 | F9 day3_004.dat | PCR positive saliva for test | Figs. 3g, 3j, 3k S15, Table S6, Data S2 |
|  | training/Negative/HD-112720-10 | HD-112720-10_004.dat | PCR negative saliva for training | Figs. 3a-3c, 3f, 3h, 3i, S13, S14, Table S5, Data S1 |
|  | training/Positive/AS-2-2-bias+01_BK-1099_045fil_TI | AS-2-2-bias+01_BK-1099_045fil_TI_004.dat  AS-2-2-bias+01_BK-1099_045fil_TI_005.dat  AS-2-2-bias+01_BK-1099_045fil_TI_008.dat | PCR positive saliva for training | Figs. 3a-3c, 3f, 3h, 3i, S13, S14, Table S5, Data S1 |
|  | training/Positive/AS-2-2-bias+01_BK-960_045fil_TI_1st | AS-2-2-bias+01_BK-960_045fil_TI_1st_002.dat | PCR positive saliva for training | Figs. 3a-3c, 3f, 3h, 3i, S13, S14, Table S5, Data S1 |
|  | training/Positive/F2 day7 | F2 day7_004.dat  F2 day7_008.dat | PCR positive saliva for training | Figs. 3a-3c, 3f, 3h, 3i, S13, S14, Table S5, Data S1 |
| Saliva(n=100 HO)_I-t data-20210209T093219Z-025.zip | test/Negative/HD-112720-35 | HD-112720-35_002.dat  HD-112720-35_003.dat  HD-112720-35_004.dat | PCR negative saliva for test | Figs. 3g, 3j, 3k S15, Table S6, Data S2 |
|  | test/Negative/HD-120420-21 | HD-120420-21_003.dat | PCR negative saliva for test | Figs. 3g, 3j, 3k S15, Table S6, Data S2 |
|  | test/Negative/HD-120720-4 | HD-120720-4_001.dat  HD-120720-4_003.dat | PCR negative saliva for test | Figs. 3g, 3j, 3k S15, Table S6, Data S2 |
|  | test/Positive/F13 day3 | F13 day3_004.dat  F13 day3_005.dat  F13 day3_008.dat  F13 day3_009.dat | PCR positive saliva for test | Figs. 3g, 3j, 3k S15, Table S6, Data S2 |
|  | training/Negative/HD-112720-36 | HD-112720-36_005.dat | PCR negative saliva for training | Figs. 3a-3c, 3f, 3h, 3i, S13, S14, Table S5, Data S1 |
|  | training/Positive/AS-2-2-bias+01_BK-1099_045fil_TI | AS-2-2-bias+01_BK-1099_045fil_TI_002.dat  AS-2-2-bias+01_BK-1099_045fil_TI_009.dat | PCR positive saliva for training | Figs. 3a-3c, 3f, 3h, 3i, S13, S14, Table S5, Data S1 |
|  | training/Positive/AS-2-2-bias+01_BK-959_045fil_TI_1st | AS-2-2-bias+01_BK-959_045fil_TI_1st_005.dat  AS-2-2-bias+01_BK-959_045fil_TI_1st_006.dat  AS-2-2-bias+01_BK-959_045fil_TI_1st_007.dat  AS-2-2-bias+01_BK-959_045fil_TI_1st_009.dat | PCR positive saliva for training | Figs. 3a-3c, 3f, 3h, 3i, S13, S14, Table S5, Data S1 |
|  | training/Positive/AS-2-2-bias+01_BK-992_045fil_TI_1st | AS-2-2-bias+01_BK-992_045fil_TI_1st_004.dat | PCR positive saliva for training | Figs. 3a-3c, 3f, 3h, 3i, S13, S14, Table S5, Data S1 |
|  | training/Positive/F5 day5 | F5 day5_006.dat | PCR positive saliva for training | Figs. 3a-3c, 3f, 3h, 3i, S13, S14, Table S5, Data S1 |
|  | training/Positive/F6 day1 | F6 day1_005.dat | PCR positive saliva for training | Figs. 3a-3c, 3f, 3h, 3i, S13, S14, Table S5, Data S1 |
| Saliva(n=100 HO)_I-t data-20210209T093219Z-026.zip | test/Negative/HD-112720-17 | HD-112720-17_002.dat  HD-112720-17_003.dat  HD-112720-17_004.dat | PCR negative saliva for test | Figs. 3g, 3j, 3k S15, Table S6, Data S2 |
|  | test/Negative/HD-120420-8 | HD-120420-8_005.dat | PCR negative saliva for test | Figs. 3g, 3j, 3k S15, Table S6, Data S2 |
|  | test/Positive/AS-2-2-bias+01_BK-965_045fil_TI_1st | AS-2-2-bias+01_BK-965_045fil_TI_1st_006.dat | PCR positive saliva for test | Figs. 3g, 3j, 3k S15, Table S6, Data S2 |
|  | test/Positive/AS-2-2-bias+01_BK-984_045fil_TI_1st | AS-2-2-bias+01_BK-984_045fil_TI_1st_009.dat | PCR positive saliva for test | Figs. 3g, 3j, 3k S15, Table S6, Data S2 |
|  | test/Positive/F10 day1① | F10 day1①_009.dat | PCR positive saliva for test | Figs. 3g, 3j, 3k S15, Table S6, Data S2 |
|  | test/Positive/F10 day1② | F10 day1②_009.dat | PCR positive saliva for test | Figs. 3g, 3j, 3k S15, Table S6, Data S2 |
|  | test/Positive/F13 day3 | F13 day3_001.dat  F13 day3_003.dat | PCR positive saliva for test | Figs. 3g, 3j, 3k S15, Table S6, Data S2 |
|  | training/Negative/HD-112720-18 | HD-112720-18_001.dat  HD-112720-18_002.dat | PCR negative saliva for training | Figs. 3a-3c, 3f, 3h, 3i, S13, S14, Table S5, Data S1 |
|  | training/Positive/AS-2-2-bias+01_BK-1146_045fil_TI | AS-2-2-bias+01_BK-1146_045fil_TI_001.dat | PCR positive saliva for training | Figs. 3a-3c, 3f, 3h, 3i, S13, S14, Table S5, Data S1 |
|  | training/Positive/AS-2-2-bias+01_BK-959_045fil_TI_1st | AS-2-2-bias+01_BK-959_045fil_TI_1st_002.dat  AS-2-2-bias+01_BK-959_045fil_TI_1st_003.dat | PCR positive saliva for training | Figs. 3a-3c, 3f, 3h, 3i, S13, S14, Table S5, Data S1 |
|  | training/Positive/F4 | F4_004.dat | PCR positive saliva for training | Figs. 3a-3c, 3f, 3h, 3i, S13, S14, Table S5, Data S1 |
|  | training/Positive/F5 day5 | F5 day5_003.dat  F5 day5_004.dat  F5 day5_008.dat | PCR positive saliva for training | Figs. 3a-3c, 3f, 3h, 3i, S13, S14, Table S5, Data S1 |
| Saliva(n=100 HO)_I-t data-20210209T093219Z-027.zip | test/Positive/AS-2-2-bias+01_BK-965_045fil_TI_1st | AS-2-2-bias+01_BK-965_045fil_TI_1st_001.dat  AS-2-2-bias+01_BK-965_045fil_TI_1st_002.dat  AS-2-2-bias+01_BK-965_045fil_TI_1st_005.dat | PCR positive saliva for test | Figs. 3g, 3j, 3k S15, Table S6, Data S2 |
|  | test/Positive/AS-2-2-bias+01_BK-984_045fil_TI_1st | AS-2-2-bias+01_BK-984_045fil_TI_1st_002.dat  AS-2-2-bias+01_BK-984_045fil_TI_1st_004.dat  AS-2-2-bias+01_BK-984_045fil_TI_1st_005.dat  AS-2-2-bias+01_BK-984_045fil_TI_1st_007.dat  AS-2-2-bias+01_BK-984_045fil_TI_1st_008.dat | PCR positive saliva for test | Figs. 3g, 3j, 3k S15, Table S6, Data S2 |
|  | test/Positive/F10 day1① | F10 day1①_001.dat  F10 day1①_004.dat  F10 day1①_006.dat  F10 day1①_007.dat | PCR positive saliva for test | Figs. 3g, 3j, 3k S15, Table S6, Data S2 |
|  | training/Positive/AS-2-2-bias+01_BK-1100_045fil_TI | AS-2-2-bias+01_BK-1100_045fil_TI_003.dat | PCR positive saliva for training | Figs. 3a-3c, 3f, 3h, 3i, S13, S14, Table S5, Data S1 |
|  | training/Positive/AS-2-2-bias+01_BK-1153_045fil_TI | AS-2-2-bias+01_BK-1153_045fil_TI_002.dat | PCR positive saliva for training | Figs. 3a-3c, 3f, 3h, 3i, S13, S14, Table S5, Data S1 |
|  | training/Positive/F7 day3 | F7 day3_006.dat  F7 day3_007.dat  F7 day3_009.dat | PCR positive saliva for training | Figs. 3a-3c, 3f, 3h, 3i, S13, S14, Table S5, Data S1 |
| Saliva(n=100 HO)_I-t data-20210209T093219Z-028.zip | test/Positive/AS-2-2-bias+01_BK-984_045fil_TI_1st | AS-2-2-bias+01_BK-984_045fil_TI_1st_001.dat | PCR positive saliva for test | Figs. 3g, 3j, 3k S15, Table S6, Data S2 |
|  | test/Positive/F10 day1② | F10 day1②_001.dat  F10 day1②_003.dat  F10 day1②_004.dat  F10 day1②_005.dat  F10 day1②_006.dat  F10 day1②_007.dat  F10 day1②_008.dat | PCR positive saliva for test | Figs. 3g, 3j, 3k S15, Table S6, Data S2 |
|  | training/Negative/HD-120220-4 | HD-120220-4_002.dat | PCR negative saliva for training | Figs. 3a-3c, 3f, 3h, 3i, S13, S14, Table S5, Data S1 |
|  | training/Positive/F6 day3 | F6 day3_005.dat  F6 day3_006.dat  F6 day3_007.dat | PCR positive saliva for training | Figs. 3a-3c, 3f, 3h, 3i, S13, S14, Table S5, Data S1 |
|  | training/Positive/F7 day3 | F7 day3_003.dat  F7 day3_004.dat  F7 day3_005.dat | PCR positive saliva for training | Figs. 3a-3c, 3f, 3h, 3i, S13, S14, Table S5, Data S1 |
| Saliva(n=100 HO)_I-t data-20210209T093219Z-029.zip | test/Positive/AS-2-2-bias+01_BK-969_045fil_TI_1st | AS-2-2-bias+01_BK-969_045fil_TI_1st_007.dat  AS-2-2-bias+01_BK-969_045fil_TI_1st_008.dat | PCR positive saliva for test | Figs. 3g, 3j, 3k S15, Table S6, Data S2 |
|  | training/Negative/HD-120220-4 | HD-120220-4_001.dat  HD-120220-4_003.dat  HD-120220-4_004.dat | PCR negative saliva for training | Figs. 3a-3c, 3f, 3h, 3i, S13, S14, Table S5, Data S1 |
|  | training/Positive/F5 day1 | F5 day1_005.dat  F5 day1_006.dat  F5 day1_008.dat  F5 day1_009.dat | PCR positive saliva for training | Figs. 3a-3c, 3f, 3h, 3i, S13, S14, Table S5, Data S1 |
|  | training/Positive/F6 day3 | F6 day3_001.dat  F6 day3_002.dat  F6 day3_004.dat | PCR positive saliva for training | Figs. 3a-3c, 3f, 3h, 3i, S13, S14, Table S5, Data S1 |
|  | training/Positive/F7 day3 | F7 day3_001.dat  F7 day3_002.dat  F7 day3_008.dat | PCR positive saliva for training | Figs. 3a-3c, 3f, 3h, 3i, S13, S14, Table S5, Data S1 |
| Saliva(n=100 HO)_I-t data-20210209T093219Z-030.zip | test/Positive/AS-2-2-bias+01_BK-969_045fil_TI_1st | AS-2-2-bias+01_BK-969_045fil_TI_1st_001.dat  AS-2-2-bias+01_BK-969_045fil_TI_1st_002.dat  AS-2-2-bias+01_BK-969_045fil_TI_1st_003.dat  AS-2-2-bias+01_BK-969_045fil_TI_1st_004.dat  AS-2-2-bias+01_BK-969_045fil_TI_1st_005.dat  AS-2-2-bias+01_BK-969_045fil_TI_1st_006.dat | PCR positive saliva for test | Figs. 3g, 3j, 3k S15, Table S6, Data S2 |
|  | test/Positive/AS-2-2-bias+01_BK-987_045fil_TI_1st | AS-2-2-bias+01_BK-987_045fil_TI_1st_008.dat | PCR positive saliva for test | Figs. 3g, 3j, 3k S15, Table S6, Data S2 |
|  | test/Positive/F12 day1 | F12 day1_005.dat  F12 day1_006.dat  F12 day1_008.dat  F12 day1_009.dat | PCR positive saliva for test | Figs. 3g, 3j, 3k S15, Table S6, Data S2 |
|  | test/Positive/F14 day3 | F14 day3_009.dat | PCR positive saliva for test | Figs. 3g, 3j, 3k S15, Table S6, Data S2 |
|  | training/Positive/AS-2-2-bias+01_BK-970_045fil_TI_1st | AS-2-2-bias+01_BK-970_045fil_TI_1st_009.dat | PCR positive saliva for training | Figs. 3a-3c, 3f, 3h, 3i, S13, S14, Table S5, Data S1 |
|  | training/Positive/F5 day1 | F5 day1_001.dat  F5 day1_004.dat | PCR positive saliva for training | Figs. 3a-3c, 3f, 3h, 3i, S13, S14, Table S5, Data S1 |
|  | training/Positive/F6 day3 | F6 day3_003.dat | PCR positive saliva for training | Figs. 3a-3c, 3f, 3h, 3i, S13, S14, Table S5, Data S1 |
| Saliva(n=100 HO)_I-t data-20210209T093219Z-031.zip | test/Negative/HD-120220-6 | HD-120220-6_001.dat  HD-120220-6_002.dat  HD-120220-6_003.dat | PCR negative saliva for test | Figs. 3g, 3j, 3k S15, Table S6, Data S2 |
|  | test/Positive/AS-2-2-bias+01_BK-1154_045fil_TI | AS-2-2-bias+01_BK-1154_045fil_TI_007.dat  AS-2-2-bias+01_BK-1154_045fil_TI_008.dat | PCR positive saliva for test | Figs. 3g, 3j, 3k S15, Table S6, Data S2 |
|  | test/Positive/AS-2-2-bias+01_BK-987_045fil_TI_1st | AS-2-2-bias+01_BK-987_045fil_TI_1st_005.dat  AS-2-2-bias+01_BK-987_045fil_TI_1st_006.dat | PCR positive saliva for test | Figs. 3g, 3j, 3k S15, Table S6, Data S2 |
|  | test/Positive/F12 day1 | F12 day1_002.dat  F12 day1_003.dat  F12 day1_004.dat | PCR positive saliva for test | Figs. 3g, 3j, 3k S15, Table S6, Data S2 |
|  | test/Positive/F14 day3 | F14 day3_005.dat  F14 day3_007.dat  F14 day3_008.dat | PCR positive saliva for test | Figs. 3g, 3j, 3k S15, Table S6, Data S2 |
|  | training/Positive/AS-2-2-bias+01_BK-970_045fil_TI_1st | AS-2-2-bias+01_BK-970_045fil_TI_1st_008.dat | PCR positive saliva for training | Figs. 3a-3c, 3f, 3h, 3i, S13, S14, Table S5, Data S1 |
|  | training/Positive/F5 day1 | F5 day1_003.dat | PCR positive saliva for training | Figs. 3a-3c, 3f, 3h, 3i, S13, S14, Table S5, Data S1 |
| Saliva(n=100 HO)_I-t data-20210209T093219Z-032.zip | test/Negative/HD-120420-29 | HD-120420-29_004.dat | PCR negative saliva for test | Figs. 3g, 3j, 3k S15, Table S6, Data S2 |
|  | test/Positive/AS-2-2-bias+01_BK-1151_045fil_TI | AS-2-2-bias+01_BK-1151_045fil_TI_009.dat | PCR positive saliva for test | Figs. 3g, 3j, 3k S15, Table S6, Data S2 |
|  | test/Positive/AS-2-2-bias+01_BK-1154_045fil_TI | AS-2-2-bias+01_BK-1154_045fil_TI_004.dat  AS-2-2-bias+01_BK-1154_045fil_TI_005.dat | PCR positive saliva for test | Figs. 3g, 3j, 3k S15, Table S6, Data S2 |
|  | test/Positive/AS-2-2-bias+01_BK-987_045fil_TI_1st | AS-2-2-bias+01_BK-987_045fil_TI_1st_002.dat  AS-2-2-bias+01_BK-987_045fil_TI_1st_003.dat  AS-2-2-bias+01_BK-987_045fil_TI_1st_004.dat | PCR positive saliva for test | Figs. 3g, 3j, 3k S15, Table S6, Data S2 |
|  | test/Positive/F12 day1 | F12 day1_001.dat | PCR positive saliva for test | Figs. 3g, 3j, 3k S15, Table S6, Data S2 |
|  | test/Positive/F14 day3 | F14 day3_001.dat  F14 day3_002.dat  F14 day3_003.dat  F14 day3_004.dat  F14 day3_006.dat | PCR positive saliva for test | Figs. 3g, 3j, 3k S15, Table S6, Data S2 |
|  | test/Positive/F7 day1 | F7 day1_009.dat | PCR positive saliva for test | Figs. 3g, 3j, 3k S15, Table S6, Data S2 |
|  | training/Positive/AS-2-2-bias+01_BK-970_045fil_TI_1st | AS-2-2-bias+01_BK-970_045fil_TI_1st_007.dat | PCR positive saliva for training | Figs. 3a-3c, 3f, 3h, 3i, S13, S14, Table S5, Data S1 |
|  | training/Positive/F6 day5 | F6 day5_009.dat | PCR positive saliva for training | Figs. 3a-3c, 3f, 3h, 3i, S13, S14, Table S5, Data S1 |
| Saliva(n=100 HO)_I-t data-20210209T093219Z-033.zip | test/Negative/HD-120420-29 | HD-120420-29_002.dat  HD-120420-29_002.dat | PCR negative saliva for test | Figs. 3g, 3j, 3k S15, Table S6, Data S2 |
|  | test/Positive/AS-2-2-bias+01_BK-1091_045fil_T | AS-2-2-bias+01_BK-1091_045fil_TI_007.dat | PCR positive saliva for test | Figs. 3g, 3j, 3k S15, Table S6, Data S2 |
|  | test/Positive/AS-2-2-bias+01_BK-1151_045fil_TI | AS-2-2-bias+01_BK-1151_045fil_TI_007.dat  AS-2-2-bias+01_BK-1151_045fil_TI_008.dat | PCR positive saliva for test | Figs. 3g, 3j, 3k S15, Table S6, Data S2 |
|  | test/Positive/AS-2-2-bias+01_BK-1154_045fil_TI | AS-2-2-bias+01_BK-1154_045fil_TI_002.dat  AS-2-2-bias+01_BK-1154_045fil_TI_003.dat | PCR positive saliva for test | Figs. 3g, 3j, 3k S15, Table S6, Data S2 |
|  | test/Positive/AS-2-2-bias+01_BK-957_045fil_TI_1st | AS-2-2-bias+01_BK-957_045fil_TI_1st_009.dat | PCR positive saliva for test | Figs. 3g, 3j, 3k S15, Table S6, Data S2 |
|  | test/Positive/F10 day1② | F10 day1②_002.dat | PCR positive saliva for test | FFigs. 3g, 3j, 3k S15, Table S6, Data S2 |
|  | test/Positive/F7 day1 | F7 day1_006.dat | PCR positive saliva for test | Figs. 3g, 3j, 3k S15, Table S6, Data S2 |
|  | training/Negative/HD-113020-35 | HD-113020-35_004.dat | PCR negative saliva for training | Figs. 3a-3c, 3f, 3h, 3i, S13, S14, Table S5, Data S1 |
|  | training/Positive/AS-2-2-bias+01_BK-970_045fil_TI_1st | AS-2-2-bias+01_BK-970_045fil_TI_1st_003.dat  AS-2-2-bias+01_BK-970_045fil_TI_1st_004.dat  AS-2-2-bias+01_BK-970_045fil_TI_1st_005.dat  AS-2-2-bias+01_BK-970_045fil_TI_1st_006.dat | PCR positive saliva for training | Figs. 3a-3c, 3f, 3h, 3i, S13, S14, Table S5, Data S1 |
| Saliva(n=100 HO)_I-t data-20210209T093219Z-034.zip | test/Negative/HD-120420-29 | HD-120420-29_001.dat | PCR negative saliva for test | Figs. 3g, 3j, 3k S15, Table S6, Data S2 |
|  | test/Positive/AS-2-2-bias+01_BK-1091_045fil_TI | AS-2-2-bias+01_BK-1091_045fil_TI_005.dat  AS-2-2-bias+01_BK-1091_045fil_TI_006.dat  AS-2-2-bias+01_BK-1091_045fil_TI_008.dat | PCR positive saliva for test | Figs. 3g, 3j, 3k S15, Table S6, Data S2 |
|  | test/Positive/AS-2-2-bias+01_BK-1151_045fil_TI | AS-2-2-bias+01_BK-1151_045fil_TI_005.dat  AS-2-2-bias+01_BK-1151_045fil_TI_006.dat | PCR positive saliva for test | Figs. 3g, 3j, 3k S15, Table S6, Data S2 |
|  | test/Positive/AS-2-2-bias+01_BK-990_045fil_TI_1st | AS-2-2-bias+01_BK-990_045fil_TI_1st_009.dat | PCR positive saliva for test | Figs. 3g, 3j, 3k S15, Table S6, Data S2 |
|  | test/Positive/F7 day1 | F7 day1_004.dat  F7 day1_005.dat  F7 day1_007.dat | PCR positive saliva for test | Figs. 3g, 3j, 3k S15, Table S6, Data S2 |
|  | training/Negative/HD-113020-35 | HD-113020-35_001.dat  HD-113020-35_002.dat | PCR negative saliva for training | Figs. 3a-3c, 3f, 3h, 3i, S13, S14, Table S5, Data S1 |
|  | training/Positive/AS-2-2-bias+01_BK-1126_045fil_TI | AS-2-2-bias+01_BK-1126_045fil_TI_009.dat | PCR positive saliva for training | Figs. 3a-3c, 3f, 3h, 3i, S13, S14, Table S5, Data S1 |
|  | training/Positive/AS-2-2-bias+01_BK-953_045fil_TI_1st | AS-2-2-bias+01_BK-953_045fil_TI_1st_009.dat | PCR positive saliva for training | Figs. 3a-3c, 3f, 3h, 3i, S13, S14, Table S5, Data S1 |
|  | training/Positive/F6 day5 | F6 day5_007.dat  F6 day5_008.dat | PCR positive saliva for training | Figs. 3a-3c, 3f, 3h, 3i, S13, S14, Table S5, Data S1 |
| Saliva(n=100 HO)_I-t data-20210209T093219Z-035.zip | test/Negative/HD-120220-58 | HD-120220-58_002.dat  HD-120220-58_003.dat | PCR negative saliva for test | Figs. 3g, 3j, 3k S15, Table S6, Data S2 |
|  | test/Positive/AS-2-2-bias+01_BK-1091_045fil_TI | AS-2-2-bias+01_BK-1091_045fil_TI_002.dat  AS-2-2-bias+01_BK-1091_045fil_TI_003.dat  AS-2-2-bias+01_BK-1091_045fil_TI_004.dat | PCR positive saliva for test | Figs. 3g, 3j, 3k S15, Table S6, Data S2 |
|  | test/Positive/AS-2-2-bias+01_BK-1151_045fil_TI | AS-2-2-bias+01_BK-1151_045fil_TI_002.dat | PCR positive saliva for test | Figs. 3g, 3j, 3k S15, Table S6, Data S2 |
|  | test/Positive/AS-2-2-bias+01_BK-957_045fil_TI_1st | AS-2-2-bias+01_BK-957_045fil_TI_1st_006.dat  AS-2-2-bias+01_BK-957_045fil_TI_1st_007.dat  AS-2-2-bias+01_BK-957_045fil_TI_1st_008.dat | PCR positive saliva for test | Figs. 3g, 3j, 3k S15, Table S6, Data S2 |
|  | test/Positive/F7 day1 | F7 day1_001.dat  F7 day1_002.dat | PCR positive saliva for test | Figs. 3g, 3j, 3k S15, Table S6, Data S2 |
|  | training/Positive/AS-2-2-bias+01_BK-953_045fil_TI_1st | AS-2-2-bias+01_BK-953_045fil_TI_1st_008.dat | PCR positive saliva for training | Figs. 3a-3c, 3f, 3h, 3i, S13, S14, Table S5, Data S1 |
|  | training/Positive/F6 day5 | F6 day5_004.dat  F6 day5_005.dat  F6 day5_006.dat | PCR positive saliva for training | Figs. 3a-3c, 3f, 3h, 3i, S13, S14, Table S5, Data S1 |
| Saliva(n=100 HO)_I-t data-20210209T093219Z-036.zip | test/Negative/HD-120220-36 | HD-120220-36_001.dat  HD-120220-36_002.dat | PCR negative saliva for test | Figs. 3g, 3j, 3k S15, Table S6, Data S2 |
|  | test/Negative/HD-120220-58 | HD-120220-58_001.dat | PCR negative saliva for test | Figs. 3g, 3j, 3k S15, Table S6, Data S2 |
|  | test/Negative/HD-120720-33 | HD-120720-33_004.dat | PCR negative saliva for test | Figs. 3g, 3j, 3k S15, Table S6, Data S2 |
|  | test/Positive/AS-2-2-bias+01_BK-1091_045fil_TI | AS-2-2-bias+01_BK-1091_045fil_TI_001.dat | PCR positive saliva for test | Figs. 3g, 3j, 3k S15, Table S6, Data S2 |
|  | test/Positive/AS-2-2-bias+01_BK-1151_045fil_TI | AS-2-2-bias+01_BK-1151_045fil_TI_001.dat | PCR positive saliva for test | Figs. 3g, 3j, 3k S15, Table S6, Data S2 |
|  | test/Positive/AS-2-2-bias+01_BK-957_045fil_TI_1st | AS-2-2-bias+01_BK-957_045fil_TI_1st_004.dat  AS-2-2-bias+01_BK-957_045fil_TI_1st_005.dat | PCR positive saliva for test | Figs. 3g, 3j, 3k S15, Table S6, Data S2 |
|  | test/Positive/AS-2-2-bias+01_BK-990_045fil_TI_1st | AS-2-2-bias+01_BK-990_045fil_TI_1st_007.dat  AS-2-2-bias+01_BK-990_045fil_TI_1st_008.dat | PCR positive saliva for test | Figs. 3g, 3j, 3k S15, Table S6, Data S2 |
|  | test/Positive/F1 day7 | F1 day7_008.dat | PCR positive saliva for test | Figs. 3g, 3j, 3k S15, Table S6, Data S2 |
|  | training/Positive/AS-2-2-bias+01_BK-1126_045fil_TI | AS-2-2-bias+01_BK-1126_045fil_TI_008.dat | PCR positive saliva for training | Figs. 3a-3c, 3f, 3h, 3i, S13, S14, Table S5, Data S1 |
|  | training/Positive/AS-2-2-bias+01_BK-953_045fil_TI_1st | AS-2-2-bias+01_BK-953_045fil_TI_1st_005.dat  AS-2-2-bias+01_BK-953_045fil_TI_1st_007.dat | PCR positive saliva for training | Figs. 3a-3c, 3f, 3h, 3i, S13, S14, Table S5, Data S1 |
|  | training/Positive/F6 day5 | F6 day5_003.dat | PCR positive saliva for training | Figs. 3a-3c, 3f, 3h, 3i, S13, S14, Table S5, Data S1 |
| Saliva(n=100 HO)_I-t data-20210209T093219Z-037.zip | test/Negative/HD-120720-33 | HD-120720-33_002.dat  HD-120720-33_003.dat | PCR negative saliva for test | Figs. 3g, 3j, 3k S15, Table S6, Data S2 |
|  | test/Positive/AS-2-2-bias+01_BK-1095_045fil_TI | AS-2-2-bias+01_BK-1095_045fil_TI_009.dat | PCR positive saliva for test | Figs. 3g, 3j, 3k S15, Table S6, Data S2 |
|  | test/Positive/AS-2-2-bias+01_BK-1150_045fil_TI | AS-2-2-bias+01_BK-1150_045fil_TI_009.dat | PCR positive saliva for test | Figs. 3g, 3j, 3k S15, Table S6, Data S2 |
|  | test/Positive/AS-2-2-bias+01_BK-957_045fil_TI_1st | AS-2-2-bias+01_BK-957_045fil_TI_1st_002.dat  AS-2-2-bias+01_BK-957_045fil_TI_1st_003.dat | PCR positive saliva for test | Figs. 3g, 3j, 3k S15, Table S6, Data S2 |
|  | test/Positive/AS-2-2-bias+01_BK-990_045fil_TI_1st | AS-2-2-bias+01_BK-990_045fil_TI_1st_005.dat  AS-2-2-bias+01_BK-990_045fil_TI_1st_006.dat | PCR positive saliva for test | Figs. 3g, 3j, 3k S15, Table S6, Data S2 |
|  | test/Positive/F1 day7 | F1 day7_006.dat | PCR positive saliva for test | Figs. 3g, 3j, 3k S15, Table S6, Data S2 |
|  | training/Positive/AS-2-2-bias+01_BK-1126_045fil_TI | AS-2-2-bias+01_BK-1126_045fil_TI_005.dat  AS-2-2-bias+01_BK-1126_045fil_TI_006.dat  AS-2-2-bias+01_BK-1126_045fil_TI_007.dat | PCR positive saliva for training | Figs. 3a-3c, 3f, 3h, 3i, S13, S14, Table S5, Data S1 |
|  | training/Positive/AS-2-2-bias+01_BK-953_045fil_TI_1st | AS-2-2-bias+01_BK-953_045fil_TI_1st_004.dat  AS-2-2-bias+01_BK-953_045fil_TI_1st_006.dat | PCR positive saliva for training | Figs. 3a-3c, 3f, 3h, 3i, S13, S14, Table S5, Data S1 |
|  | training/Positive/F6 day5 | F6 day5_002.dat | PCR positive saliva for training | Figs. 3a-3c, 3f, 3h, 3i, S13, S14, Table S5, Data S1 |
|  | training/Positive/F8 day3 | F8 day3_009.dat | PCR positive saliva for training | Figs. 3a-3c, 3f, 3h, 3i, S13, S14, Table S5, Data S1 |
| Saliva(n=100 HO)_I-t data-20210209T093219Z-038.zip | test/Negative/HD-113020-33 | HD-113020-33_002.dat  HD-113020-33_003.dat  HD-113020-33_004.dat | PCR negative saliva for test | Figs. 3g, 3j, 3k S15, Table S6, Data S2 |
|  | test/Negative/HD-120720-33 | HD-120720-33_001.dat | PCR negative saliva for test | Figs. 3g, 3j, 3k S15, Table S6, Data S2 |
|  | test/Positive/AS-2-2-bias+01_BK-1095_045fil_TI | AS-2-2-bias+01_BK-1095_045fil_TI_008.dat | PCR positive saliva for test | Figs. 3g, 3j, 3k S15, Table S6, Data S2 |
|  | test/Positive/AS-2-2-bias+01_BK-1150_045fil_TI | AS-2-2-bias+01_BK-1150_045fil_TI_007.dat  AS-2-2-bias+01_BK-1150_045fil_TI_008.dat | PCR positive saliva for test | Figs. 3g, 3j, 3k S15, Table S6, Data S2 |
|  | test/Positive/AS-2-2-bias+01_BK-957_045fil_TI_1st | AS-2-2-bias+01_BK-957_045fil_TI_1st_001.dat | PCR positive saliva for test | Figs. 3g, 3j, 3k S15, Table S6, Data S2 |
|  | test/Positive/AS-2-2-bias+01_BK-990_045fil_TI_1st | AS-2-2-bias+01_BK-990_045fil_TI_1st_003.dat  AS-2-2-bias+01_BK-990_045fil_TI_1st_004.dat | PCR positive saliva for test | Figs. 3g, 3j, 3k S15, Table S6, Data S2 |
|  | test/Positive/F1 day7 | F1 day7_005.dat | PCR positive saliva for test | Figs. 3g, 3j, 3k S15, Table S6, Data S2 |
|  | test/Positive/F3 day5 | F3 day5_006.dat | PCR positive saliva for test | Figs. 3g, 3j, 3k S15, Table S6, Data S2 |
|  | training/Positive/AS-2-2-bias+01_BK-1126_045fil_TI | AS-2-2-bias+01_BK-1126_045fil_TI_004.dat | PCR positive saliva for training | Figs. 3a-3c, 3f, 3h, 3i, S13, S14, Table S5, Data S1 |
|  | training/Positive/AS-2-2-bias+01_BK-953_045fil_TI_1st | AS-2-2-bias+01_BK-953_045fil_TI_1st_002.dat | PCR positive saliva for training | Figs. 3a-3c, 3f, 3h, 3i, S13, S14, Table S5, Data S1 |
|  | training/Positive/F8 day3 | F8 day3_007.dat | PCR positive saliva for training | Figs. 3a-3c, 3f, 3h, 3i, S13, S14, Table S5, Data S1 |
| Saliva(n=100 HO)_I-t data-20210209T093219Z-039.zip | test/Negative/HD-112720-30 | HD-112720-30_003.dat  HD-112720-30_004.dat | PCR negative saliva for test | Figs. 3g, 3j, 3k S15, Table S6, Data S2 |
|  | test/Positive/AS-2-2-bias+01_BK-1095_045fil_TI | AS-2-2-bias+01_BK-1095_045fil_TI_006.dat  AS-2-2-bias+01_BK-1095_045fil_TI_007.dat | PCR positive saliva for test | Figs. 3g, 3j, 3k S15, Table S6, Data S2 |
|  | test/Positive/AS-2-2-bias+01_BK-1150_045fil_TI | AS-2-2-bias+01_BK-1150_045fil_TI_006.dat | PCR positive saliva for test | Figs. 3g, 3j, 3k S15, Table S6, Data S2 |
|  | test/Positive/AS-2-2-bias+01_BK-1151_045fil_TI | AS-2-2-bias+01_BK-1151_045fil_TI_004.dat | PCR positive saliva for test | Figs. 3g, 3j, 3k S15, Table S6, Data S2 |
|  | test/Positive/AS-2-2-bias+01_BK-967_045fil_TI_1st | AS-2-2-bias+01_BK-967_045fil_TI_1st_008.dat | PCR positive saliva for test | Figs. 3g, 3j, 3k S15, Table S6, Data S2 |
|  | test/Positive/AS-2-2-bias+01_BK-986_045fil_TI_1st | AS-2-2-bias+01_BK-986_045fil_TI_1st_009.dat | PCR positive saliva for test | Figs. 3g, 3j, 3k S15, Table S6, Data S2 |
|  | test/Positive/AS-2-2-bias+01_BK-990_045fil_TI_1st | AS-2-2-bias+01_BK-990_045fil_TI_1st_001.dat | PCR positive saliva for test | Figs. 3g, 3j, 3k S15, Table S6, Data S2 |
|  | test/Positive/F1 day7 | F1 day7_003.dat  F1 day7_004.dat | PCR positive saliva for test | Figs. 3g, 3j, 3k S15, Table S6, Data S2 |
|  | test/Positive/F3 day5 | F3 day5_005.dat | PCR positive saliva for test | Figs. 3g, 3j, 3k S15, Table S6, Data S2 |
|  | training/Positive/AS-2-2-bias+01_BK-1126_045fil_TI | AS-2-2-bias+01_BK-1126_045fil_TI_003.dat | PCR positive saliva for training | Figs. 3a-3c, 3f, 3h, 3i, S13, S14, Table S5, Data S1 |
|  | training/Positive/AS-2-2-bias+01_BK-953_045fil_TI_1st | AS-2-2-bias+01_BK-953_045fil_TI_1st_001.dat  AS-2-2-bias+01_BK-953_045fil_TI_1st_003.dat | PCR positive saliva for training | Figs. 3a-3c, 3f, 3h, 3i, S13, S14, Table S5, Data S1 |
| Saliva(n=100 HO)_I-t data-20210209T093219Z-040.zip | test/Negative/HD-112720-30 | HD-112720-30_002.dat | PCR negative saliva for test | Figs. 3g, 3j, 3k S15, Table S6, Data S2 |
|  | test/Negative/HD-113020-33 | HD-113020-33_001.dat | PCR negative saliva for test | Figs. 3g, 3j, 3k S15, Table S6, Data S2 |
|  | test/Positive/AS-2-2-bias+01_BK-1095_045fil_TI | AS-2-2-bias+01_BK-1095_045fil_TI_004.dat  AS-2-2-bias+01_BK-1095_045fil_TI_005.dat | PCR positive saliva for test | Figs. 3g, 3j, 3k S15, Table S6, Data S2 |
|  | test/Positive/AS-2-2-bias+01_BK-1105_045fil_TI | AS-2-2-bias+01_BK-1105_045fil_TI_009.dat | PCR positive saliva for test | Figs. 3g, 3j, 3k S15, Table S6, Data S2 |
|  | test/Positive/AS-2-2-bias+01_BK-1150_045fil_TI | AS-2-2-bias+01_BK-1150_045fil_TI_005.dat | PCR positive saliva for test | Figs. 3g, 3j, 3k S15, Table S6, Data S2 |
|  | test/Positive/AS-2-2-bias+01_BK-967_045fil_TI_1st | AS-2-2-bias+01_BK-967_045fil_TI_1st_006.dat  AS-2-2-bias+01_BK-967_045fil_TI_1st_007.dat | PCR positive saliva for test | Figs. 3g, 3j, 3k S15, Table S6, Data S2 |
|  | test/Positive/F1 day7 | F1 day7_001.dat  F1 day7_002.dat | PCR positive saliva for test | Figs. 3g, 3j, 3k S15, Table S6, Data S2 |
|  | test/Positive/F3 day5 | F3 day5_003.dat | PCR positive saliva for test | Figs. 3g, 3j, 3k S15, Table S6, Data S2 |
|  | training/Positive/AS-2-2-bias+01_BK-1126_045fil_TI | AS-2-2-bias+01_BK-1126_045fil_TI_001.dat | PCR positive saliva for training | Figs. 3a-3c, 3f, 3h, 3i, S13, S14, Table S5, Data S1 |
|  | training/Positive/F8 day3 | F8 day3_004.dat  F8 day3_005.dat  F8 day3_008.dat | PCR positive saliva for training | Figs. 3a-3c, 3f, 3h, 3i, S13, S14, Table S5, Data S1 |
| Saliva(n=100 HO)_I-t data-20210209T093219Z-041.zip | test/Positive/AS-2-2-bias+01_BK-1095_045fil_TI | AS-2-2-bias+01_BK-1095_045fil_TI_002.dat  AS-2-2-bias+01_BK-1095_045fil_TI_003.dat | PCR positive saliva for test | Figs. 3g, 3j, 3k S15, Table S6, Data S2 |
|  | test/Positive/AS-2-2-bias+01_BK-1103_045fil_TI | AS-2-2-bias+01_BK-1103_045fil_TI_009.dat | PCR positive saliva for test | Figs. 3g, 3j, 3k S15, Table S6, Data S2 |
|  | test/Positive/AS-2-2-bias+01_BK-1105_045fil_TI | AS-2-2-bias+01_BK-1105_045fil_TI_007.dat  AS-2-2-bias+01_BK-1105_045fil_TI_008.dat | PCR positive saliva for test | Figs. 3g, 3j, 3k S15, Table S6, Data S2 |
|  | test/Positive/AS-2-2-bias+01_BK-1150_045fil_TI | AS-2-2-bias+01_BK-1150_045fil_TI_001.dat  AS-2-2-bias+01_BK-1150_045fil_TI_002.dat  AS-2-2-bias+01_BK-1150_045fil_TI_003.dat  AS-2-2-bias+01_BK-1150_045fil_TI_004.dat | PCR positive saliva for test | Figs. 3g, 3j, 3k S15, Table S6, Data S2 |
|  | test/Positive/AS-2-2-bias+01_BK-967_045fil_TI_1st | AS-2-2-bias+01_BK-967_045fil_TI_1st_004.dat  AS-2-2-bias+01_BK-967_045fil_TI_1st_005.dat | PCR positive saliva for test | Figs. 3g, 3j, 3k S15, Table S6, Data S2 |
|  | test/Positive/AS-2-2-bias+01_BK-986_045fil_TI_1st | AS-2-2-bias+01_BK-986_045fil_TI_1st_007.dat  AS-2-2-bias+01_BK-986_045fil_TI_1st_008.dat | PCR positive saliva for test | Figs. 3g, 3j, 3k S15, Table S6, Data S2 |
|  | test/Positive/F3 day5 | F3 day5_002.dat | PCR positive saliva for test | Figs. 3g, 3j, 3k S15, Table S6, Data S2 |
|  | test/Positive/F7 day5 | F7 day5_002.dat | PCR positive saliva for test | Figs. 3g, 3j, 3k S15, Table S6, Data S2 |
| Saliva(n=100 HO)_I-t data-20210209T093219Z-042.zip | test/Positive/AS-2-2-bias+01_BK-1103_045fil_TI | AS-2-2-bias+01_BK-1103_045fil_TI_008.dat | PCR positive saliva for test | Figs. 3g, 3j, 3k S15, Table S6, Data S2 |
|  | test/Positive/AS-2-2-bias+01_BK-1105_045fil_TI | AS-2-2-bias+01_BK-1105_045fil_TI_005.dat  AS-2-2-bias+01_BK-1105_045fil_TI_006.dat | PCR positive saliva for test | Figs. 3g, 3j, 3k S15, Table S6, Data S2 |
|  | test/Positive/AS-2-2-bias+01_BK-1149_045fil_TI | AS-2-2-bias+01_BK-1149_045fil_TI_009.dat | PCR positive saliva for test | Figs. 3g, 3j, 3k S15, Table S6, Data S2 |
|  | test/Positive/AS-2-2-bias+01_BK-950_045fil_TI_1st | AS-2-2-bias+01_BK-950_045fil_TI_1st_007.dat  AS-2-2-bias+01_BK-950_045fil_TI_1st_008.dat  AS-2-2-bias+01_BK-950_045fil_TI_1st_009.dat | PCR positive saliva for test | Figs. 3g, 3j, 3k S15, Table S6, Data S2 |
|  | test/Positive/AS-2-2-bias+01_BK-967_045fil_TI_1st | AS-2-2-bias+01_BK-967_045fil_TI_1st_001.dat  AS-2-2-bias+01_BK-967_045fil_TI_1st_002.dat  AS-2-2-bias+01_BK-967_045fil_TI_1st_003.dat | PCR positive saliva for test | Figs. 3g, 3j, 3k S15, Table S6, Data S2 |
|  | test/Positive/AS-2-2-bias+01_BK-986_045fil_TI_1st | AS-2-2-bias+01_BK-986_045fil_TI_1st_003.dat  AS-2-2-bias+01_BK-986_045fil_TI_1st_005.dat  AS-2-2-bias+01_BK-986_045fil_TI_1st_006.dat | PCR positive saliva for test | Figs. 3g, 3j, 3k S15, Table S6, Data S2 |
|  | test/Positive/F7 day5 | F7 day5_001.dat | PCR positive saliva for test | Figs. 3g, 3j, 3k S15, Table S6, Data S2 |
|  | training/Positive/AS-2-2-bias+01_BK-985_045fil_TI_1s | AS-2-2-bias+01_BK-985_045fil_TI_1st_009.dat | PCR positive saliva for training | Figs. 3a-3c, 3f, 3h, 3i, S13, S14, Table S5, Data S1 |
|  | training/Positive/F8 day3 | F8 day3_001.dat | PCR positive saliva for training | Figs. 3a-3c, 3f, 3h, 3i, S13, S14, Table S5, Data S1 |
| Saliva(n=100 HO)_I-t data-20210209T093219Z-043.zip | test/Negative/HD-112720-12 | HD-112720-12_003.dat  HD-112720-12_004.dat | PCR negative saliva for test | Figs. 3g, 3j, 3k S15, Table S6, Data S2 |
|  | test/Positive/AS-2-2-bias+01_BK-1103_045fil_TI | AS-2-2-bias+01_BK-1103_045fil_TI_004.dat  AS-2-2-bias+01_BK-1103_045fil_TI_005.dat  AS-2-2-bias+01_BK-1103_045fil_TI_006.dat | PCR positive saliva for test | Figs. 3g, 3j, 3k S15, Table S6, Data S2 |
|  | test/Positive/AS-2-2-bias+01_BK-1105_045fil_TI | AS-2-2-bias+01_BK-1105_045fil_TI_003.dat  AS-2-2-bias+01_BK-1105_045fil_TI_004.dat | PCR positive saliva for test | Figs. 3g, 3j, 3k S15, Table S6, Data S2 |
|  | test/Positive/AS-2-2-bias+01_BK-950_045fil_TI_1st | AS-2-2-bias+01_BK-950_045fil_TI_1st_005.dat  AS-2-2-bias+01_BK-950_045fil_TI_1st_006.dat | PCR positive saliva for test | Figs. 3g, 3j, 3k S15, Table S6, Data S2 |
|  | test/Positive/ AS-2-2-bias+01_BK-986_045fil_TI_1st | AS-2-2-bias+01_BK-986_045fil_TI_1st_001.dat  AS-2-2-bias+01_BK-986_045fil_TI_1st_002.dat  AS-2-2-bias+01_BK-986_045fil_TI_1st_004.dat | PCR positive saliva for test | Figs. 3g, 3j, 3k S15, Table S6, Data S2 |
|  | test/Positive/F5 day14 | F5 day14_008.dat | PCR positive saliva for test | Figs. 3g, 3j, 3k S15, Table S6, Data S2 |
|  | training/Positive/AS-2-2-bias+01_BK-985_045fil_TI_1st | AS-2-2-bias+01_BK-985_045fil_TI_1st_008.dat | PCR positive saliva for training | Figs. 3a-3c, 3f, 3h, 3i, S13, S14, Table S5, Data S1 |
|  | training/Positive/F1 day3 | F1 day3_009.dat | PCR positive saliva for training | Figs. 3a-3c, 3f, 3h, 3i, S13, S14, Table S5, Data S1 |
| Saliva(n=100 HO)_I-t data-20210209T093219Z-044.zip | test/Negative/HD-112720-12 | HD-112720-12_001.dat  HD-112720-12_002.dat | PCR negative saliva for test | Figs. 3g, 3j, 3k S15, Table S6, Data S2 |
|  | test/Positive/AS-2-2-bias+01_BK-1103_045fil_TI | AS-2-2-bias+01_BK-1103_045fil_TI_001.dat  AS-2-2-bias+01_BK-1103_045fil_TI_002.dat  AS-2-2-bias+01_BK-1103_045fil_TI_003.dat | PCR positive saliva for test | Figs. 3g, 3j, 3k S15, Table S6, Data S2 |
|  | test/Positive/AS-2-2-bias+01_BK-1105_045fil_TI | AS-2-2-bias+01_BK-1105_045fil_TI_001.dat  AS-2-2-bias+01_BK-1105_045fil_TI_002.dat | PCR positive saliva for test | Figs. 3g, 3j, 3k S15, Table S6, Data S2 |
|  | test/Positive/AS-2-2-bias+01_BK-1149_045fil_TI | AS-2-2-bias+01_BK-1149_045fil_TI_007.dat | PCR positive saliva for test | Figs. 3g, 3j, 3k S15, Table S6, Data S2 |
|  | test/Positive/AS-2-2-bias+01_BK-950_045fil_TI_1st | AS-2-2-bias+01_BK-950_045fil_TI_1st_002.dat  AS-2-2-bias+01_BK-950_045fil_TI_1st_004.dat | PCR positive saliva for test | Figs. 3g, 3j, 3k S15, Table S6, Data S2 |
|  | test/Positive/F5 day14 | F5 day14_007.dat | PCR positive saliva for test | Figs. 3g, 3j, 3k S15, Table S6, Data S2 |
|  | training/Positive/AS-2-2-bias+01_BK-960_045fil_TI_1st | AS-2-2-bias+01_BK-960_045fil_TI_1st_009.dat | PCR positive saliva for training | Figs. 3a-3c, 3f, 3h, 3i, S13, S14, Table S5, Data S1 |
|  | training/Positive/AS-2-2-bias+01_BK-985_045fil_TI_1st | AS-2-2-bias+01_BK-985_045fil_TI_1st_003.dat  AS-2-2-bias+01_BK-985_045fil_TI_1st_004.dat  AS-2-2-bias+01_BK-985_045fil_TI_1st_007.dat | PCR positive saliva for training | Figs. 3a-3c, 3f, 3h, 3i, S13, S14, Table S5, Data S1 |
| Saliva(n=100 HO)_I-t data-20210209T093219Z-045.zip | test/Positive/AS-2-2-bias+01_BK-1149_045fil_TI | AS-2-2-bias+01_BK-1149_045fil_TI_004.dat  AS-2-2-bias+01_BK-1149_045fil_TI_005.dat  AS-2-2-bias+01_BK-1149_045fil_TI_006.dat  AS-2-2-bias+01_BK-1149_045fil_TI_008.dat | PCR positive saliva for test | Figs. 3g, 3j, 3k S15, Table S6, Data S2 |
|  | test/Positive/AS-2-2-bias+01_BK-950_045fil_TI_1st | AS-2-2-bias+01_BK-950_045fil_TI_1st_001.dat | PCR positive saliva for test | Figs. 3g, 3j, 3k S15, Table S6, Data S2 |
|  | test/Positive/F5 day14 | F5 day14_001.dat  F5 day14_002.dat  F5 day14_004.dat  F5 day14_005.dat  F5 day14_006.dat | PCR positive saliva for test | Figs. 3g, 3j, 3k S15, Table S6, Data S2 |
|  | training/Positive/AS-2-2-bias+01_BK-960_045fil_TI_1st | AS-2-2-bias+01_BK-960_045fil_TI_1st_006.dat | PCR positive saliva for training | Figs. 3a-3c, 3f, 3h, 3i, S13, S14, Table S5, Data S1 |
|  | training/Positive/AS-2-2-bias+01_BK-985_045fil_TI_1st | AS-2-2-bias+01_BK-985_045fil_TI_1st_005.dat  AS-2-2-bias+01_BK-985_045fil_TI_1st_006.dat | PCR positive saliva for training | Figs. 3a-3c, 3f, 3h, 3i, S13, S14, Table S5, Data S1 |
|  | training/Positive/F1 day3 | F1 day3_006.dat  F1 day3_008.dat | PCR positive saliva for training | Figs. 3a-3c, 3f, 3h, 3i, S13, S14, Table S5, Data S1 |
| Saliva(n=100 HO)_I-t data-20210209T093219Z-046.zip | test/Positive/AS-2-2-bias+01_BK-1149_045fil_TI | AS-2-2-bias+01_BK-1149_045fil_TI_002.dat  AS-2-2-bias+01_BK-1149_045fil_TI_003.dat | PCR positive saliva for test | Figs. 3g, 3j, 3k S15, Table S6, Data S2 |
|  | test/Positive/AS-2-2-bias+01_BK-964_045fil_TI_3rd | AS-2-2-bias+01_BK-964_045fil_TI_3rd_004.dat  AS-2-2-bias+01_BK-964_045fil_TI_3rd_006.dat  AS-2-2-bias+01_BK-964_045fil_TI_3rd_007.dat  AS-2-2-bias+01_BK-964_045fil_TI_3rd_008.dat | PCR positive saliva for test | Figs. 3g, 3j, 3k S15, Table S6, Data S2 |
|  | test/Positive/AS-2-2-bias+01_BK-972_045fil_TI_1st | AS-2-2-bias+01_BK-972_045fil_TI_1st_008.dat | PCR positive saliva for test | Figs. 3g, 3j, 3k S15, Table S6, Data S2 |
|  | training/Positive/AS-2-2-bias+01_BK-960_045fil_TI_1st | AS-2-2-bias+01_BK-960_045fil_TI_1st_001.dat  AS-2-2-bias+01_BK-960_045fil_TI_1st_003.dat  AS-2-2-bias+01_BK-960_045fil_TI_1st_004.dat  AS-2-2-bias+01_BK-960_045fil_TI_1st_005.dat  AS-2-2-bias+01_BK-960_045fil_TI_1st_008.dat | PCR positive saliva for training | Figs. 3a-3c, 3f, 3h, 3i, S13, S14, Table S5, Data S1 |
|  | training/Positive/F1 day3 | F1 day3_005.dat  F1 day3_007.dat | PCR positive saliva for training | Figs. 3a-3c, 3f, 3h, 3i, S13, S14, Table S5, Data S1 |
|  | training/Positive/F4 | F4_009.dat | PCR positive saliva for training | Figs. 3a-3c, 3f, 3h, 3i, S13, S14, Table S5, Data S1 |
| Saliva(n=100 HO)_I-t data-20210209T093219Z-047.zip | test/Negative/HD-112720-20 | HD-112720-20_003.dat | PCR negative saliva for test | Figs. 3g, 3j, 3k S15, Table S6, Data S2 |
|  | test/Positive/AS-2-2-bias+01_BK-964_045fil_TI_3rd | AS-2-2-bias+01_BK-964_045fil_TI_3rd_002.dat  AS-2-2-bias+01_BK-964_045fil_TI_3rd_003.dat  AS-2-2-bias+01_BK-964_045fil_TI_3rd_005.dat | PCR positive saliva for test | Figs. 3g, 3j, 3k S15, Table S6, Data S2 |
|  | test/Positive/AS-2-2-bias+01_BK-972_045fil_TI_1st | AS-2-2-bias+01_BK-972_045fil_TI_1st_003.dat  AS-2-2-bias+01_BK-972_045fil_TI_1st_004.dat  AS-2-2-bias+01_BK-972_045fil_TI_1st_005.dat  AS-2-2-bias+01_BK-972_045fil_TI_1st_006.dat  AS-2-2-bias+01_BK-972_045fil_TI_1st_007.dat | PCR positive saliva for test | Figs. 3g, 3j, 3k S15, Table S6, Data S2 |
|  | test/Positive/AS-2-2-bias+01_BK-983_045fil_TI_1st | AS-2-2-bias+01_BK-983_045fil_TI_1st_005.dat  AS-2-2-bias+01_BK-983_045fil_TI_1st_006.dat  AS-2-2-bias+01_BK-983_045fil_TI_1st_007.dat  AS-2-2-bias+01_BK-983_045fil_TI_1st_008.dat | PCR positive saliva for test | Figs. 3g, 3j, 3k S15, Table S6, Data S2 |
|  | training/Positive/F1 day3 | F1 day3_002.dat  F1 day3_004.dat | PCR positive saliva for training | Figs. 3a-3c, 3f, 3h, 3i, S13, S14, Table S5, Data S1 |
| Saliva(n=100 HO)_I-t data-20210209T093219Z-048.zip | test/Negative/HD-112720-20 | HD-112720-20_001.dat  HD-112720-20_002.dat  HD-112720-20_004.dat | PCR negative saliva for test | Figs. 3g, 3j, 3k S15, Table S6, Data S2 |
|  | test/Positive/AS-2-2-bias+01_BK-964_045fil_TI_3rd | AS-2-2-bias+01_BK-964_045fil_TI_3rd_001.dat | PCR positive saliva for test | Figs. 3g, 3j, 3k S15, Table S6, Data S2 |
|  | test/Positive/AS-2-2-bias+01_BK-972_045fil_TI_1st | AS-2-2-bias+01_BK-972_045fil_TI_1st_001.dat  AS-2-2-bias+01_BK-972_045fil_TI_1st_002.dat | PCR positive saliva for test | Figs. 3g, 3j, 3k S15, Table S6, Data S2 |
|  | test/Positive/AS-2-2-bias+01_BK-974_045fil_TI_1st | AS-2-2-bias+01_BK-974_045fil_TI_1st_009.dat | PCR positive saliva for test | Figs. 3g, 3j, 3k S15, Table S6, Data S2 |
|  | test/Positive/AS-2-2-bias+01_BK-983_045fil_TI_1st | AS-2-2-bias+01_BK-983_045fil_TI_1st_003.dat  AS-2-2-bias+01_BK-983_045fil_TI_1st_004.dat | PCR positive saliva for test | Figs. 3g, 3j, 3k S15, Table S6, Data S2 |
|  | test/Positive/F3 | F3_009.dat | PCR positive saliva for test | Figs. 3g, 3j, 3k S15, Table S6, Data S2 |
|  | training/Positive/AS-2-2-bias+01_BK-1123_045fil_TI | AS-2-2-bias+01_BK-1123_045fil_TI_009.dat | PCR positive saliva for training | Figs. 3a-3c, 3f, 3h, 3i, S13, S14, Table S5, Data S1 |
|  | training/Positive/AS-2-2-bias+01_BK-1125_045fil_TI | AS-2-2-bias+01_BK-1125_045fil_TI_002.dat | PCR positive saliva for training | Figs. 3a-3c, 3f, 3h, 3i, S13, S14, Table S5, Data S1 |
|  | training/Positive/F1 day3 | F1 day3_001.dat | PCR positive saliva for training | Figs. 3a-3c, 3f, 3h, 3i, S13, S14, Table S5, Data S1 |
|  | training/Positive/F4 | F4_005.dat  F4_005.dat  F4_005.dat | PCR positive saliva for training | Figs. 3a-3c, 3f, 3h, 3i, S13, S14, Table S5, Data S1 |
| Saliva(n=100 HO)_I-t data-20210209T093219Z-049.zip | test/Negative/HD-120420-36 | HD-120420-36_001.dat  HD-120420-36_002.dat  HD-120420-36_003.dat  HD-120420-36_004.dat | PCR negative saliva for test | Figs. 3g, 3j, 3k S15, Table S6, Data S2 |
|  | test/Positive/AS-2-2-bias+01_BK-974_045fil_TI_1st | AS-2-2-bias+01_BK-974_045fil_TI_1st_008.dat | PCR positive saliva for test | Figs. 3g, 3j, 3k S15, Table S6, Data S2 |
|  | test/Positive/AS-2-2-bias+01_BK-983_045fil_TI_1st | AS-2-2-bias+01_BK-983_045fil_TI_1st_001.dat  AS-2-2-bias+01_BK-983_045fil_TI_1st_002.dat | PCR positive saliva for test | Figs. 3g, 3j, 3k S15, Table S6, Data S2 |
|  | test/Positive/F3 | F3_006.dat  F3_007.dat  F3_008.dat | PCR positive saliva for test | Figs. 3g, 3j, 3k S15, Table S6, Data S2 |
|  | training/Negative/HD-112720-36 | HD-112720-36_003.dat  HD-112720-36_004.dat | PCR negative saliva for training | Figs. 3a-3c, 3f, 3h, 3i, S13, S14, Table S5, Data S1 |
|  | training/Positive/AS-2-2-bias+01_BK-1125_045fil_TI | AS-2-2-bias+01_BK-1125_045fil_TI_003.dat | PCR positive saliva for training | Figs. 3a-3c, 3f, 3h, 3i, S13, S14, Table S5, Data S1 |
|  | training/Positive/F4 | F4_003.dat  F4_007.dat | PCR positive saliva for training | Figs. 3a-3c, 3f, 3h, 3i, S13, S14, Table S5, Data S1 |
| Saliva(n=100 HO)_I-t data-20210209T093219Z-050.zip | test/Positive/AS-2-2-bias+01_BK-968_045fil_TI_1st | AS-2-2-bias+01_BK-968_045fil_TI_1st_009.dat | PCR positive saliva for test | Figs. 3g, 3j, 3k S15, Table S6, Data S2 |
|  | test/Positive/AS-2-2-bias+01_BK-974_045fil_TI_1st | AS-2-2-bias+01_BK-974_045fil_TI_1st_006.dat  AS-2-2-bias+01_BK-974_045fil_TI_1st_007.dat | PCR positive saliva for test | Figs. 3g, 3j, 3k S15, Table S6, Data S2 |
|  | test/Positive/F3 | F3_001.dat  F3_003.dat  F3_004.dat  F3_005.dat | PCR positive saliva for test | Figs. 3g, 3j, 3k S15, Table S6, Data S2 |
|  | training/Negative/HD-112720-36 | HD-112720-36_001.dat | PCR negative saliva for training | Figs. 3a-3c, 3f, 3h, 3i, S13, S14, Table S5, Data S1 |
|  | training/Positive/ AS-2-2-bias+01_BK-1123_045fil_TI | AS-2-2-bias+01_BK-1123_045fil_TI_004.dat  AS-2-2-bias+01_BK-1123_045fil_TI_005.dat  AS-2-2-bias+01_BK-1123_045fil_TI_006.dat  AS-2-2-bias+01_BK-1123_045fil_TI_007.dat | PCR positive saliva for training | Figs. 3a-3c, 3f, 3h, 3i, S13, S14, Table S5, Data S1 |
|  | training/Positive/AS-2-2-bias+01_BK-1125_045fil_TI | AS-2-2-bias+01_BK-1125_045fil_TI_001.dat | PCR positive saliva for training | Figs. 3a-3c, 3f, 3h, 3i, S13, S14, Table S5, Data S1 |
|  | training/Positive/AS-2-2-bias+01_BK-960_045fil_TI_1st | AS-2-2-bias+01_BK-960_045fil_TI_1st_007.dat | PCR positive saliva for training | Figs. 3a-3c, 3f, 3h, 3i, S13, S14, Table S5, Data S1 |
|  | training/Positive/F4 | F4_001.dat | PCR positive saliva for training | Figs. 3a-3c, 3f, 3h, 3i, S13, S14, Table S5, Data S1 |
| Saliva(n=100 HO)_I-t data-20210209T093219Z-051.zip | test/Positive/AS-2-2-bias+01_BK-958_045fil_TI_1st | AS-2-2-bias+01_BK-958_045fil_TI_1st_006.dat  AS-2-2-bias+01_BK-958_045fil_TI_1st_007.dat  AS-2-2-bias+01_BK-958_045fil_TI_1st_008.dat | PCR positive saliva for test | Figs. 3g, 3j, 3k S15, Table S6, Data S2 |
|  | test/Positive/AS-2-2-bias+01_BK-968_045fil_TI_1st | AS-2-2-bias+01_BK-968_045fil_TI_1st_005.dat  AS-2-2-bias+01_BK-968_045fil_TI_1st_006.dat  AS-2-2-bias+01_BK-968_045fil_TI_1st_007.dat  AS-2-2-bias+01_BK-968_045fil_TI_1st_008.dat | PCR positive saliva for test | Figs. 3g, 3j, 3k S15, Table S6, Data S2 |
|  | test/Positive/AS-2-2-bias+01_BK-974_045fil_TI_1st | AS-2-2-bias+01_BK-974_045fil_TI_1st_001.dat  AS-2-2-bias+01_BK-974_045fil_TI_1st_002.dat  AS-2-2-bias+01_BK-974_045fil_TI_1st_003.dat  AS-2-2-bias+01_BK-974_045fil_TI_1st_004.dat  AS-2-2-bias+01_BK-974_045fil_TI_1st_005.dat | PCR positive saliva for test | Figs. 3g, 3j, 3k S15, Table S6, Data S2 |
|  | test/Positive/F3 | F3_002.dat | PCR positive saliva for test | Figs. 3g, 3j, 3k S15, Table S6, Data S2 |
|  | training/Positive/AS-2-2-bias+01_BK-1123_045fil_TI | AS-2-2-bias+01_BK-1123_045fil_TI_002.dat  AS-2-2-bias+01_BK-1123_045fil_TI_003.dat | PCR positive saliva for training | Figs. 3a-3c, 3f, 3h, 3i, S13, S14, Table S5, Data S1 |
| Saliva(n=100 HO)_I-t data-20210209T093219Z-052.zip | test/Negative/HD-120220-11 | HD-120220-11_003.dat  HD-120220-11_004.dat | PCR negative saliva for test | Figs. 3g, 3j, 3k S15, Table S6, Data S2 |
|  | test/Positive/AS-2-2-bias+01_BK-1096_045fil_TI | AS-2-2-bias+01_BK-1096_045fil_TI_005.dat  AS-2-2-bias+01_BK-1096_045fil_TI_007.dat  AS-2-2-bias+01_BK-1096_045fil_TI_008.dat  AS-2-2-bias+01_BK-1096_045fil_TI_009.dat | PCR positive saliva for test | Figs. 3g, 3j, 3k S15, Table S6, Data S2 |
|  | test/Positive/AS-2-2-bias+01_BK-958_045fil_TI_1st | AS-2-2-bias+01_BK-958_045fil_TI_1st_003.dat  AS-2-2-bias+01_BK-958_045fil_TI_1st_004.dat  AS-2-2-bias+01_BK-958_045fil_TI_1st_005.dat | PCR positive saliva for test | Figs. 3g, 3j, 3k S15, Table S6, Data S2 |
|  | test/Positive/AS-2-2-bias+01_BK-968_045fil_TI_1st | AS-2-2-bias+01_BK-968_045fil_TI_1st_001.dat  AS-2-2-bias+01_BK-968_045fil_TI_1st_002.dat  AS-2-2-bias+01_BK-968_045fil_TI_1st_003.dat  AS-2-2-bias+01_BK-968_045fil_TI_1st_004.dat | PCR positive saliva for test | Figs. 3g, 3j, 3k S15, Table S6, Data S2 |
|  | test/Positive/AS-2-2-bias+01_BK-971_045fil_TI_1st | AS-2-2-bias+01_BK-971_045fil_TI_1st_009.dat | PCR positive saliva for test | Figs. 3g, 3j, 3k S15, Table S6, Data S2 |
|  | test/Positive/F2 day14 | F2 day14_009.dat126.6 MB | PCR positive saliva for test | Figs. 3g, 3j, 3k S15, Table S6, Data S2 |
|  | training/Positive/AS-2-2-bias+01_BK-1123_045fil_TI | AS-2-2-bias+01_BK-1123_045fil_TI_001.dat | PCR positive saliva for training | Figs. 3a-3c, 3f, 3h, 3i, S13, S14, Table S5, Data S1 |
| Saliva(n=100 HO)_I-t data-20210209T093219Z-053.zip | test/Negative/HD-120220-11 | HD-120220-11_002.dat | PCR negative saliva for test | Figs. 3g, 3j, 3k S15, Table S6, Data S2 |
|  | test/Positive/AS-2-2-bias+01_BK-1096_045fil_TI | AS-2-2-bias+01_BK-1096_045fil_TI_001.dat  AS-2-2-bias+01_BK-1096_045fil_TI_004.dat  AS-2-2-bias+01_BK-1096_045fil_TI_006.dat | PCR positive saliva for test | Figs. 3g, 3j, 3k S15, Table S6, Data S2 |
|  | test/Positive/AS-2-2-bias+01_BK-1152_045fil_TI | AS-2-2-bias+01_BK-1152_045fil_TI_009.dat | PCR positive saliva for test | Figs. 3g, 3j, 3k S15, Table S6, Data S2 |
|  | test/Positive/AS-2-2-bias+01_BK-958_045fil_TI_1st | AS-2-2-bias+01_BK-958_045fil_TI_1st_001.dat | PCR positive saliva for test | Figs. 3g, 3j, 3k S15, Table S6, Data S2 |
|  | test/Positive/AS-2-2-bias+01_BK-971_045fil_TI_1st | AS-2-2-bias+01_BK-971_045fil_TI_1st_004.dat  AS-2-2-bias+01_BK-971_045fil_TI_1st_005.dat  AS-2-2-bias+01_BK-971_045fil_TI_1st_008.dat | PCR positive saliva for test | Figs. 3g, 3j, 3k S15, Table S6, Data S2 |
|  | test/Positive/F2 day14 | F2 day14_003.dat  F2 day14_004.dat | PCR positive saliva for test | Figs. 3g, 3j, 3k S15, Table S6, Data S2 |
|  | test/Positive/F9 day3 | F9 day3_009.dat | PCR positive saliva for test | Figs. 3g, 3j, 3k S15, Table S6, Data S2 |
|  | training/Positive/AS-2-2-bias+01_BK-1153_045fil_TI | AS-2-2-bias+01_BK-1153_045fil_TI_009.dat | PCR positive saliva for training | Figs. 3a-3c, 3f, 3h, 3i, S13, S14, Table S5, Data S1 |
|  | training/Positive/F6 day1 | F6 day1_001.dat  F6 day1_006.dat  F6 day1_007.dat | PCR positive saliva for training | Figs. 3a-3c, 3f, 3h, 3i, S13, S14, Table S5, Data S1 |
| Saliva(n=100 HO)_I-t data-20210209T093219Z-054.zip | test/Negative/HD-120220-11 | HD-120220-11_001.dat | PCR negative saliva for test | Figs. 3g, 3j, 3k S15, Table S6, Data S2 |
|  | test/Positive/AS-2-2-bias+01_BK-1096_045fil_TI | AS-2-2-bias+01_BK-1096_045fil_TI_003.dat | PCR positive saliva for test | Figs. 3g, 3j, 3k S15, Table S6, Data S2 |
|  | test/Positive/AS-2-2-bias+01_BK-971_045fil_TI_1st | AS-2-2-bias+01_BK-971_045fil_TI_1st_002.dat  AS-2-2-bias+01_BK-971_045fil_TI_1st_003.dat  AS-2-2-bias+01_BK-971_045fil_TI_1st_006.dat  AS-2-2-bias+01_BK-971_045fil_TI_1st_007.dat | PCR positive saliva for test | Figs. 3g, 3j, 3k S15, Table S6, Data S2 |
|  | test/Positive/F2 day14 | F2 day14_001.dat  F2 day14_002.dat  F2 day14_006.dat  F2 day14_007.dat | PCR positive saliva for test | Figs. 3g, 3j, 3k S15, Table S6, Data S2 |
|  | test/Positive/F9 day3 | F9 day3_007.dat  F9 day3_008.dat | PCR positive saliva for test | Figs. 3g, 3j, 3k S15, Table S6, Data S2 |
|  | training/Positive/F6 day1 | F6 day1_002.dat  F6 day1_003.dat  F6 day1_004.dat | PCR positive saliva for training | Figs. 3a-3c, 3f, 3h, 3i, S13, S14, Table S5, Data S1 |
| Saliva(n=100 HO)_I-t data-20210209T093219Z-055.zip | test/Positive/AS-2-2-bias+01_BK-1152_045fil_TI | AS-2-2-bias+01_BK-1152_045fil_TI_004.dat  AS-2-2-bias+01_BK-1152_045fil_TI_006.dat  AS-2-2-bias+01_BK-1152_045fil_TI_007.dat | PCR positive saliva for test | Figs. 3g, 3j, 3k S15, Table S6, Data S2 |
|  | test/Positive/AS-2-2-bias+01_BK-971_045fil_TI_1st | AS-2-2-bias+01_BK-971_045fil_TI_1st_001.dat | PCR positive saliva for test | Figs. 3g, 3j, 3k S15, Table S6, Data S2 |
|  | test/Positive/F9 day3 | F9 day3_001.dat  F9 day3_005.dat  F9 day3_006.dat | PCR positive saliva for test | Figs. 3g, 3j, 3k S15, Table S6, Data S2 |
|  | training/Positive/AS-2-2-bias+01_BK-1146_045fil_TI | AS-2-2-bias+01_BK-1146_045fil_TI_005.dat  AS-2-2-bias+01_BK-1146_045fil_TI_006.dat  AS-2-2-bias+01_BK-1146_045fil_TI_009.dat | PCR positive saliva for training | Figs. 3a-3c, 3f, 3h, 3i, S13, S14, Table S5, Data S1 |
|  | training/Positive/AS-2-2-bias+01_BK-1153_045fil_TI | AS-2-2-bias+01_BK-1153_045fil_TI_003.dat  AS-2-2-bias+01_BK-1153_045fil_TI_007.dat  AS-2-2-bias+01_BK-1153_045fil_TI_008.dat | PCR positive saliva for training | Figs. 3a-3c, 3f, 3h, 3i, S13, S14, Table S5, Data S1 |
|  | training/Positive/AS-2-2-bias+01_BK-992_045fil_TI_1st | AS-2-2-bias+01_BK-992_045fil_TI_1st_003.dat  AS-2-2-bias+01_BK-992_045fil_TI_1st_006.dat | PCR positive saliva for training | Figs. 3a-3c, 3f, 3h, 3i, S13, S14, Table S5, Data S1 |
| Saliva(n=100 HO)_I-t data-20210209T093219Z-056.zip | test/Positive/AS-2-2-bias+01_BK-1152_045fil_TI | AS-2-2-bias+01_BK-1152_045fil_TI_001.dat  AS-2-2-bias+01_BK-1152_045fil_TI_002.dat  AS-2-2-bias+01_BK-1152_045fil_TI_003.dat  AS-2-2-bias+01_BK-1152_045fil_TI_005.dat | PCR positive saliva for test | Figs. 3g, 3j, 3k S15, Table S6, Data S2 |
|  | test/Positive/F9 day3 | F9 day3_003.dat | PCR positive saliva for test | Figs. 3g, 3j, 3k S15, Table S6, Data S2 |
|  | training/Positive/AS-2-2-bias+01_BK-1146_045fil_TI | AS-2-2-bias+01_BK-1146_045fil_TI_002.dat  AS-2-2-bias+01_BK-1146_045fil_TI_003.dat  AS-2-2-bias+01_BK-1146_045fil_TI_004.dat  AS-2-2-bias+01_BK-1146_045fil_TI_008.dat | PCR positive saliva for training | Figs. 3a-3c, 3f, 3h, 3i, S13, S14, Table S5, Data S1 |
|  | training/Positive/AS-2-2-bias+01_BK-1153_045fil_TI | AS-2-2-bias+01_BK-1153_045fil_TI_001.dat  AS-2-2-bias+01_BK-1153_045fil_TI_004.dat  AS-2-2-bias+01_BK-1153_045fil_TI_005.dat  AS-2-2-bias+01_BK-1153_045fil_TI_006.dat | PCR positive saliva for training | Figs. 3a-3c, 3f, 3h, 3i, S13, S14, Table S5, Data S1 |
|  | training/Positive/AS-2-2-bias+01_BK-992_045fil_TI_1st | AS-2-2-bias+01_BK-992_045fil_TI_1st_008.dat  AS-2-2-bias+01_BK-992_045fil_TI_1st_009.dat | PCR positive saliva for training | Figs. 3a-3c, 3f, 3h, 3i, S13, S14, Table S5, Data S1 |
| Saliva(n=100 HO)_I-t data-20210209T093219Z-057.zip | test/Negative/HD-120420-6 | HD-120420-6_001.dat  HD-120420-6_002.dat  HD-120420-6_003.dat  HD-120420-6_004.dat | PCR negative saliva for test | Figs. 3g, 3j, 3k S15, Table S6, Data S2 |
|  | test/Positive/F9 day3 | F9 day3_002.dat | PCR positive saliva for test | Figs. 3g, 3j, 3k S15, Table S6, Data S2 |
|  | training/Positive/AS-2-2-bias+01_BK-1124_045fil_TI | AS-2-2-bias+01_BK-1124_045fil_TI_001.dat  AS-2-2-bias+01_BK-1124_045fil_TI_003.dat  AS-2-2-bias+01_BK-1124_045fil_TI_006.dat  AS-2-2-bias+01_BK-1124_045fil_TI_007.dat  AS-2-2-bias+01_BK-1124_045fil_TI_008.dat | PCR positive saliva for training | Figs. 3a-3c, 3f, 3h, 3i, S13, S14, Table S5, Data S1 |
|  | training/Positive/AS-2-2-bias+01_BK-1146_045fil_TI | AS-2-2-bias+01_BK-1146_045fil_TI_007.dat | PCR positive saliva for training | Figs. 3a-3c, 3f, 3h, 3i, S13, S14, Table S5, Data S1 |
|  | training/Positive/AS-2-2-bias+01_BK-991_045fil_TI_1st | AS-2-2-bias+01_BK-991_045fil_TI_1st_007.dat  AS-2-2-bias+01_BK-991_045fil_TI_1st_009.dat | PCR positive saliva for training | Figs. 3a-3c, 3f, 3h, 3i, S13, S14, Table S5, Data S1 |
|  | training/Positive/AS-2-2-bias+01_BK-992_045fil_TI_1st | AS-2-2-bias+01_BK-992_045fil_TI_1st_001.dat  AS-2-2-bias+01_BK-992_045fil_TI_1st_007.dat | PCR positive saliva for training | Figs. 3a-3c, 3f, 3h, 3i, S13, S14, Table S5, Data S1 |
| Saliva(n=100 HO)_I-t data-20210209T093219Z-058.zip | test/Negative/HD-112720-1 | HD-112720-1_001.dat  HD-112720-1_002.dat  HD-112720-1_003.dat  HD-112720-1_004.dat | PCR negative saliva for test | Figs. 3g, 3j, 3k S15, Table S6, Data S2 |
|  | training/Negative/HD-113020-24 | HD-113020-24_001.dat  HD-113020-24_002.dat  HD-113020-24_003.dat  HD-113020-24_004.dat | PCR negative saliva for training | Figs. 3a-3c, 3f, 3h, 3i, S13, S14, Table S5, Data S1 |
|  | training/Positive/AS-2-2-bias+01_BK-1124_045fil_TI | AS-2-2-bias+01_BK-1124_045fil_TI_009.dat | PCR positive saliva for training | Figs. 3a-3c, 3f, 3h, 3i, S13, S14, Table S5, Data S1 |
|  | training/Positive/AS-2-2-bias+01_BK-991_045fil_TI_1st | AS-2-2-bias+01_BK-991_045fil_TI_1st_001.dat  AS-2-2-bias+01_BK-991_045fil_TI_1st_002.dat  AS-2-2-bias+01_BK-991_045fil_TI_1st_006.dat | PCR positive saliva for training | Figs. 3a-3c, 3f, 3h, 3i, S13, S14, Table S5, Data S1 |
|  | training/Positive/F2 day5 | F2 day5_002.dat  F2 day5_005.dat  F2 day5_007.dat | PCR positive saliva for training | Figs. 3a-3c, 3f, 3h, 3i, S13, S14, Table S5, Data S1 |
| Saliva(n=100 HO)_I-t data-20210209T093219Z-059.zip | test/Negative/HD-120220-29 | HD-120220-29_001.dat  HD-120220-29_002.dat  HD-120220-29_003.dat  HD-120220-29_004.dat | PCR negative saliva for test | Figs. 3g, 3j, 3k S15, Table S6, Data S2 |
|  | test/Negative/HD-120420-8 | HD-120420-8_001.dat  HD-120420-8_004.dat | PCR negative saliva for test | Figs. 3g, 3j, 3k S15, Table S6, Data S2 |
|  | test/Positive/F14 day1 | F14 day1_009.dat | PCR positive saliva for training | Figs. 3a-3c, 3f, 3h, 3i, S13, S14, Table S5, Data S1 |
|  | training/Positive/AS-2-2-bias+01_BK-1124_045fil_TI | AS-2-2-bias+01_BK-1124_045fil_TI_005.dat | PCR positive saliva for training | Figs. 3a-3c, 3f, 3h, 3i, S13, S14, Table S5, Data S1 |
|  | training/Positive/AS-2-2-bias+01_BK-991_045fil_TI_1st | AS-2-2-bias+01_BK-991_045fil_TI_1st_003.dat  AS-2-2-bias+01_BK-991_045fil_TI_1st_004.dat  AS-2-2-bias+01_BK-991_045fil_TI_1st_005.dat | PCR positive saliva for training | Figs. 3a-3c, 3f, 3h, 3i, S13, S14, Table S5, Data S1 |
|  | training/Positive/F2 day5 | F2 day5_001.dat  F2 day5_003.dat  F2 day5_004.dat  F2 day5_008.dat | PCR positive saliva for training | Figs. 3a-3c, 3f, 3h, 3i, S13, S14, Table S5, Data S1 |
| Saliva(n=100 HO)_I-t data-20210209T093219Z-060.zip | test/Negative/HD-120420-21 | HD-120420-21_002.dat | PCR negative saliva for test | Figs. 3g, 3j, 3k S15, Table S6, Data S2 |
|  | test/Negative/HD-120420-8 | HD-120420-8_002.dat  HD-120420-8_003.dat | PCR negative saliva for test | Figs. 3g, 3j, 3k S15, Table S6, Data S2 |
|  | test/Positive/F14 day1 | F14 day1_001.dat  F14 day1_002.dat  F14 day1_003.dat  F14 day1_005.dat  F14 day1_006.dat | PCR positive saliva for test | Figs. 3g, 3j, 3k S15, Table S6, Data S2 |
|  | training/Negative/HD-112720-10 | HD-112720-10_001.dat  HD-112720-10_002.dat | PCR negative saliva for training | Figs. 3a-3c, 3f, 3h, 3i, S13, S14, Table S5, Data S1 |
|  | training/Positive/AS-2-2-bias+01_BK-1100_045fil_TI | AS-2-2-bias+01_BK-1100_045fil_TI_001.dat  AS-2-2-bias+01_BK-1100_045fil_TI_004.dat  AS-2-2-bias+01_BK-1100_045fil_TI_006.dat  AS-2-2-bias+01_BK-1100_045fil_TI_007.dat  AS-2-2-bias+01_BK-1100_045fil_TI_008.dat | PCR positive saliva for training | Figs. 3a-3c, 3f, 3h, 3i, S13, S14, Table S5, Data S1 |
| Saliva(n=100 HO)_I-t data-20210209T093219Z-061.zip | test/Negative/HD-120420-21 | HD-120420-21_001.dat | PCR negative saliva for test | Figs. 3g, 3j, 3k S15, Table S6, Data S2 |

Sampling rate: 250 KHz. Setting.xml and CatalogFile.xml include measurement settings.
